# An ENIGMA Consortium study of the relationship between white matter microstructure and positive and negative symptom severity in patients with schizophrenia

**DOI:** 10.1038/s41537-026-00728-z

**Published:** 2026-03-06

**Authors:** Aoife Warren, Laurena Holleran, Ingrid Agartz, Ole A. Andreassen, Nerisa Banaj, Dara M. Cannon, Aiden Corvin, Melissa Green, Ruben Gur, Ryota Hashimoto, Elliot Hong, Cyril Hoschl, Peter Kochunov, Stephen M. Lawrie, Colm McDonald, Derek Morris, David Mothersill, Emma Neilson, Christos Pantelis, Fabrizio Piras, Paul E. Rasser, David Roalf, Theodore D. Satterthwaite, Ulrich Schall, Kang Sim, Antonin Skoch, Gianfranco Spalletta, Filip Spaniel, Sophia Thomopoulos, David Tomecek, Andrew Zalesky, Paul M. Thompson, Neda Jahanshad, Jessica A. Turner, Theo G. M. van Erp, Gary Donohoe

**Affiliations:** 1 School of Psychology, Centre for Neuroimaging, Cognition and Genomics (NICOG), & Galway Neuroscience Centre, University of Galway, Galway, Ireland; 2 NORMENT, KG Jebsen Centre for Psychosis Research, Division of Mental Health and Addiction, Oslo University Hospital and Institute of Clinical Medicine, University of Oslo, Oslo, Norway; 3 Department of Psychiatry, Ullevål University Hospital and Institute of Psychiatry, University of Oslo, Oslo, Norway; 4 Laboratory of Neuropsychiatry, Department of Clinical and Behavioral Neurology, IRCCS Santa Lucia Foundation, Rome, Italy; 5 Neuropsychiatric Genetics Research Group, Department of Psychiatry, Trinity College Dublin, Dublin, Ireland; 6 Systems Lab, Departments of Psychiatry & Biomedical Engineering, University of Melbourne, Melbourne, VIC Australia; 7 Department of Psychiatry, University of Pennsylvania, Philadelphia, PA USA; 8 Department of Pathology of Mental Diseases, National Institute of Mental Health, National Center of Neurology and Psychiatry, Kodaira, Tokyo Japan; 9 Maryland Psychiatric Research Center, Department of Psychiatry, University of Maryland School of Medicine, Baltimore, MD USA; 10 National Institute of Mental Health, Klecany, Czechia; 11 University of Maryland Center for Brain Imaging Research, University of Maryland, Baltimore, MD USA; 12 Department of Psychiatry, University of Edinburgh, Edinburgh, UK; 13 Department of Psychology, School of Business, National College of Ireland, Dublin, Ireland; 14 Centre for Brain & Mental Health Research, The University of Newcastle, Newcastle, NSW Australia; 15 Priority Research Centre for Stroke and Brain Injury, University of Newcastle, Callaghan, Newcastle, NSW Australia; 16 West Region, Institute of Mental Health, Singapore, Singapore; 17 Imaging Genetics Center, Keck School of Medicine, University of Southern California, Los Angeles, CA USA; 18 Department of Psychiatry & Behavioral Health, Wexner Medical Center, The Ohio State University, Columbus, OH USA; 19 Clinical Translational Neuroscience Laboratory, Department of Psychiatry and Human Behavior, University of California Irvine, Irvine, CA USA; 20 Center for the Neurobiology of Learning and Memory, University of California Irvine, Irvine, CA USA

**Keywords:** Schizophrenia, Schizophrenia

## Abstract

Symptom severity in schizophrenia has been repeatedly associated with thinner cortical gray matter. While global and regional white matter microstructure alterations in schizophrenia are well-documented, their association with clinical symptom severity remains unclear. As this is likely due to methodological heterogeneity across studies, we tested whether symptom severity in schizophrenia was associated with regional and global white matter alterations using standardized methods. We hypothesized that positive symptom severity would be associated with temporal white matter changes and that negative symptom severity would be associated with alterations in frontal white matter. Using a standardized fractional anisotropy (FA) analysis pipeline developed by the ENIGMA consortium, we conducted a meta-analysis of the association between white matter microstructure and symptom severity in *n* = 1025 (ages 16–68 years; 369 women/656 men) across 19 ENIGMA sites. Where significant heterogeneity was detected across sites, we examined whether variation in association strength between white matter microstructure and symptom severity was explained by duration of illness and/or current antipsychotic use. Positive symptom severity was significantly associated with white matter microstructure as measured using temporal lobe FA and global FA. Negative symptom severity showed no significant association with white matter microstructure as measured using frontal lobe FA or global FA. Significant heterogeneity across sites was observed for the negative symptom analysis, explained partly by duration of illness. Post-hoc exploratory analyses identified one site as disproportionately contributing to this heterogeneity, and when removed, negative symptom severity was significantly associated with both global and frontal FA. These findings support the view of schizophrenia as a disorder of brain connectivity, in a manner relevant to understanding variation in clinical symptom severity.

## Introduction

Structural magnetic resonance imaging (MRI) studies of schizophrenia (SZ) provide robust evidence of gray matter (GM) abnormalities^1^, including widespread reductions in GM volume^2^, cortical thinning^3^, and cortical surface area^4^. Such alterations are observed across frontal, temporal, occipital, and parietal regions in individuals with SZ when compared to healthy control subjects (HC)^1,2,4^.

Furthermore, diffusion tensor imaging (DTI) studies provide robust evidence of widespread white matter (WM) microstructural alterations in SZ in comparison to HC^5–7^. For example, in our previous case-control study^6^, we found evidence of significant differences in fractional anisotropy (FA) in 19 out of the 24 individual tracts investigated, such as the anterior corona radiata (ACR) and the corpus callosum (CC). However, the largest effect size for WM alterations between individuals with SZ and HC has been reported for the average WM skeleton^6^.

Structural brain changes in SZ have previously been linked to the severity of positive and negative symptoms. Previous evidence has linked higher positive symptom severity—most often auditory hallucinations—to cortical thinning in frontal and temporal brain regions^2,3,8^. Specifically, thinner GM in the superior temporal gyrus (STG) and the left middle temporal gyrus (MTG), key regions of the language network of the brain, have previously been associated with positive symptom severity^9,10^. Reduced FA in the WM of temporal regions involved in language comprehension and production have also been consistently reported in SZ^6,7,11^. The relationship between WM alterations and positive symptoms has been investigated in several small studies^12–14^; however, findings across studies have been inconsistent. Positive symptom severity has been associated with both lower FA^15,16^, and higher FA^17–20^. One consistent finding that has emerged is the significance of alterations in temporal WM tracts and temporal segments of tracts in relation to positive symptoms^15,21–24^.

The negative symptoms of SZ typically respond less well to antipsychotic treatment and are associated with a greater impact on functioning than positive symptoms^25^. Previous structural MRI studies of SZ have linked negative symptoms to cortical thinning^26^. This includes a study by the ENIGMA-SZ consortium, which found that cortical thinning in the left medial orbitofrontal cortex (mOFC) is associated with greater severity of negative symptoms^27^. Prefrontal regions, such as the mOFC, play key roles in emotional processing and the integration of emotional valence^28^, both of which are impaired in SZ^27^. Reduced FA in frontal WM^7,11^ and in WM tracts linked to the PFC and OFC^6^ is consistently reported in SZ compared to HC. Furthermore, aberrations in frontal WM tracts have previously been linked to negative symptoms^17,29–31^. However, the reported direction of this association is inconsistent across studies—some report a positive relationship between FA and negative symptoms^30,32^, while others have found an inverse relationship^24,31^. These discrepancies in past findings limit any definite characterization of the association between altered WM microstructure and negative symptom severity.

Of note, a previous large, multisite study (*n* = 597) found that greater abnormalities in WM microstructure were associated with higher overall symptom severity (across both positive and negative symptoms) and that longer duration of illness and higher antipsychotic dose were associated with greater deviations from normal WM structure^33^. These sources of between-site heterogeneity have been previously suggested to contribute to variation in both symptom severity and FA of WM tracts^1,24,34^.

Aside from this, previous studies have primarily focused on the association between symptoms and individual WM tracts within small, low-powered samples. However, given the evidence for widespread WM alterations in SZ^6,35^, and previous findings that the average WM skeleton shows the greatest effect size for deviations from healthy WM^6^, we speculated that regional alterations in FA (temporal and frontal, for positive and negative symptoms, respectively), as well as more global WM changes, would be associated with symptom severity in SZ. To our knowledge, a characterization of the association between global and regional WM alterations and the severity of positive (e.g., delusions, hallucinations^3^) or negative (e.g., diminished emotional expression, anhedonia, avolition^27^) symptoms using an international, well-powered sample has yet to be undertaken. To address this, the ENIGMA-SZ working group conducted a coordinated meta-analysis of the association between global and temporal or frontal WM microstructure and clinical symptom severity. DTI data from multiple international sites were processed using a harmonization platform (https://enigma.ini.usc.edu/protocols/dti-protocols), which outlines recommendations for pre-processing and TBSS analysis before outputs are harmonized using ENIGMA Harmonization (eharmonize).

Based on the evidence reviewed above, we hypothesized that positive symptom severity would be associated with regional WM alterations in temporo-parietal tracts, and negative symptom severity would be associated with regional alterations in frontal WM tracts. We also hypothesized that both positive and negative symptoms would be associated with global WM alterations, albeit to a lesser extent than the regional WM changes. We further explored the extent to which duration of illness and medication dosage accounted for any unexplained variance across sites.

## Results

### Demographic data

Demographic data (sex, age, symptom scale, duration of illness (in years), and chlorpromazine (CPZ) equivalents (per day) are presented in Table 1. For the positive symptom analysis, a total of *n* = 1025 participants with SZ were included across 19 ENIGMA sites. For the negative symptom analysis, 5 sites (ASRB1-5) were excluded due to limited assessment of negative symptomatology by the DIP (Diagnostic Inventory of Psychosis^36^), resulting in a final sample of *n* = 775 participants. The mean age for the total sample was 37 years (SD = 6.34 years) with a gender split of 64% males across all sites. The mean duration of illness was 13.3 years (SD = 6.6 years), and the mean CPZ equivalents per day was 498 mg (SD = 251.1 mg per day).

**Table 1:** Demographics for the ENIGMA schizophrenia sites (19 sites, *n* = 1025) included in the meta-analysis.

Site	*n*	Duration of illness (SD)	Scale	CPZ (SD)	Age (SD)	M/F %
ASRB1	102	15.7	DIP	N/A	39 (11)	74/26
ASRB2	76	14.5	DIP	N/A	38 (10)	64/36
ASRB3	14	18.2	DIP	N/A	41 (7)	71/29
ASRB4	6	15.8	DIP	N/A	38 (10)	72/28
ASRB5	52	17.3	DIP	N/A	40 (10)	66/34
Dublin	29	19.6	SAPs/SANs	340	44 (11)	74/26
Galway	13	7.7	PANSS	324	31 (11)	92/8
Osaka	76	11.5	PANSS	750	35 (11)	50/50
Rome	83	14.0	PANSS	417	39 (12)	66/34
TOP	69	6.8	PANSS	340	28 (8)	63/37
UPENN12	15	15.2	SAPs/SANs	380	36 (9)	67/33
UPENN64	34	12.8	SAPs/SANs	1019	34 (11)	59/41
HUBIN	37	28.4	PANSS	415	52 (8)	77/23
Singapore	85	6.0	PANSS	192	32 (10)	55/45
Edinburgh	26	13.6	PANSS	428	38 (10)	44/56
BCP	122	16.4	PANSS	473	41 (13)	46/53
iRELATE	39	17.9	PANSS	980	43 (11)	67/33
ESO	66	0.9	PANSS	311	31 (8)	58/42
Huilong	81	1.1	PANSS	600	24 (6)	51/49
**Total**	**1025**	**13.3 (6.6)**		**498 (251)**	**37 (6.34)**	**64/36**

ASRB1-5 were excluded from the negative symptom analysis due to limited assessment of negative symptoms by the DIP (14 sites remaining, *n* = 775).

*DIP* Diagnostic Inventory Of Psychosis, *SAPs/SANs* Scale for the Assessment of Positive/Negative Symptoms, *PANSS* Positive and Negative Syndrome Scale, *CPZ* chlorpromazine equivalents in mg/day, *M/F* males/females, *Duration of illness* time from diagnosis to data collection in mean years.

### Positive symptoms

#### Meta-analysis between temporal fractional anisotropy and positive symptoms

**Fig. 1:**
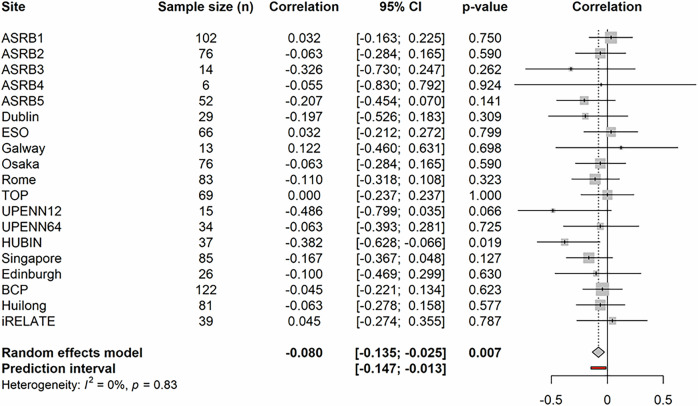
Forest plot for the association between temporal fractional anisotropy and positive symptoms. The meta-analysis results for positive symptoms and temporal-FA (*n* = 1025) showing a significant pooled association across 19 ENIGMA sites (*r* = −0.0802 [−0.1353, −0.0246], *p* = 0.007) with a non-significant degree of residual heterogeneity between sites (*p* = 0.83). Symbols used: *I*^2^ = Higgins and Thompson's *I*^2^ Statistic; CI = confidence interval.

A random effects meta-analysis was conducted using site-specific correlation coefficients for the association between temporal-FA and positive symptoms (*n* = 1025), which indicated a modest, significant negative correlation between temporal-FA and positive symptoms across all 19 sites (*r* = −0.0802 [−0.1353, −0.0246], *p* = 0.007, df = 18). A non-significant amount of heterogeneity was observed between sites (*p* = 0.83). Consequently, the meta-regression step was not performed. A forest plot showing the site-specific correlation coefficients, the corresponding 95% CI, and *p* values is presented in Fig. 1 (see accompanying table—Supplementary Table 5; and accompanying influence analyses—Supplementary Figs. 1–3).

#### Meta-analysis between global fractional anisotropy (gFA) and positive symptoms

**Fig. 2:**
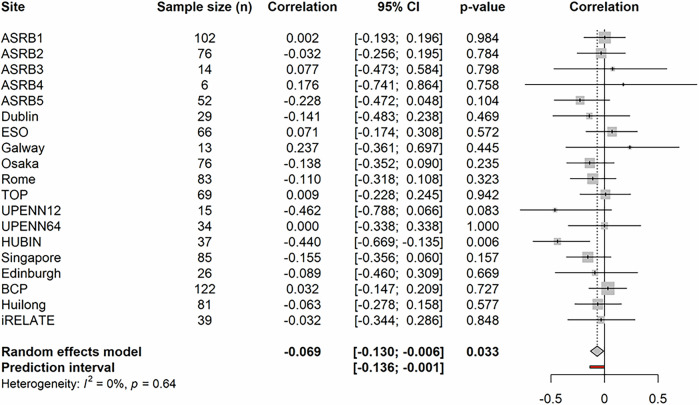
Forest plot for the association between global fractional anisotropy and positive symptoms. The meta-analysis results for positive symptoms and global-FA (*n* = 1025) showing a significant pooled association across 19 ENIGMA sites (*r* = −0.0686 [−0.1303, −0.0063], *p* = 0.0327) with a non-significant degree of residual heterogeneity between sites (*p* = 0.64). Symbols used: *I*^2^ = Higgins and Thompson's *I*^2^ Statistic; CI = confidence interval.

A random effects meta-analysis was conducted using the site-specific correlation coefficients for the association between gFA and positive symptoms (*n* = 1025), which indicated a modestly significant negative association between gFA and positive symptoms across all 19 sites (*r* = −0.0686 [−0.1303, −0.0063], *p* = 0.0327, df = 18). A non-significant amount of heterogeneity was observed between sites (*p* = 0.64). Therefore, the meta-regression step was not performed. A forest plot showing the site-specific correlation coefficients, the corresponding 95% CI, and *p* values is presented in Fig. 2 (see accompanying table—Supplementary Table 6; and accompanying influence analyses—Supplementary Figs. 4–6).

#### Meta-analysis between global fractional anisotropy, excluding temporal brain regions, and positive symptoms

We ran a meta-analysis for the association with gFA, excluding temporal brain regions, and positive symptoms to examine if the gFA component explains variance in positive symptoms beyond that captured by the temporal-FA component. The random effects model remained significant (*r* = −0.0635 [−0.1242, −0.0023], *p* = 0.0427, df = 18), with the amount of heterogeneity remaining non-significant (*p* = 0.67) (see Supplementary Fig. 7 for forest plot; Supplementary Table 7 for results; Supplementary Figs. 8–10 for influence analyses).

#### Post-hoc variance partitioning analysis

A post-hoc variance partitioning analysis was then performed to assess the unique contributions of the temporal-FA association and the gFA association, excluding temporal regions, to the broader association between gFA and positive symptoms. We ran a multiple regression analysis to include both temporal-FA and gFA, excluding temporal brain regions. The association with gFA, excluding temporal brain regions, was a significant predictor of the broader gFA association (*r* = 0.7702, *p* < 0.001, *R*^2^ = 27.3%), whereas the association between temporal-FA and positive symptoms was not (*r* = 0.2202, *p* = 0.163, *R*^2^ = 1.65%).

Taken alongside the results of the meta-analysis, these findings suggest that both temporal lobe alterations in FA and global changes in FA are uniquely associated with positive symptomatology.

### Negative symptoms

#### Meta-analysis between frontal fractional anisotropy and negative symptoms

**Fig. 3:**
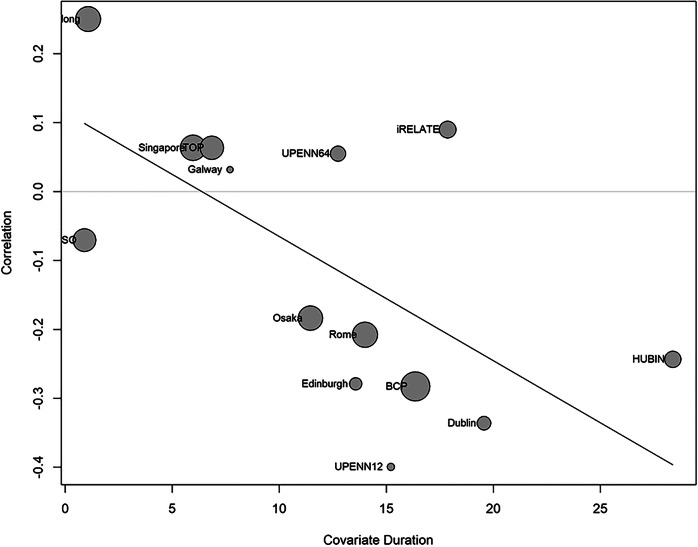
Bubble plot showing the relationship between duration of illness and the correlation between frontal fractional anisotropy and negative symptoms. The relationship between duration of illness and the size of the correlation coefficient for frontal-FA and negative symptoms at each site (*n* = 14) included in the meta-regression model (*p* = 0.0065, *I*^2^ = 6.9%), whereby longer duration of illness is associated with a stronger inverse association between frontal-FA and negative symptoms.

A random effects meta-analysis was carried out using the site-specific correlation coefficients for the association between frontal-FA and negative symptoms (*n* = 775), which indicated a non-significant pooled association for frontal-FA and negative symptoms across sites (*r* = −0.0861, [−0.1946, 0.0246], *p* = 0.117, df = 13). Low-to-moderate heterogeneity was noted between sites (*I*^2^ = 48%, *p* = 0.02) (see Supplementary Fig. 11 for forest plot; Supplementary Table 8 for results). Multiple meta-regression was used to estimate whether duration of illness and/or CPZ equivalents partially accounted for this residual heterogeneity. Mean duration of illness appeared to explain a majority of the residual heterogeneity in the model (*p* = 0.0065, *I*^2^ = 6.9%) (Supplementary Table 9A, B), whereas CPZ scores did not (*p* = 0.4451). Figure 3 illustrates the relationship between duration of illness and the size of the frontal-FA and negative symptom association across sites, whereby longer duration of illness is associated with a stronger inverse association between frontal-FA and negative symptoms.

Given the evidence that duration of illness explained much of the residual heterogeneity in the model, we carried out a post-hoc exploratory leave-one-out analysis to determine whether this heterogeneity was being driven by any one site (see Supplementary Figs. 12–14). This indicated that one site in particular (Huilong) was driving the observed heterogeneity. On omitting this site, heterogeneity in the model became non-significant (*I*^2^ = 21%, *p* = 0.23). Notably, the frontal-FA and negative symptom association became significant based on the remaining sites (*r* = −0.121, [−0.215, −0.025], *p* = 0.018, df = 12) (see Supplementary Fig. 15).

#### Meta-analysis between global fractional anisotropy and negative symptoms

**Fig. 4:**
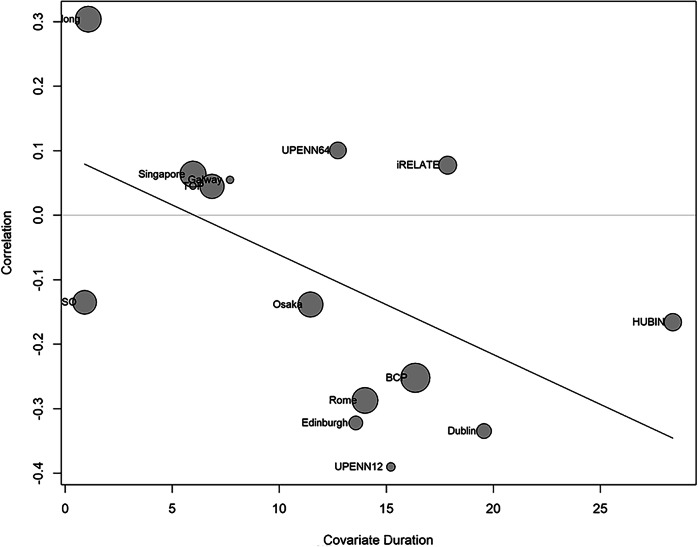
Bubble plot showing the relationship between duration of illness and the correlation between global fractional anisotropy and negative symptoms. The relationship between duration of illness and the size of the correlation coefficient between gFA and negative symptoms at each site (*n* = 14) included in the meta-regression model (*p* = 0.034, *I*^2^ = 32.45%), whereby longer duration of illness is associated with a stronger inverse association between gFA and negative symptoms.

A random effects meta-analysis was carried out using the site-specific correlation coefficients for the association between gFA and negative symptoms (*n* = 775), indicating a non-significant association between gFA and negative symptoms (r = −0.0084, [−0.1988, 0.0340], *p* = 0.148, df = 13), and low-to-moderate heterogeneity between sites (*I*^2^ = 54%, *p* < 0.01) (see Supplementary Fig. 16 for forest plot; Supplementary Table 10 for results). Using multiple meta-regression, the mean duration of illness was observed to account for a significant amount of heterogeneity (*p* = 0.034, *I*^2^ = 32.45%; Fig. 4 and Supplementary Table 11A, B), whereas CPZ did not (*p* = 0.2957).

Post-hoc exploratory leave-one-out analyses were carried out to identify any site(s) contributing disproportionately to the heterogeneity explained by duration of illness (see Supplementary Figs. 17–19). This indicated that the heterogeneity was driven by one particular site (Huilong). On omitting Huilong, duration of illness no longer explained the residual heterogeneity (*p* = 0.1615), and the association between gFA and negative symptoms became significant based on the remaining sites (*r* = −0.124, [−0.218, −0.027], *p* = 0.017, df = 12) (see Supplementary Fig. 20).

#### Meta-analysis between global fractional anisotropy (excluding frontal brain regions) and negative symptoms

A random effects meta-analysis was carried out with the site-specific correlation coefficients for the association between gFA, excluding frontal brain regions, and negative symptoms (*n* = 775), indicating a non-significant pooled association across the 14 included sites (*r* = −0.0521, [−0.1594; 0.0565], *p* = 0.3192, df = 13). Low-to-moderate heterogeneity was observed between sites (*I*^2^ = 45%, *p* = 0.03) (see Supplementary Fig. 21 for forest plot; Supplementary Table 12 for results). Using multiple meta-regression, the mean duration of illness showed a trend towards explaining this residual heterogeneity (*p* = 0.0799, *I*^2^ = 30.6%), whereas CPZ scores did not (*p* = 0.2035) (see Supplementary Fig. 22 for bubble plot; Supplementary Table 13A, B for results).

A post-hoc exploratory leave-one-out analysis was performed to examine if any site(s) were driving the residual heterogeneity, which showed that one site (Huilong) was the sole driver of this heterogeneity (see Supplementary Figs. 23–25). On omitting Huilong, the residual heterogeneity reduced to non-significant levels (*p* = 0.51), and the association between gFA excluding frontal regions and negative symptoms became significant based on the remaining sites (*r* = −0.0943 [−0.1753, −0.0121], *p* = 0.0280, df = 12) (see Supplementary Fig. 26).

#### Post-hoc variance partitioning analysis

We ran a multiple regression model (excluding Huilong) to examine if the gFA component explained variation in negative symptoms, beyond that captured by the frontal-FA component. Both the association between gFA excluding frontal brain regions and negative symptoms (*r* = 0.5888, *p* < 0.001, *R*^2^ = 5.14%), and the association between frontal-FA and negative symptoms (*r* = 0.5026, *p* < 0.001, *R*^2^ = 4.46%), significantly predicted the gFA association.

Taken alongside the meta-analysis results, these findings suggest that both the frontal-FA and gFA components uniquely contribute to explaining variance in negative symptom severity.

## Discussion

This study sought to characterize the relationship between WM microstructure and clinical symptom severity in *n* = 1025 patients with SZ, using a standardized ENIGMA-DTI analysis pipeline. In analyses where significant residual heterogeneity between sites was detected, we further sought to determine whether either duration of illness or medication dosage accounted for this variance.

We observed that variation in WM microstructure was significantly negatively associated with positive symptom severity, i.e., *higher* symptom severity was associated with *lower* gFA and *lower* temporal-FA. A post-hoc partitioning analysis was performed to assess the unique contributions of the temporal-FA and gFA (excluding temporal brain regions) components in explaining variance in positive symptom severity. The findings of this analysis suggested that the gFA component captured variance in positive symptoms beyond that of the temporal component.

For negative symptoms, no association was observed for either gFA or frontal-FA (the specific region hypothesized) in the meta-analysis model when all eligible sites were included. Significant residual heterogeneity was observed between sites, which was accounted for by differences in duration of illness, but not medication dosage. We found that where the mean duration of illness was longer, the association between negative symptoms and FA trended towards being larger. In post-hoc exploratory analyses, one site (Huilong) was observed to contribute disproportionately to the residual heterogeneity across sites in the associations between FA and negative symptoms. On removal of this site, significant associations were observed between *higher* negative symptom severity and *lower* gFA and frontal-FA.

### Positive symptomatology and the integrity of individual white matter tracts

We had hypothesized that temporo-parietal tracts^15,16^ would be particularly associated with positive symptom severity. This was based on their role in processes relevant to positive symptoms, including auditory processing, language production, and self-monitoring, and its previous associations with SZ pathophysiology^37,38^. Our hypothesis was further supported by the prominence of temporal tracts in studies reporting a significant association between FA and positive symptoms^15,21–24^. Instead, we found that positive symptom severity was associated more broadly with global WM changes. This is consistent with our earlier study, where patients showed widespread reductions in FA in individual tracts when compared to healthy controls^6^. We further showed that cognitive ability is associated with global FA alterations across patients with SZ and control subjects^35^.

The direction of the observed association between positive symptoms and both temporal and gFA is also of note. Our finding that higher symptom severity is associated with lower gFA is consistent with some previous reports^18^, while others have suggested that positive symptoms (either individually, e.g., auditory hallucinations, or total positive symptom scores) are instead associated with higher FA^19,20,25,39^. The findings from the current study suggest that aberrant cortical communication may reflect reductions in WM integrity, as opposed to increased structural connectivity, in its association with clinical symptom severity in chronic SZ. Overall, our findings are consistent with the view that SZ represents a disorder of widespread, rather than focal, structural dysconnectivity.

### Negative symptomatology and the integrity of individual white matter tracts

Findings from previous studies examining FA and negative symptoms have been inconsistent, with significant associations reported by some studies^39–42^, but not others^12,18,41^. Notably, the majority of previous studies investigated the associations between negative symptoms and FA of individual WM tracts and did not examine their relationship with global WM changes. In one case, negative symptoms were found to be negatively associated with FA predominantly in prefrontal brain regions (CR, internal and external capsule, inferior fronto-occipital fasciculus (IFOF), for example) in a sample of patients with SZ spectrum disorders^43^. Whereas, in contrast, in ultra-high risk individuals, an increase in negative symptoms was correlated with an increase in FA in the right superior longitudinal fasciculus (SLF)^30^. Such inconsistent findings are likely due to small sample sizes, differing patient groups (ultra-high risk, first-episode, chronic), and varying methodologies.

In addition, our findings suggest that the inconsistent findings reported to date may reflect the influence of the duration of illness. This suggestion is in line with a finding from a previous large-scale study in SZ, which reported that longer duration of illness is associated with more severe aberrations in WM structure compared to healthy participants^33^. Our findings imply that residual heterogeneity in the association between negative symptom severity and FA relates in some capacity to duration of illness, whereby *longer* duration of illness was associated with a *stronger* inverse association between gFA and negative symptoms. Reducing heterogeneity associated with duration of illness (by removing one site that was an outlier for illness duration) revealed significant associations with both gFA and frontal-FA. Given that these associations were only observed following an exploratory analysis that included the removal of that site, these findings should be interpreted cautiously.

### Strengths and limitations

This meta-analysis study analyzed the relationship between WM and symptom severity using a well-validated and harmonized ENIGMA-DTI analysis pipeline across a large sample of *n* = 1025 individuals with SZ. The study overcomes several significant limitations of previous studies by minimizing or modeling sources of heterogeneity and reducing the potential for consequent false positive or negative findings. The large, multisite sample allows for sufficient power to detect relatively small effects, which may have been overlooked in a smaller sample with significantly lower power.

A number of study limitations should be considered when interpreting our findings. First, in an attempt to minimize the multiple testing burden associated with analysis of individual WM tracts, symptom sub-scales, or item-level data for symptoms, we decided to apply principal components analysis (PCA) to summarize FA using global, frontal, and temporal components, and to focus on total positive and negative symptom scores. As a result, we are only able to make more general observations about the association between WM microstructure and symptom severity. Secondly, there were only three samples in our study that had a mean illness duration of less than 5 years, meaning that the study cohort overall is more representative of a chronic SZ sample. Thirdly, while including diverse cultures enhances the generalizability of findings, it also introduces variability in clinical assessments, treatment practices, and healthcare systems. This may partially explain why a Chinese site (Huilong) emerged as an outlier in the negative symptom analysis, amongst other sites which are predominantly located in the USA and Europe. While this highlights the challenges of culturally diverse, international large-scale studies, it also emphasizes the importance of cultural context when interpreting findings in psychiatric research. Lastly, the cross-sectional nature of the study impacts the reliability of variables such as medication type and dosage, and symptom status. CPZ equivalents do not account for medication history, and do not distinguish between first- and second-generation antipsychotics, which have previously been shown to differ in their effects on brain structure^44–46^. Hence, using CPZ equivalents to estimate drug dosage is likely to have limited our ability to establish the effects of medication on WM. Furthermore, cross-sectional observations of symptom data are confounded by episode stage and type at data collection. Symptom status might also impact FA measurement—for example, individuals in the early remission phase may be undergoing dynamic brain changes that impact FA, such as swelling of myelin^47–49^.

Future studies should consider the need for large sample sizes and the harmonization of analysis pipelines in the study of WM microstructure and its association with clinical symptomatology. Based on previous studies highlighting the utility of FA in detecting control vs patient group differences in WM structure, we chose to solely focus on FA as the measure of WM integrity. However, future studies will benefit from incorporating other DTI metrics, such as mean, radial, and axial diffusivity, when possible. These alternative measures may provide further insights into the pathology relating to WM. Finally, the value of longitudinal data collection should also be considered, particularly pertaining to measures that vary rapidly and significantly over time, such as symptom severity, medication usage, and dosage.

## Conclusion

In the largest study of the relationship between WM microstructure and clinical symptom severity to date, we provide evidence of a modest association between global alterations in WM microstructure and increased positive symptom severity in patients with SZ. In modeling the association between WM microstructure and negative symptoms, we found that duration of illness was an important consideration. Finally, the associations observed between symptom severity and WM microstructure are consistent with an understanding of SZ as a disorder of brain connectivity, in a manner relevant to understanding variation in clinical severity.

## Methods

### Study sample

Data for the current study were collected via the ENIGMA-SZ-DTI working group. The participating sites included research centers in Ireland, the United States, Japan, Australia, Italy, Norway, Singapore, Scotland, the Czech Republic, and China. Inclusion criteria included the availability of data processed using the ENIGMA-DTI protocol and measures of SZ symptom severity for each participant in each dataset. Demographic information collected included illness duration, chlorpromazine (CPZ) equivalents, age, and sex. The final sample consisted of 1025 patients with SZ (Table 1). Each study sample was assessed with participants’ written informed consent, after study approval by local Institutional Review Boards.

### Symptoms rating scales in schizophrenia

Symptom severity was assessed using the Positive and Negative Syndrome Scale^50^ (PANSS, 11 sites), Scale for the Assessment of Positive/Negative Symptoms^51,52^ (SAPS/SANS, 3 sites), or Diagnostic Inventory of Psychosis^36^ (DIP, 5 sites (ASBR1-5)). Symptoms were assessed during the data collection period for all participants and therefore reflect each participant’s episode type and stage at this time. Sites using the DIP (ASRB1-5) were excluded from the negative symptom analysis due to the limited assessment of negative symptoms by the DIP. For each site, PANSS, SAPS/SANS, or DIP scales were analyzed to assess their association with WM microstructure. Symptom conversion was carried out using the equations outlined previously by van Erp et al.^53^ (see Supplementary Methods for conversion equations). These linear regression-based conversion algorithms allowed for conversion of symptom dimension scores between (a) SAPS and PANSS-positive scales for positive symptomatology and (b) SANS and PANSS-negative scales for negative symptomatology.

### Image acquisition and processing

Imaging data were acquired using site-specific diffusion MRI sequences. Details of study type, scanner, and acquisition parameters for each site are presented in Supplementary Table 1. For each site, pre-processing, including eddy current correction, echo-planar imaging-induced distortion correction, and tensor fitting, was carried out locally based on local protocols and procedures, and further informed by quality control pipelines available via the ENIGMA-DTI webpage (http://enigma.ini.usc.edu/protocols/dti-protocols) and NITRC (Neuroimaging Informatics Tools and Resources Clearinghouse; https://www.nitrc.org/projects/enigma_dti/). To correct for subject motion during image acquisition, pre-processing included the alignment of diffusion-weighted images to the *b* = 0 (non-diffusion-weighted) image using linear image registration. Individuals with bad-quality diffusion images (based on a visual check of each FA map prior to TBSS, see linked protocol above) were excluded from the analysis. As per Kelly et al.^6^, harmonization of pre-processing schemes was not enforced across sites to allow individual sites to use existing pipelines that may be more appropriate for their data acquisition. Following pre-processing, harmonized image analysis was conducted to compute the FA measures at each site using the ENIGMA-DTI protocol (http://enigma.ini.usc.edu/protocols/dti-protocols/). The ENIGMA-DTI protocol using TBSS^54^ outputted averaged FA for all WM regions of interest (Supplementary Table 2). The TBSS output includes FA values for both right and left bilateral WM tracts and an average FA based on values from both hemispheres. For this analysis, bilateral average FA values for all tracts were used, minimizing multiple comparisons. Secondary TBSS metrics, including mean, radial, and axial diffusivity, were not included as previous findings suggest that they possess less significance when analyzing SZ-HC group differences^6,35^. Further to this, their biological interpretation is limited, and their measurement is affected by processes such as inflammation and edema^55,56^.

### Statistical analysis

#### Within-site analysis

Calculation of FA is based on specific acquisition protocols, including scanner make and model, diffusion sequence parameters, methods of tensor estimation models, and processing pipelines^57,58^. To overcome this systematic limitation, preliminary analysis was carried out individually at each site to assess the association between WM tract microstructure and positive/negative symptoms. For the meta-analysis, the input parameters were correlation coefficients summarizing the association between FA and symptom severity at each site. This reduced the variance introduced by the symptom scale, scanner, or acquisition parameters used at different sites.

#### Fractional anisotropy principal components analysis

To reduce the burden of multiple testing, we undertook PCA of FA from the available WM tracts to capture widespread signatures of WM effects. For each site separately, PCA, implemented in SPSS, was used to derive an unrotated first principal component, representing global WM, termed “gFA”^35^ (see Supplementary Table 2. for all included tracts).

Previous evidence has suggested that tracts located in or near the temporal lobe are of particular importance when investigating the links between FA and positive symptom severity^15,21–24^. Taking these findings alongside the reported association between thinner STG and MTG cortices and positive symptoms^9,18^, we used a PCA to generate a latent factor capturing the common variance from the selected temporal-related WM tracts, which we hereafter refer to as temporal-FA. The included tracts were the cingulum bundle, inferior longitudinal fasciculus, uncinate fasciculus (UF), and splenium of the CC. It is important to note that while we aimed to capture variance across temporal tracts specifically, it is likely that the temporal-FA component captures some non-temporal variance. This is due to the inclusion of certain tracts, such as the splenium of the CC and the cingulum bundle, which possess extensive extra-temporal connections.

Similarly, previous studies have highlighted the role of frontal WM tracts in the association between negative symptoms and FA^17,29–31^. Combined with the previously reported association between thinning of the frontal cortex and negative symptom severity in SZ^27^, we derived a latent factor using PCA to summarize common variance across the frontal tracts of interest, subsequently referred to as frontal-FA. The PCA of frontal WM tracts included the ACR, anterior limb of the internal capsule, body and genu of the CC, fornix, SLF, IFOF, and the UF. These temporal-FA and frontal-FA WM tracts were based on TBSS ENIGMA output in consultation with a DTI WM atlas^59^. Furthermore, the calculation of these components followed a similar procedure to that used in prior publications from our group^35^ and others^60,61^. For each PCA, we examined scree plots and the extraction values. Comparable scree plots were observed for data across all sites for both temporal-FA and frontal-FA, suggesting that tract-FA values could be summarized by a single latent factor. The loadings of each WM tract on the latent components are presented in Supplementary Tables 3 and 4.

#### Within-site regression analyses

Regression analyses were performed at each individual site to estimate the variance in (1) positive symptoms explained by temporal-FA, and (2) negative symptoms explained by frontal-FA (IBM SPSS Statistics for Windows, version 24). Given previous reports that WM abnormalities in SZ can vary with age and sex^33^, we controlled for mean age and sex at a site-by-site level in each regression analysis. The resulting correlation coefficients between FA and symptom measures from each site were then entered into a meta-analysis to compute a pooled correlation coefficient summarizing the relationship between FA and symptoms across all sites.

#### Meta-analysis

Separate random effects meta-analyses were performed for positive and negative symptoms. All meta-analyses were carried out in RStudio IDE using R Statistical Software (v4.4.0)^62^ through the meta package (v7.0.0)^63^. The estimators and variances chosen for the meta-analysis models were comparable to the approach taken in our previous studies using the Comprehensive Meta-Analysis Software (see Supplementary Notes). The meta-analyses aimed to summarize the associations between symptom severity and FA across all eligible sites.

#### Meta-regression

In cases where significant residual heterogeneity was observed between sites in the meta-analysis (defined as *p* value < 0.05), we performed step-wise multiple meta-regression analyses (see Supplementary Notes for procedure), including mean duration of illness and/or CPZ equivalents (RStudio IDE; metafor package; v4.6.0^64^). This aimed to account for variables that may potentially obscure the relationship between WM integrity and symptom severity.

### **Inclusion and ethics statement**

All ENIGMA-SZ and ENIGMA-DTI sites included in this study followed the ENIGMA Memorandum of Understanding guidelines. This study was conducted in accordance with the Declaration of Helsinki, and study approval was received at each site from the local Institutional Review Board. Each study sample was assessed with participants’ written informed consent.

## Supplementary information

Supplementary Materials for the Manuscript

Supplementary File with Information of ENIGMA Members

## Data Availability

The data that support the findings of this study are available from the corresponding author (G.D.) upon reasonable request.

## Code availability

The codes supporting the findings of this study are available from the first author (A.W.) upon reasonable request.
