# Supplementary material for: An ENIGMA Consortium study of the relationship between white matter microstructure and positive and negative symptom severity in patients with schizophrenia
Source: Schizophrenia (Heidelb). 2026 Mar 6;12(1):38. doi: 10.1038/s41537-026-00728-z (PMC13087269; doi:10.1038/s41537-026-00728-z)

**Supplementary Materials**

**Supplementary Methods**

**Note.** Symptom Score Conversion Algorithms

1. **Positive Symptom Conversion Equations**

Global SAPS (Summary) Score = -3.222 + (0.567*PANSS Positive score)

Global SAPS (Summary) Score = 2.3526 + (0.1932*Total SAPS (composite) score)

PANSS: (-3.222) + (0.567 * PANSSPOS)

SAPS: (2.3526) + (0.1932 * SAPSTOT)

1. **Negative Symptom Conversion Equations**

Global SANS (Summary) Score = -2.0671 + (0.665*PANSS Negative score)

Global SANS (Summary) Score = 1.0863 + (0.2943*Total SANS (composite) score)

PANSS: (-2.0671) + (0.665 * PANSSNEG)

SANS: (1.0863) + (0.2943 * SANSTOT)

**Note.** Specifics for the Meta-Analysis Model

Specifics of the meta-analysis random effects model are as follows:

- Inverse variance method
- DerSimonian-Laird estimator for tau^2^
- Jackson method for confidence interval of tau^2^ and tau
- Hartung-Knapp adjustment for random effects model

**Note.** Procedure for the Step-wise Multiple Meta-regression Analysis

To account for significant residual heterogeneity in the meta-analysis models, we conducted step-wise multiple meta-regression. First, we run preliminary simple (univariate) meta-regressions using mean duration of illness or mean CPZ equivalents as moderator to determine which variable better accounted for inter-site heterogeneity. We found that mean duration of illness explained a greater proportion of the residual heterogeneity than mean CPZ equivalents. Accordingly, the first meta-regression model included mean duration of illness as the single moderator. We then re-ran the model including both mean duration of illness and mean CPZ equivalents as moderators. An ANOVA (likelihood-ratio test) was used to compare model fit and the proportion of heterogeneity explained between the single- and two-moderator models. If the two-moderator model explained significantly more residual heterogeneity (i.e., a significant improvement in model fit), this model was retained; otherwise, the simpler single moderator model was reported.

**Supplementary Table 1.** The scanner details and acquisition parameters for the ENIGMA sites included in the meta-analysis.

| **Study cohort** | **Scanner** | **Field Strength** | **Acquisitions** | **Voxel Size and Slice thickness (mm)** | **Gradient directions and b-value (mm/s2)** | **b=0 scans** |
| --- | --- | --- | --- | --- | --- | --- |
| ASRB 1 | Siemens Avanto | 1.5T | 1 | 2.4x2.4x2.4 | 64 at b =1000 | 1 |
| ASRB 2 | Siemens Avanto | 1.5T | 1 | 2.4x2.4x2.4 | 64 at b =1000 | 1 |
| ASRB 3 | Siemens Avanto | 1.5T | 1 | 2.4x2.4x2.4 | 64 at b =1000 | 1 |
| ASRB 4 | Siemens Avanto | 1.5T | 1 | 2.4x2.4x2.4 | 64 at b =1000 | 1 |
| ASRB 5 | Siemens Avanto | 1.5T | 1 | 2.4x2.4x2.4 | 64 at b =1000 | 1 |
| Dublin | Philips Achieva | 3T | 1 | 2x2x2.3 | 15 at b=800 | 1 |
| Galway | Siemens Magnetom Symphony | 1.5T | 1 | 2.5x2.5x2.5 | 64 at b=1300 | 7 |
| Osaka | GE | 3T | 1 | 1.0156x1.0156x33 | 15 at b=1000 | 1 |
| Rome | Siemens Allegra | 3T | 3 | 1.8x1.8x1.8 | 30 at b = 1000 | 2 |
| TOP | GE | 3T | 1 | 2x2x2.5 | 30 at b=1000 | 1 |
| UPENN12 | Siemens Trio | 3T | 1 | 1.72x1.72x3 | 12 at b=1000 | 1 |
| UPENN64 | Siemens Trio | 3T | 1 | 1.875x1.875x2.0 | 64 at b=1000 | 1 |
| HUBIN | GE | 3T | 1 | 0.94 x 0.94 x 2.9 | 60 at b=1000 | 10 |
| Singapore | Philips Achieva | 3T | 3 | 3x3x3 | 15 at b=800 | 1 |
| EDIN | Siemens Magnetom Verio | 3T | 1 | 2.2x2.5x2.5 | 56 at b=1000 | 6 |
| BCP | Siemens Prisma | 3T | 1 per shell | 1.5x1.5x1.5 | 93 at b=1000;92 at b=2000 | 14 |
| iRELATE | Philips Achieva MR system | 3T | 1 | 2x2x2 | 32 at b=1000 | 1 |
| ESO | Siemens Trio | 3T | 2 | 2x2x2 | 30 at b=900 | 2 |
| Huilong | Siemens Trio; GE | 3T | 1 AP, shimmed EPI | 1.8x1.8x3;1.0x1.0x3 | 64 at b=1000;32 at b=1000 | 1;3 |

**Supplementary Table 2.** The tracts included in the global fractional anisotropy principal component, the temporal fractional anisotropy latent component, and the frontal fractional anisotropy latent component. *Note.* These temporal-FA and frontal-FA white tracts were based on TBSS ENIGMA output in consultation with a DTI white matter atlas.

| **All Tracts** | **Temporal-FA Tracts** | **Frontal-FA Tracts** |
| --- | --- | --- |
| Full skeleton average FA | Cingulum cingulate gyrus (CGC) | Anterior corona radiata (ACR) |
| Anterior corona radiata (ACR) | Cingulum hippocampal portion (CGH) | Anterior limb of internal capsule (ALIC) |
| Anterior limb of internal capsule (ALIC) | Inferior fronto-occipital fasciculus (IFO) | Body of corpus callosum (BCC) |
| Body of corpus callosum (BCC) | Uncinate fasciculus (UNC) | Cingulum cingulate gyrus (CGC) |
| Corpus callosum (CC) | Splenium of corpus callosum (SCC) | Fornix (FX) |
| Cingulum cingulate gyrus (CGC) |  | Fornix - crus / Stria terminalis (FXST) |
| Cingulum hippocampal portion (CGH) |  | Genu of corpus callosum (GCC) |
| Corona radiata (CR) |  | Inferior fronto-occipital fasciculus (IFO) |
| Corticospinal tract (CST) |  | Superior fronto-occipital fasciculus (SFO) |
| External capsule (EC) |  | Superior longitudinal fasciculus (SLF) |
| Fornix (FX) |  | Uncinate fasciculus (UNC) |
| Fornix - crus / Stria terminalis (FXST) |  |  |
| Genu of corpus callosum (GCC) |  |  |
| Internal capsule (IC) |  |  |
| Inferior fronto-occipital fasciculus (IFO) |  |  |
| Posterior corona radiata (PCR) |  |  |
| Posterior limb of internal capsule (PLIC) |  |  |
| Posterior thalamic radiation (PTR) |  |  |
| Retrolenticular part of internal capsule (RLIC) |  |  |
| Splenium of corpus callosum (SCC) |  |  |
| Superior corona radiata (SCR) |  |  |
| Superior fronto-occipital fasciculus (SFOF) |  |  |
| Superior longitudinal fasciculus (SLF) |  |  |
| Sagittal stratum (SS) |  |  |
| Uncinate fasciculus (UNC) |  |  |

**Supplementary Table 3.** The loadings from the principal component analysis (FA) for the temporal fractional anisotropy latent component. *Note.* CGC = Cingulum cingulate gyrus, CGH = Cingulum hippocampal portion, IFO = inferior fronto-occipital fasciculus, UNC = uncinate fasciculus, SCC = splenium of corpus callosum.

| **Tracts** | **ASRB1** | **ASRB2** | **ASRB3** | **ASRB4** | **ASRB5** | **Dublin** | **Galway** | **Osaka** | **Rome** | **TOP** |
| --- | --- | --- | --- | --- | --- | --- | --- | --- | --- | --- |
| CGC | 0.853 | 0.758 | 0.749 | 0.819 | 0.824 | 0.778 | 0.838 | 0.795 | 0.89 | 0.742 |
| CGH | 0.515 | 0.656 | 0.575 | 0.591 | 0.555 | 0.471 | 0.71 | 0.491 | 0.816 | 0.513 |
| IFO | 0.565 | 0.648 | 0.578 | 0.607 | 0.605 | 0.599 | 0.636 | 0.653 | 0.654 | 0.552 |
| UNC | 0.526 | 0.467 | 0.652 | 0.641 | 0.357 | 0.426 | 0.366 | 0.344 | 0.766 | 0.541 |
| SSC | 0.812 | 0.82 | 0.812 | 0.775 | 0.801 | 0.786 | 0.804 | 0.778 | 0.897 | 0.74 |

| **UPENN12** | **UPENN64** | **HUBIN** | **Singapore** | **Edin** | **BCP** | **iRELATE** | **ESO** | **Huilong** |
| --- | --- | --- | --- | --- | --- | --- | --- | --- |
| 0.775 | 0.859 | 0.643 | 0.826 | 0.798 | 0.811 | 0.745 | 0.811 | 0.832 |
| -0.354 | 0.611 | 0.637 | 0.843 | 0.789 | 0.564 | 0.685 | 0.681 | 0.746 |
| 0.398 | 0.666 | 0.565 | 0.974 | 0.761 | 0.63 | 0.621 | 0.681 | 0.717 |
| 0.49 | 0.515 | 0.61 | 0.944 | 0.671 | 0.696 | 0.714 | 0.517 | 0.584 |
| 0.737 | 0.786 | 0.778 | 0.853 | 0.698 | 0.841 | 0.722 | 0.777 | 0.773 |

**Supplementary Table 4.** The loadings from the principal component analysis (PCA) for the frontal fractional anisotropy latent component. *Note.* ACR = Anterior corona radiata, ALIC = Anterior limb of internal capsule, BCC = Body of corpus callosum, CGC = Cingulum cingulate gyrus, FX = Fornix, FXST = Fornix - crus / Stria terminalis, GCC = Genu of corpus callosum, IFO = Inferior fronto-occipital fasciculus, SFO = Superior fronto-occipital fasciculus, SLF = Superior longitudinal fasciculus, UNC = Uncinate fasciculus.

| **Tracts** | **Dublin** | **Galway** | **Osaka** | **Rome** | **TOP** | **UPENN12** | **UPENN64** | **HUBIN** |
| --- | --- | --- | --- | --- | --- | --- | --- | --- |
| ACR | 0.852 | 0.588 | 0.772 | 0.761 | 0.768 | 0.806 | 0.845 | 0.84 |
| ALIC | 0.723 | 0.659 | 0.757 | 0.767 | 0.641 | 0.749 | 0.798 | 0.654 |
| BCC | 0.811 | 0.719 | 0.795 | 0.879 | 0.804 | 0.778 | 0.837 | 0.717 |
| CGC | 0.708 | 0.946 | 0.798 | 0.893 | 0.733 | 0.774 | 0.854 | 0.69 |
| FX | 0.538 | 0.609 | 0.454 | 0.584 | 0.319 | 0.271 | 0.814 | 0.428 |
| FXST | 0.576 | 0.939 | 0.578 | 0.765 | 0.462 | 0.499 | 0.669 | 0.61 |
| GCC | 0.845 | 0.777 | 0.873 | 0.848 | 0.786 | 0.847 | 0.896 | 0.829 |
| IFO | 0.649 | 0.429 | 0.434 | 0.593 | 0.466 | 0.172 | 0.559 | 0.421 |
| SFO | 0.776 | 0.657 | 0.699 | 0.763 | 0.686 | 0.685 | 0.725 | 0.677 |
| SLF | 0.825 | 0.753 | 0.801 | 0.824 | 0.702 | 0.704 | 0.712 | 0.737 |
| UNC | 0.521 | 0.453 | 0.425 | 0.672 | 0.442 | 0.504 | 0.392 | 0.537 |

| **Singapore** | **Edin** | **BCP** | **iRELATE** | **ESO** | **Huilong** |
| --- | --- | --- | --- | --- | --- |
| 0.821 | 0.762 | 0.841 | 0.867 | 0.820 | 0.782 |
| 0.83 | 0.845 | 0.794 | 0.695 | 0.777 | 0.879 |
| 0.675 | 0.746 | 0.825 | 0.86 | 0.727 | 0.857 |
| 0.84 | 0.774 | 0.787 | 0.774 | 0.709 | 0.858 |
| 0.609 | 0.595 | 0.666 | 0.689 | 0.566 | 0.675 |
| 0.675 | 0.768 | 0.66 | 0.708 | 0.627 | 0.746 |
| 0.625 | 0.893 | 0.88 | 0.875 | 0.759 | 0.854 |
| 0.934 | 0.526 | 0.498 | 0.499 | 0.602 | 0.735 |
| 0.762 | 0.753 | 0.728 | 0.814 | 0.686 | 0.77 |
| 0.799 | 0.677 | 0.644 | 0.768 | 0.759 | 0.849 |
| 0.91 | 0.621 | 0.567 | 0.54 | 0.411 | 0.555 |

**Supplementary Table 5**. Summary of the meta-analysis results for positive symptoms and temporal-FA, showing a significant inverse association across all 19 sites (*r* = -0.0802, [-0.1353, -0.0246], *p* = 0.007), with a non-significant amount of residual heterogeneity (*p* = 0.83). *Note.* *r*=site correlation value, LLCI=lower-level confidence interval, ULCI=upper-level confidence interval, %W=percentage weight.

| **Site** | ***n*** | ***r*** | **LLCI** | **ULCI** | ***p*** | **%W** |
| --- | --- | --- | --- | --- | --- | --- |
| ASRB1 | 102 | 0.032 | -0.1635 | 0.2251 | 0.75 | 10.2 |
| ASRB2 | 76 | -0.063 | -0.2844 | 0.1648 | 0.59 | 7.5 |
| ASRB3 | 14 | -0.326 | -0.7303 | 0.2474 | 0.262 | 1.1 |
| ASRB4 | 6 | -0.055 | -0.8295 | 0.7919 | 0.924 | 0.3 |
| ASRB5 | 52 | -0.207 | -0.4542 | 0.0698 | 0.141 | 5.1 |
| Dublin | 29 | -0.197 | -0.5256 | 0.1827 | 0.309 | 2.7 |
| ESO | 66 | 0.032 | -0.2117 | 0.2719 | 0.799 | 6.5 |
| Galway | 13 | 0.122 | -0.4599 | 0.6306 | 0.698 | 1 |
| Osaka | 76 | -0.063 | -0.2844 | 0.1648 | 0.59 | 7.5 |
| Rome | 83 | -0.11 | -0.3181 | 0.1083 | 0.323 | 8.3 |
| TOP | 69 | 0.00 | -0.2367 | 0.2367 | 1.000 | 6.800 |
| UPENN12 | 15 | -0.486 | -0.7993 | 0.035 | 0.066 | 1.2 |
| UPENN64 | 34 | -0.063 | -0.3928 | 0.2812 | 0.725 | 3.2 |
| HUBIN | 37 | -0.382 | -0.6283 | -0.0662 | 0.019 | 3.5 |
| Singapore | 85 | -0.167 | -0.3671 | 0.0478 | 0.127 | 8.5 |
| Edinburgh | 26 | -0.1 | -0.4692 | 0.2989 | 0.63 | 2.4 |
| BCP | 122 | -0.045 | -0.221 | 0.1338 | 0.623 | 12.3 |
| Huilong | 81 | -0.063 | -0.2775 | 0.1575 | 0.577 | 8.1 |
| iRELATE | 39 | 0.045 | -0.2744 | 0.3555 | 0.787 | 3.7 |
| **Overall** | 1025 | -0.0802 | -0.1353 | -0.0246 | 0.007 | 100 |

**Supplementary Figure 1.** Baujat plot for the analysis between positive symptoms and temporal-FA, showing the relative influence of each site on the pooled association (y-axis) and the overall heterogeneity (x-axis) across sites.

**
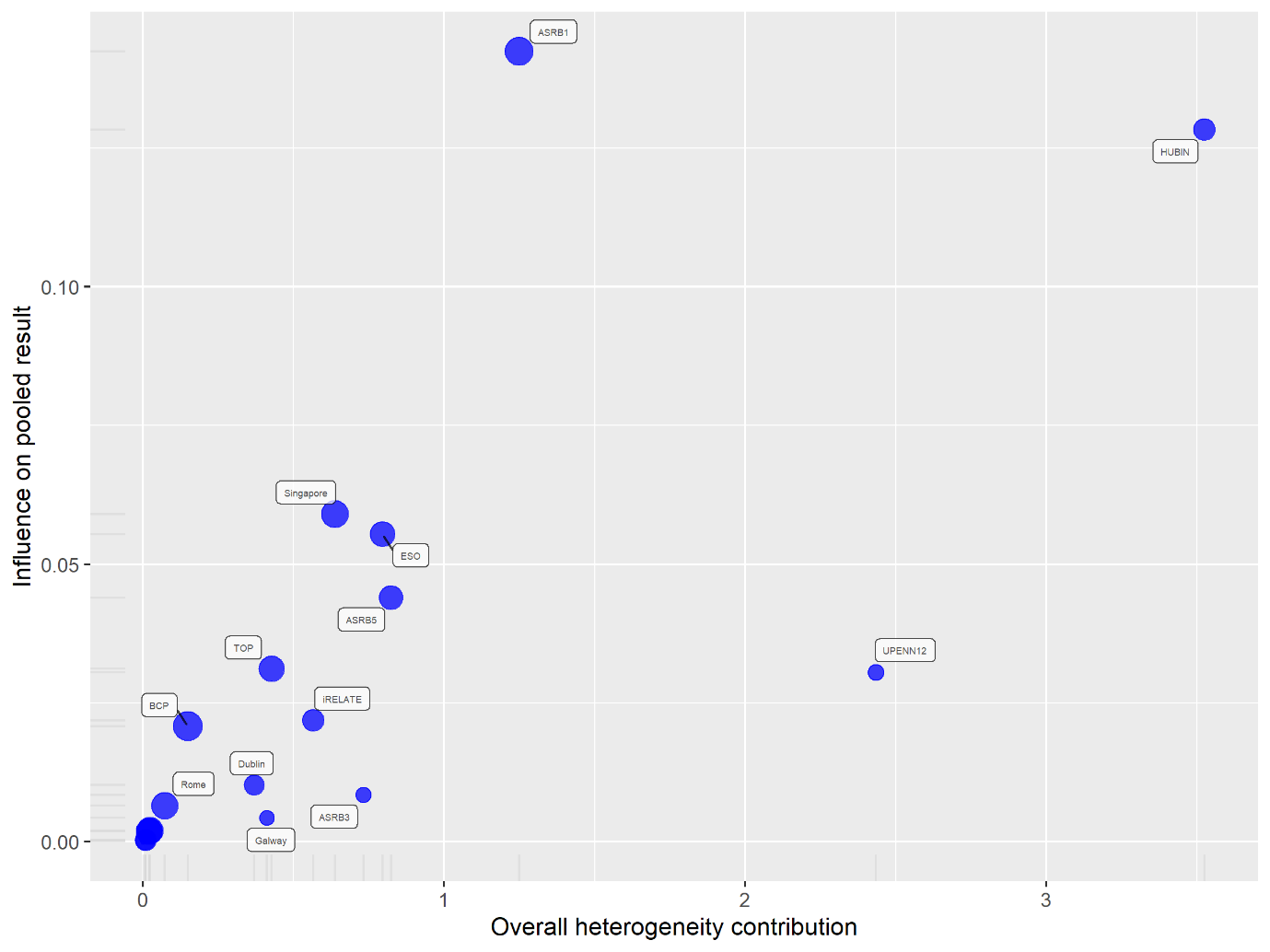
**

**Supplementary Figure 2.** Leave-One-Out analysis for the positive symptom and temporal-FA meta-analysis sorted by correlation. All leave-one-out estimates are close to the pooled estimate of *r* = -0.0802 within the range of [-0.09, -0.07], suggesting stability and consistency of the pooled estimate across studies.

**
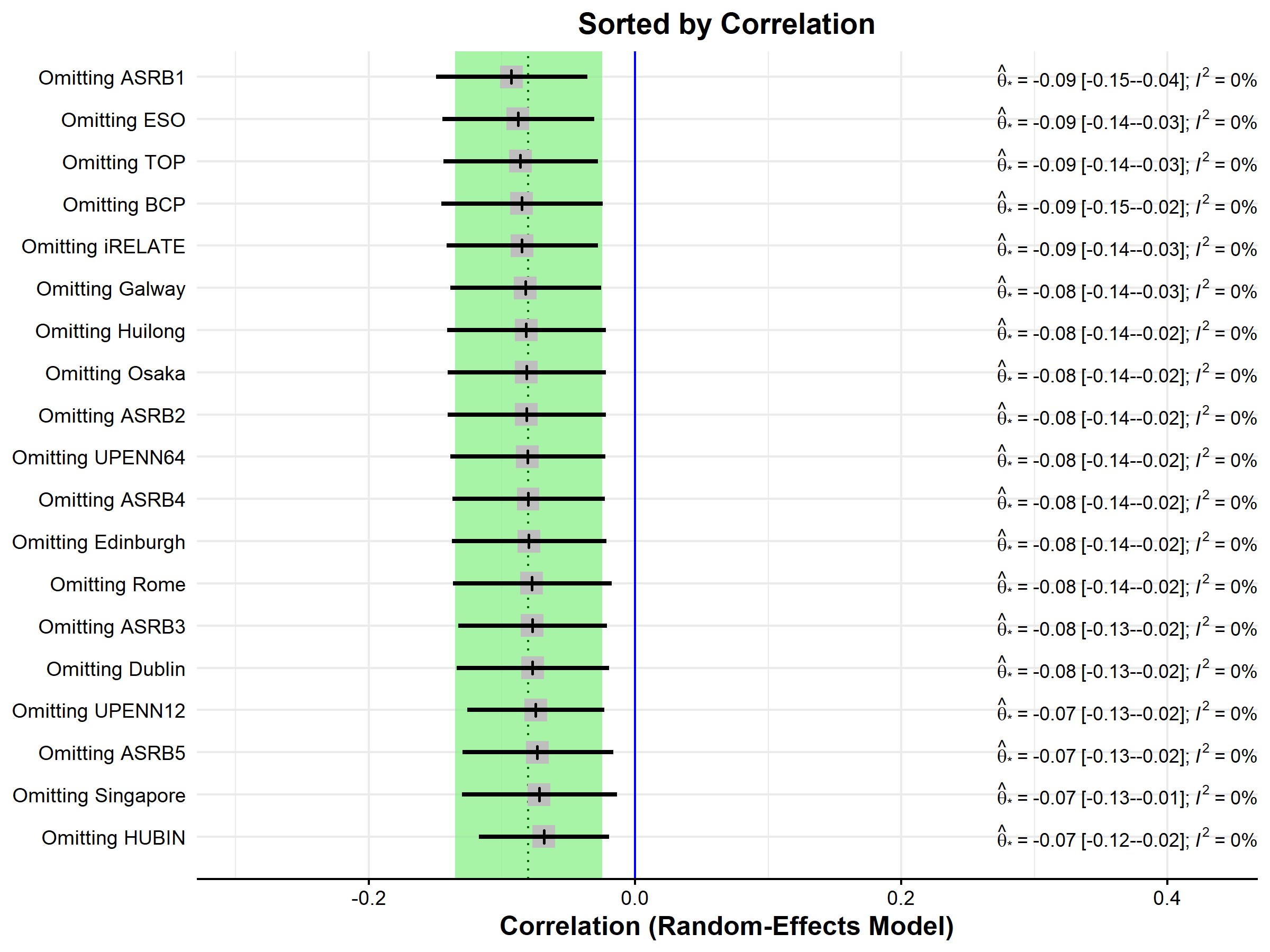
**

**Supplementary Figure 3.** Leave-One-Out analysis for the positive symptom and temporal-FA meta-analysis sorted by heterogeneity as measured by I^2^. All leave-one-out heterogeneity estimates are ~0%, indicating low heterogeneity across all sites.

**
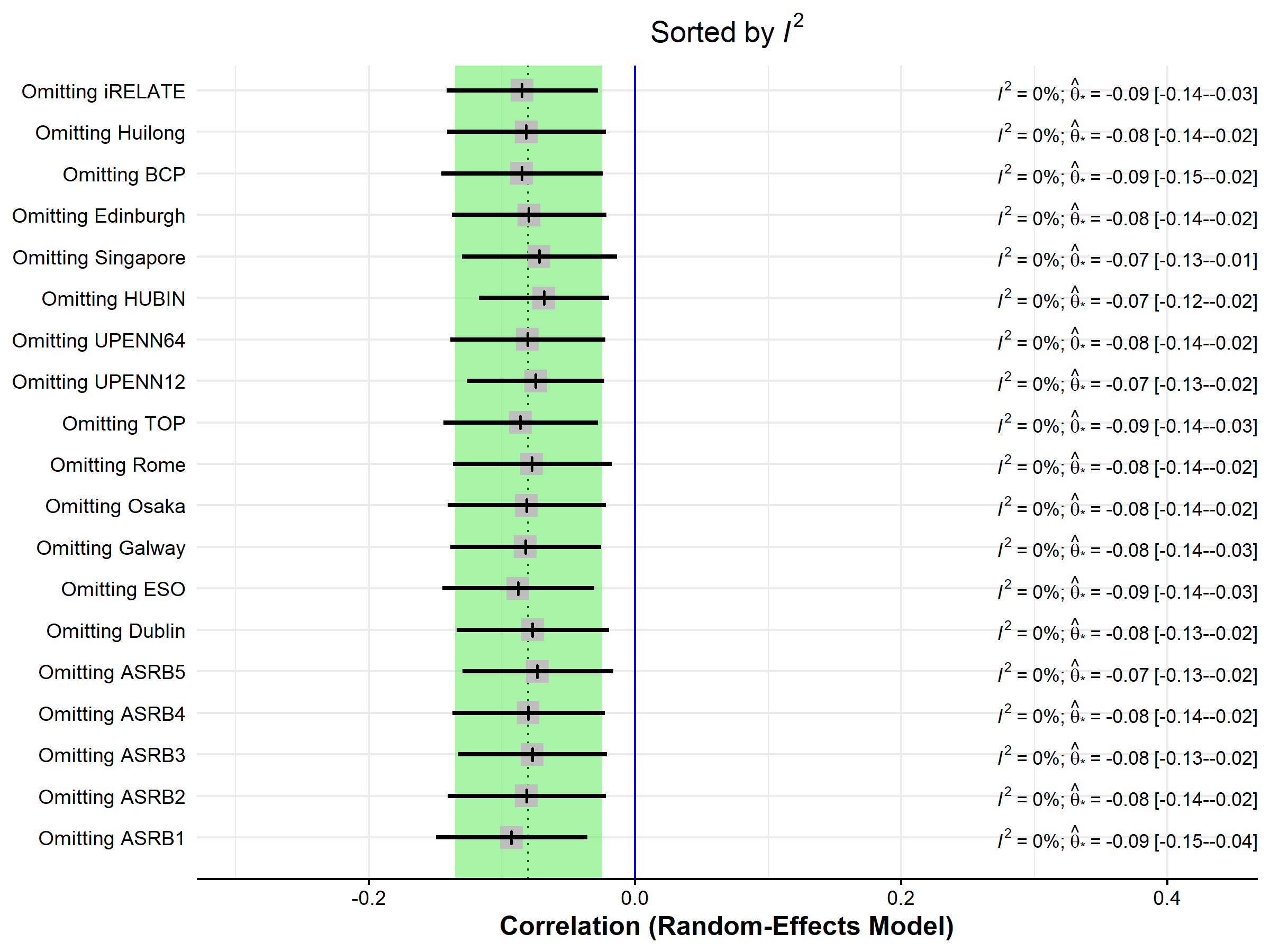
**

**Supplementary Table 6.** Summary of the meta-analysis results for positive symptoms and global-FA, showing a significant inverse association across all 19 sites (r = -0.0686 [-0.1303, -0.0063], *p* = 0.0327), with a non-significant amount of residual heterogeneity (*p* = 0.64). *Note.* *r*=site correlation value, LLCI=lower-level confidence interval, ULCI=upper-level confidence interval, %W=percentage weight.

| **Site** | ***n*** | ***r*** | **LLCI** | **ULCI** | ***p*** | **%W** |
| --- | --- | --- | --- | --- | --- | --- |
| ASRB1 | 102 | 0.002 | -0.1925 | 0.1964 | 0.984 | 10.2 |
| ASRB2 | 76 | -0.032 | -0.2556 | 0.1949 | 0.784 | 7.5 |
| ASRB3 | 14 | 0.077 | -0.4729 | 0.5837 | 0.798 | 1.1 |
| ASRB4 | 6 | 0.176 | -0.7415 | 0.8641 | 0.758 | 0.3 |
| ASRB5 | 52 | -0.228 | -0.4716 | 0.0479 | 0.104 | 5.1 |
| Dublin | 29 | -0.141 | -0.4826 | 0.2378 | 0.469 | 2.7 |
| ESO | 66 | 0.071 | -0.174 | 0.3077 | 0.572 | 6.5 |
| Galway | 13 | 0.237 | -0.3611 | 0.697 | 0.445 | 1 |
| Osaka | 76 | -0.138 | -0.3525 | 0.0903 | 0.235 | 7.5 |
| Rome | 83 | -0.11 | -0.3181 | 0.1083 | 0.323 | 8.3 |
| TOP | 69 | 0.009 | -0.2282 | 0.2452 | 0.942 | 6.8 |
| UPENN12 | 15 | -0.462 | -0.7878 | 0.0658 | 0.083 | 1.2 |
| UPENN64 | 34 | 0 | -0.3382 | 0.3382 | 1 | 3.2 |
| HUBIN | 37 | -0.44 | -0.6687 | -0.1353 | 0.006 | 3.5 |
| Singapore | 85 | -0.155 | -0.3564 | 0.0601 | 0.157 | 8.5 |
| Edinburgh | 26 | -0.089 | -0.4605 | 0.309 | 0.669 | 2.4 |
| BCP | 122 | 0.032 | -0.1466 | 0.2086 | 0.727 | 12.3 |
| Huilong | 81 | -0.063 | -0.2775 | 0.1575 | 0.577 | 8.1 |
| iRELATE | 39 | -0.032 | -0.344 | 0.2864 | 0.848 | 3.7 |
| **Summary** | 1025 | -0.0686 | -0.1303 | -0.0063 | 0.0327 | 100 |

**Supplementary Figure 4.** Baujat plot for the analysis between positive symptoms and global-FA, showing the relative influence of each site on the pooled association (y-axis) and the overall heterogeneity (x-axis) across sites.


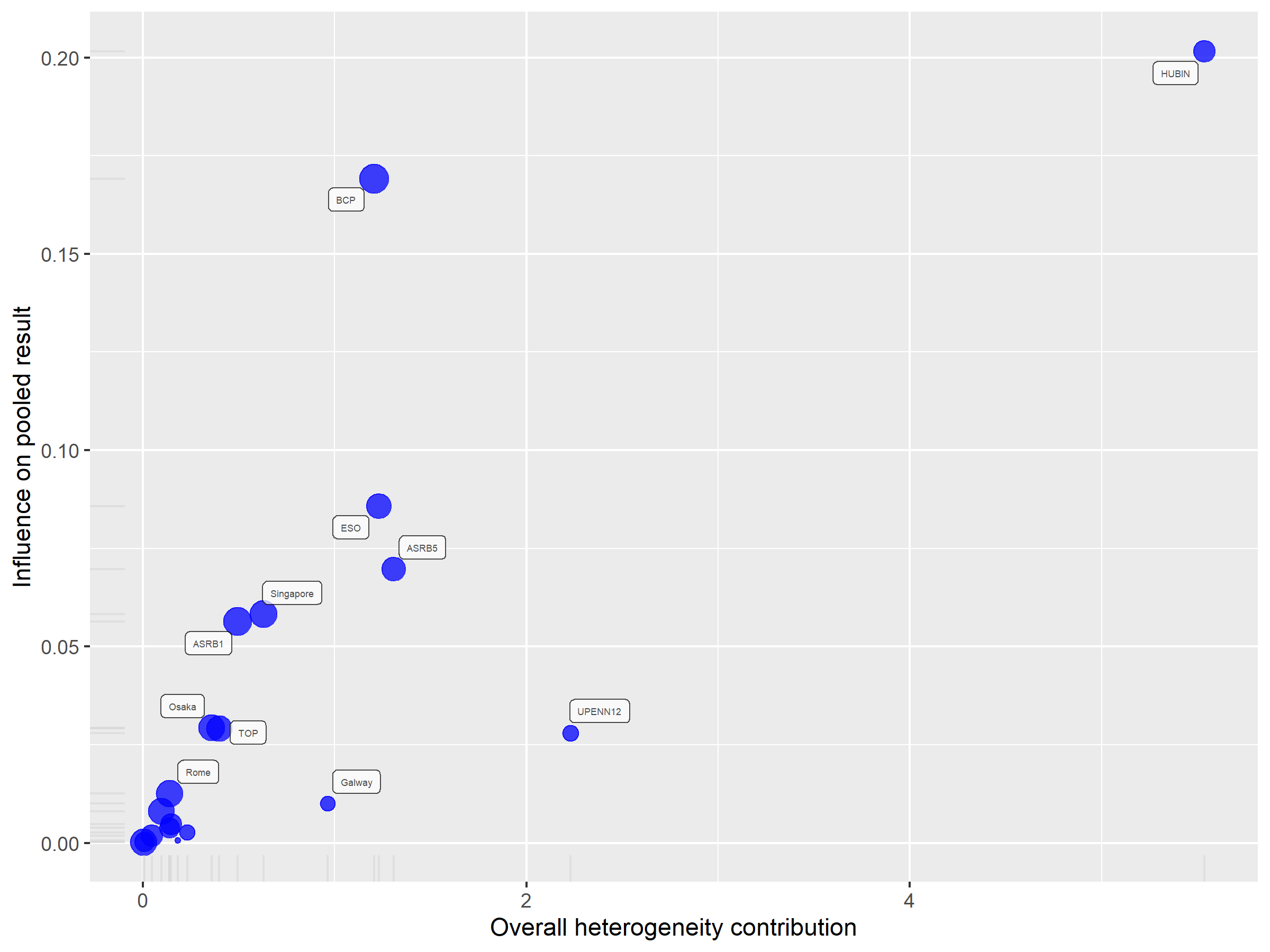


**Supplementary Figure 5.** Leave-One-Out analysis for the positive symptom and global-FA meta-analysis sorted by correlation. All leave-one-out estimates are close to the pooled estimate of *r* = -0.0686 within the range of [-0.08, -0.05], suggesting stability and consistency of the pooled estimate across studies.


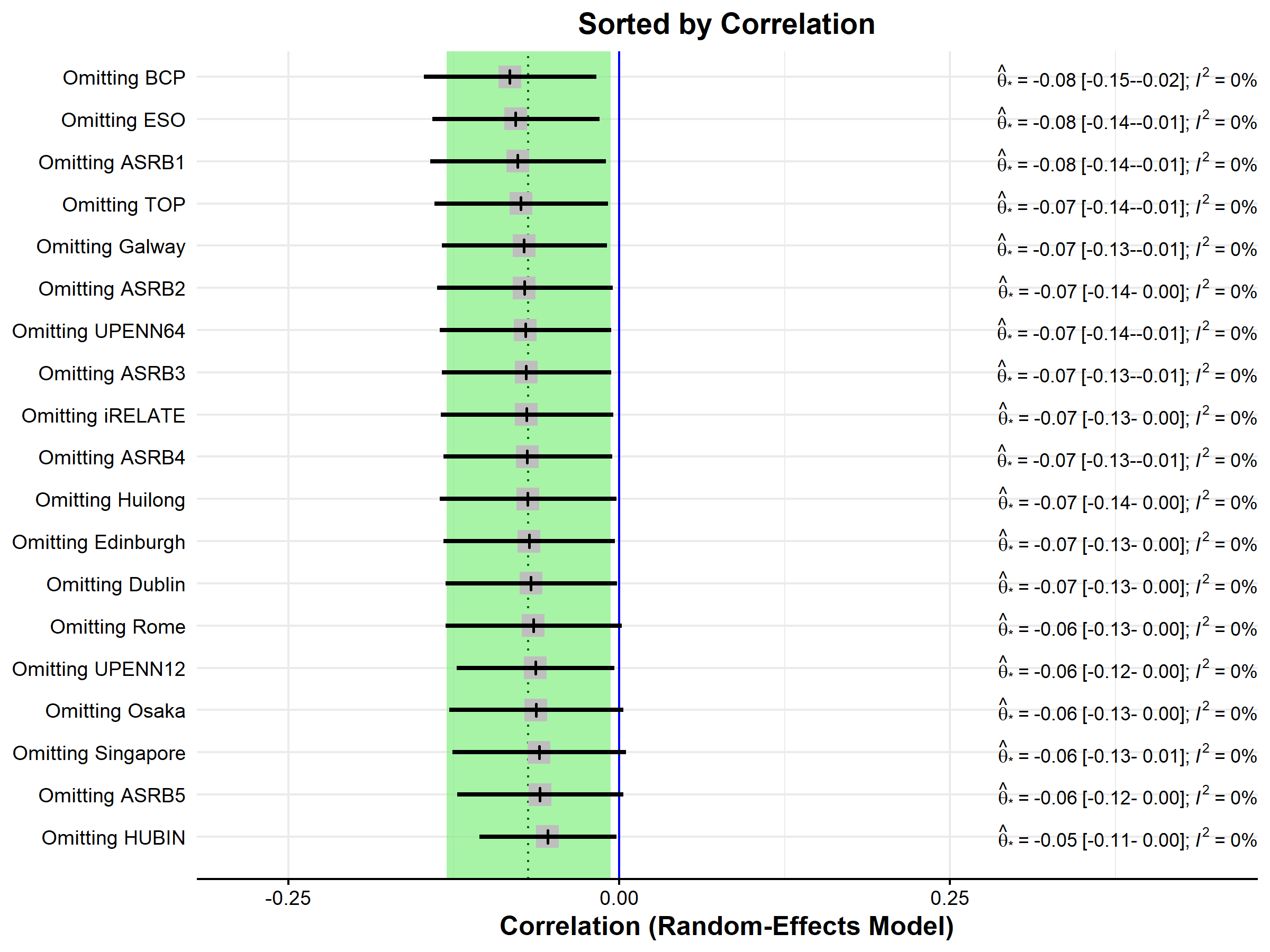


**Supplementary Figure 6.** Leave-One-Out analysis for the positive symptom and global-FA meta-analysis sorted by heterogeneity as measured by I^2^. All leave-one-out estimates for heterogeneity are ~0%, indicating low heterogeneity across sites.


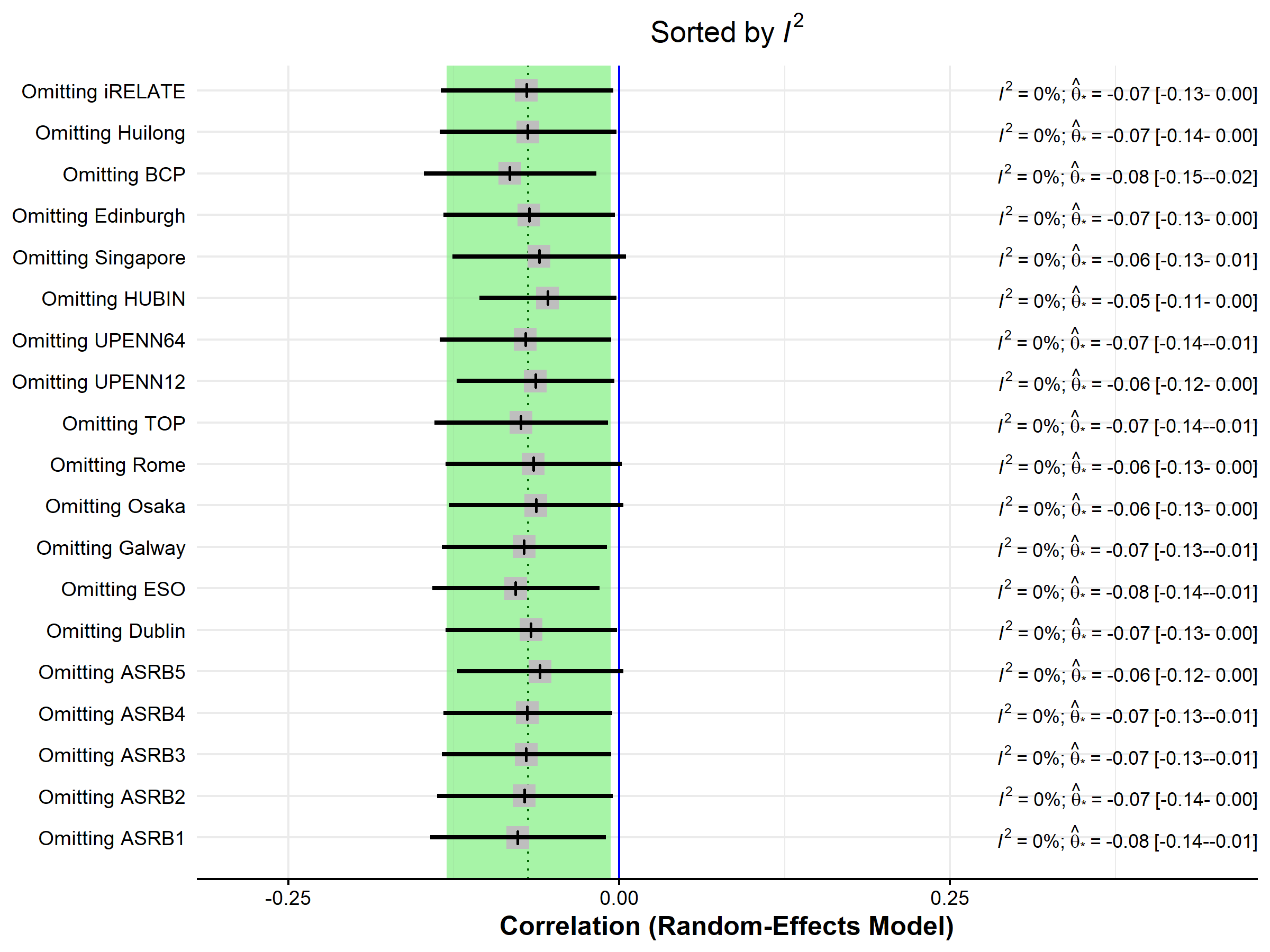


**Supplementary Table 7.** Summary of the meta-analysis results for positive symptoms and global-FA (excluding temporal brain regions), showing a significant inverse association across all 19 sites (*r* = -0.0635 [-0.1242, -0.0023], *p* = 0.0427), with a non-significant amount of residual heterogeneity (*p* = 0.67). *Note.* *r*=site correlation value, LLCI=lower-level confidence interval, ULCI=upper-level confidence interval, %W=percentage weight.

| **Site** | ***n*** | ***r*** | **LLCI** | **ULCI** | ***p*** | **%W** |
| --- | --- | --- | --- | --- | --- | --- |
| ASRB1 | 102 | 0.003 | -0.1916 | 0.1974 | 0.976 | 10.2 |
| ASRB2 | 76 | -0.063 | -0.2844 | 0.1648 | 0.59 | 7.5 |
| ASRB3 | 14 | -0.063 | -0.5744 | 0.4837 | 0.834 | 1.1 |
| ASRB4 | 6 | 0.341 | -0.6506 | 0.9027 | 0.538 | 0.3 |
| ASRB5 | 52 | -0.195 | -0.4443 | 0.0823 | 0.167 | 5.1 |
| Dublin | 29 | -0.055 | -0.4132 | 0.3179 | 0.779 | 2.7 |
| ESO | 66 | 0.152 | -0.0935 | 0.3801 | 0.224 | 6.5 |
| Galway | 13 | 0.148 | -0.4388 | 0.6463 | 0.637 | 1 |
| Osaka | 76 | -0.095 | -0.3137 | 0.1333 | 0.416 | 7.5 |
| Rome | 83 | -0.118 | -0.3254 | 0.1002 | 0.289 | 8.3 |
| TOP | 69 | 0.002 | -0.2348 | 0.2386 | 0.987 | 6.8 |
| UPENN12 | 15 | -0.444 | -0.7791 | 0.0884 | 0.098 | 1.2 |
| UPENN64 | 34 | -0.003 | -0.3408 | 0.3355 | 0.987 | 3.2 |
| HUBIN | 37 | -0.417 | -0.6528 | -0.1075 | 0.01 | 3.5 |
| Singapore | 85 | -0.148 | -0.3501 | 0.0672 | 0.177 | 8.5 |
| Edinburgh | 26 | -0.11 | -0.477 | 0.2897 | 0.596 | 2.4 |
| BCP | 122 | 0.032 | -0.1466 | 0.2086 | 0.727 | 12.3 |
| Huilong | 81 | -0.063 | -0.2775 | 0.1575 | 0.577 | 8.1 |
| iRELATE | 39 | -0.126 | -0.4246 | 0.1974 | 0.447 | 3.7 |
| **Summary** | 1025 | -0.0635 | -0.1242 | -0.0023 | 0.043 | 100 |

**Supplementary Figure 7.** Meta-analysis results for positive symptoms and global-FA, excluding temporal brain regions (*n* = 1,025), showing a significant inverse association across 19 ENIGMA sites (*r* = -0.0635 [-0.1242, -0.0246], *p* = 0.0427) with a non-significant degree of residual heterogeneity between sites (*p* = 0.67).


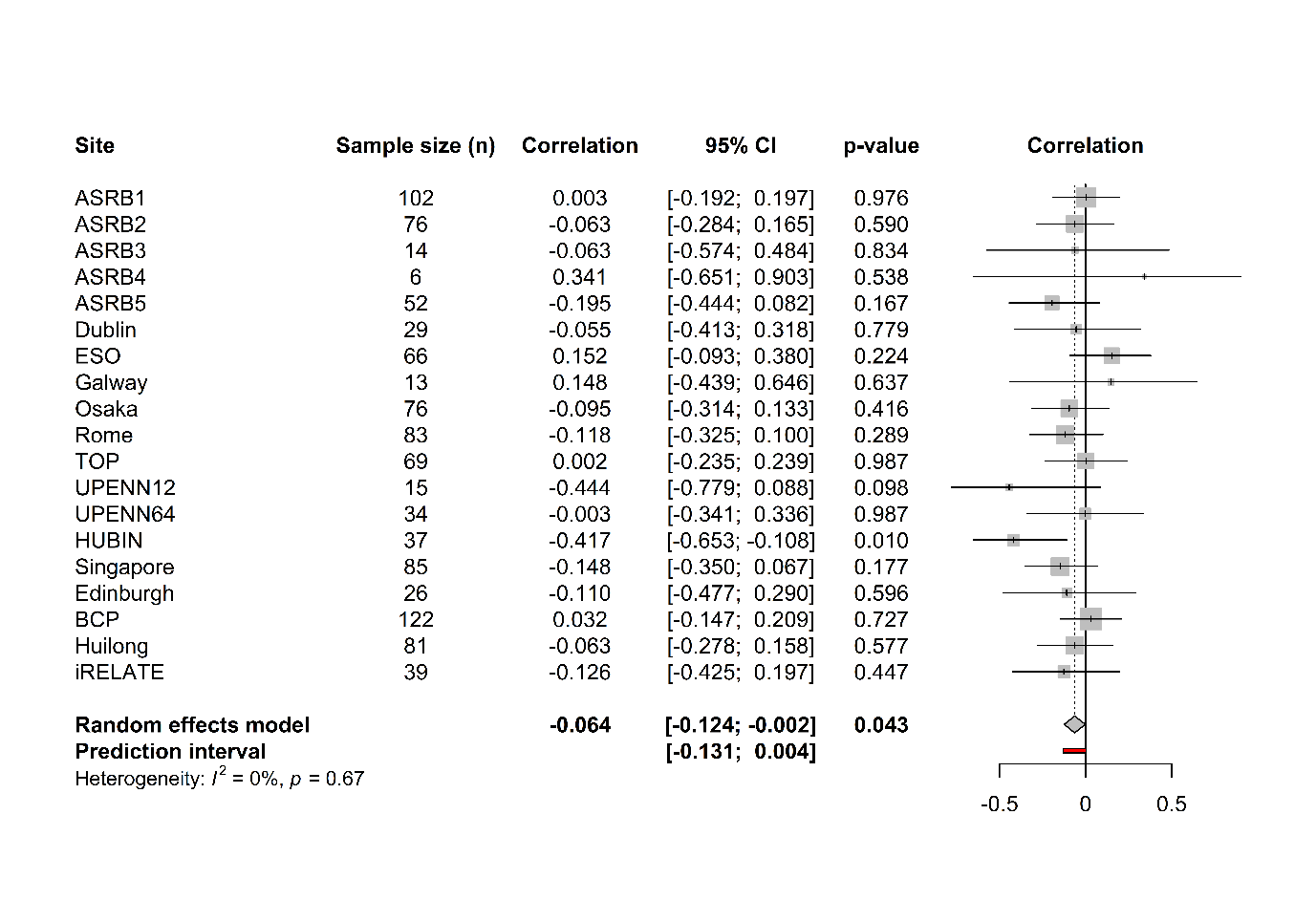


**Supplementary Figure 8.** Baujat plot for the analysis between positive symptoms and global-FA (excluding temporal regions) showing the relative influence of each site on the pooled association (y-axis) and the overall heterogeneity (x-axis) across sites.

**
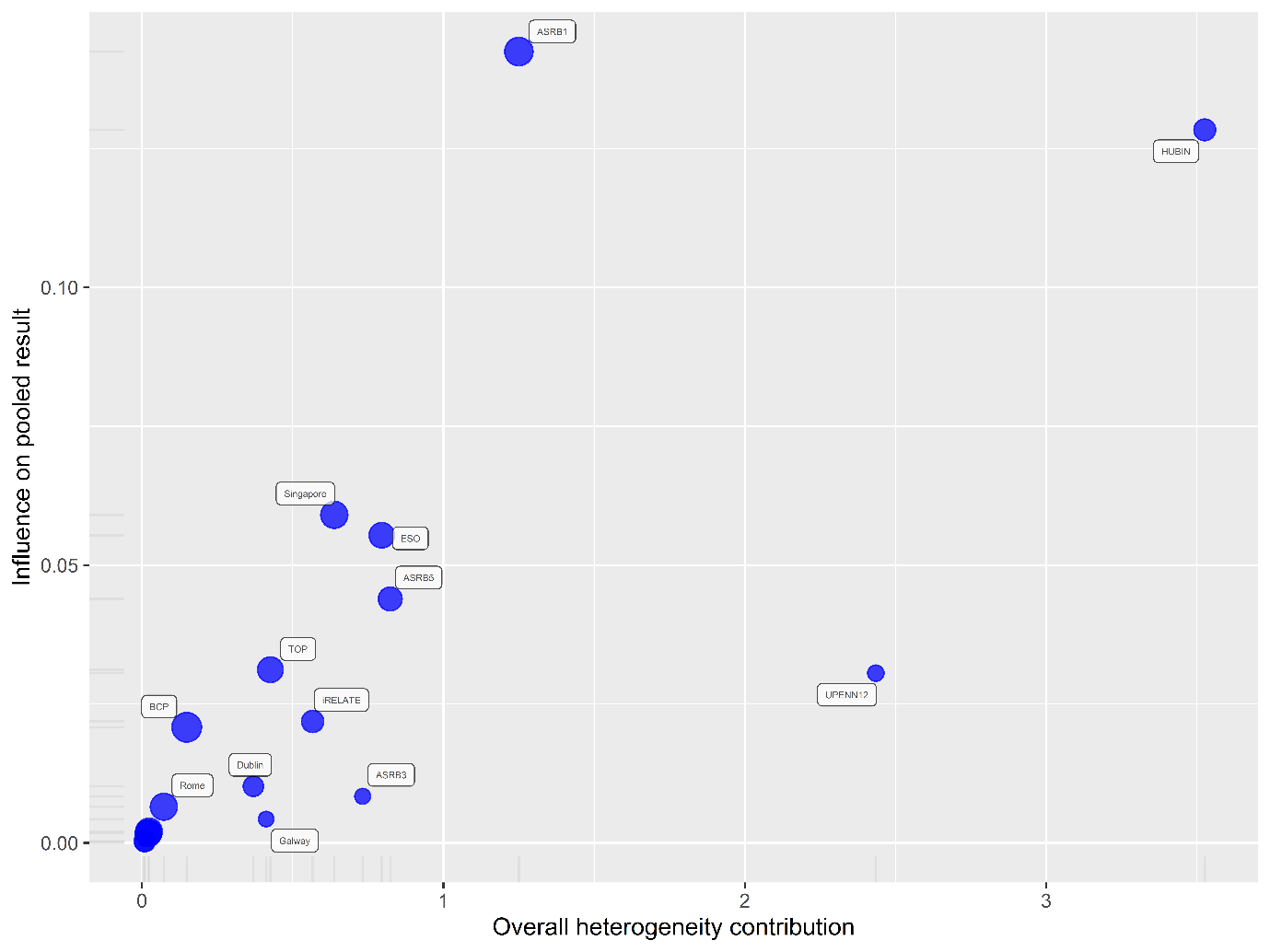
**

**Supplementary Figure 9.** Leave-One-Out analysis for the meta-analysis between positive symptoms and global-FA (excluding temporal regions) sorted by correlation. All leave-one-out estimates are close to the pooled estimate of *r* = -0.0635 within the range of [-0.08, -0.05], suggesting stability and consistency of the pooled estimate across studies.


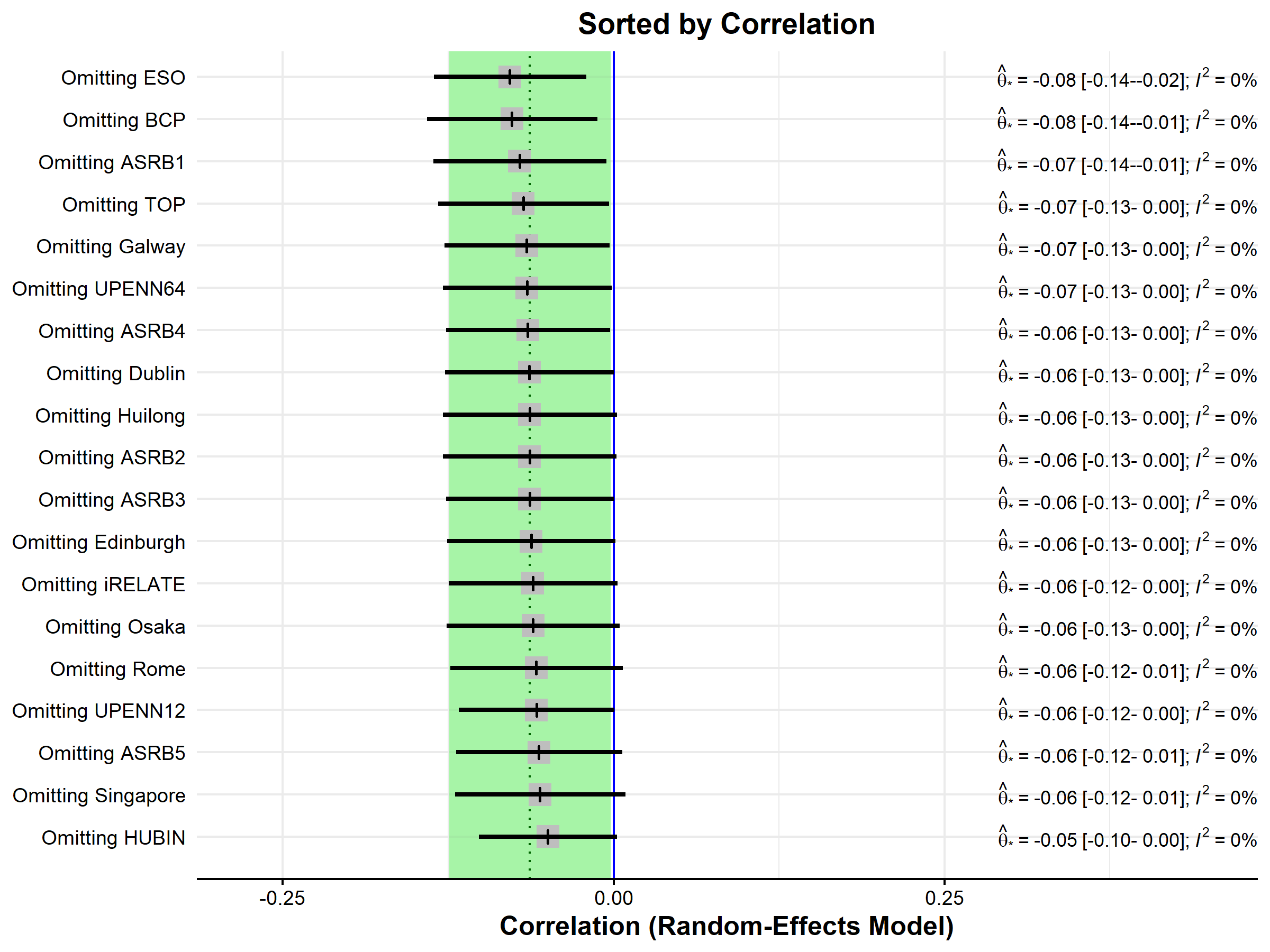


**Supplementary Figure 10.** Leave-One-Out analysis for the positive symptom and temporal-FA, excluding temporal regions, meta-analysis sorted by heterogeneity as measured by I^2^. All leave-one-out estimates for heterogeneity are ~0%, indicating low heterogeneity across sites.

**
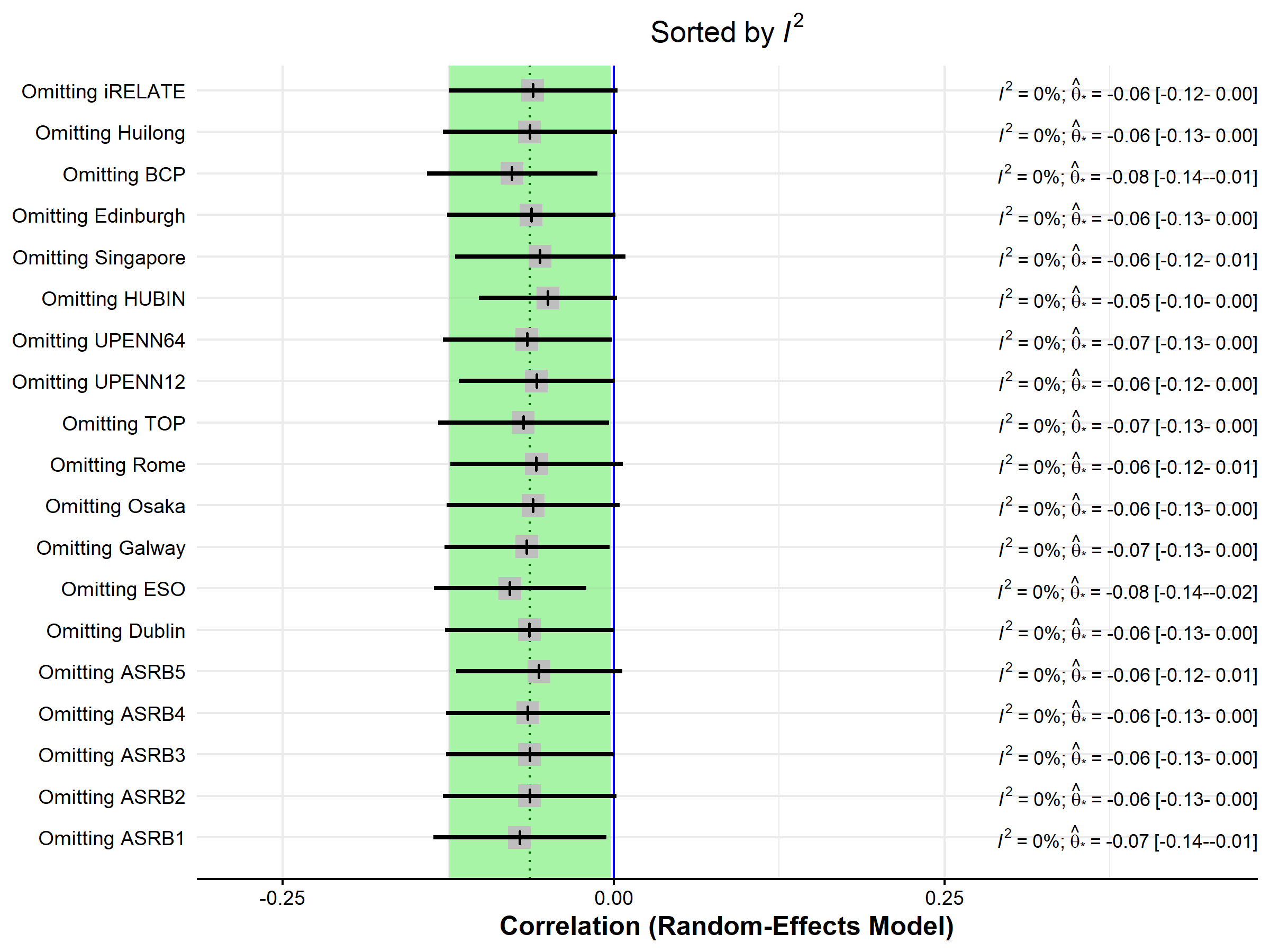
**

**Supplementary Table 8.** Summary of the meta-analysis results for negative symptoms and frontal-FA, showing a non-significant association across all 14 sites (*r* = -0.0861, [-0.1946, 0.0246], *p* = 0.117), with low to moderate heterogeneity between sites observed (I^2^ = 48%, *p* = 0.02).

*Note.* *r*=site correlation value, LLCI=lower-level confidence interval, ULCI=upper-level confidence interval, %W = percentage weight, CPZ = chlorpromazine equivalents (mg per day), mod1 = moderator 1, mod2 = moderator 2

| **Duration of illness** | **CPZ** | **Site** | ***n*** | ***r*** | **LLCI** | **ULCI** | ***p*** | **%W** |
| --- | --- | --- | --- | --- | --- | --- | --- | --- |
| 19.56 | 340 | Dublin | 29 | -0.324 | -0.617 | 0.048 | 0.087 | 5.2 |
| 0.91 | 311 | ESO | 66 | -0.071 | -0.308 | 0.174 | 0.574 | 8.6 |
| 7.7 | 324 | Galway | 13 | 0.032 | -0.529 | 0.573 | 0.920 | 2.5 |
| 11.46 | 750 | Osaka | 76 | -0.182 | -0.391 | 0.046 | 0.116 | 9.2 |
| 14 | 417 | Rome | 83 | -0.205 | -0.403 | 0.011 | 0.063 | 9.6 |
| 6.85 | 340 | TOP | 69 | 0.063 | -0.176 | 0.300 | 0.607 | 8.8 |
| 15.21 | 380 | UPENN12 | 15 | -0.380 | -0.747 | 0.165 | 0.166 | 2.9 |
| 12.75 | 1019 | UPENN64 | 34 | 0.055 | -0.289 | 0.386 | 0.760 | 5.8 |
| 28.38 | 415 | HUBIN | 37 | -0.239 | -0.522 | 0.093 | 0.156 | 6.2 |
| 5.98 | 192 | Singapore | 85 | 0.063 | -0.152 | 0.273 | 0.567 | 9.7 |
| 13.58 | 428 | Edinburgh | 26 | -0.272 | -0.597 | 0.129 | 0.181 | 4.8 |
| 16.36 | 473 | BCP | 122 | -0.276 | -0.432 | -0.103 | 0.002 | 11 |
| 1.08 | 600 | Huilong | 81 | 0.245 | 0.028 | 0.440 | 0.027 | 9.5 |
| 17.86 | 980 | iRELATE | 39 | 0.089 | -0.233 | 0.394 | 0.591 | 6.4 |
| **mod1** | **mod2** | **Summary** | 775 | -0.086 | -0.195 | 0.025 | 0.117 | 100 |

**Supplementary Figure 11.** The meta-analysis results for negative symptoms and frontal-FA (*n*=775) showing a non-significant pooled association across 14 ENIGMA sites (*r* = -0.0861, [-0.1946, 0.0246], *p* = 0.117) with low to moderate heterogeneity between sites (I^2^ = 48%, *p* = 0.02).


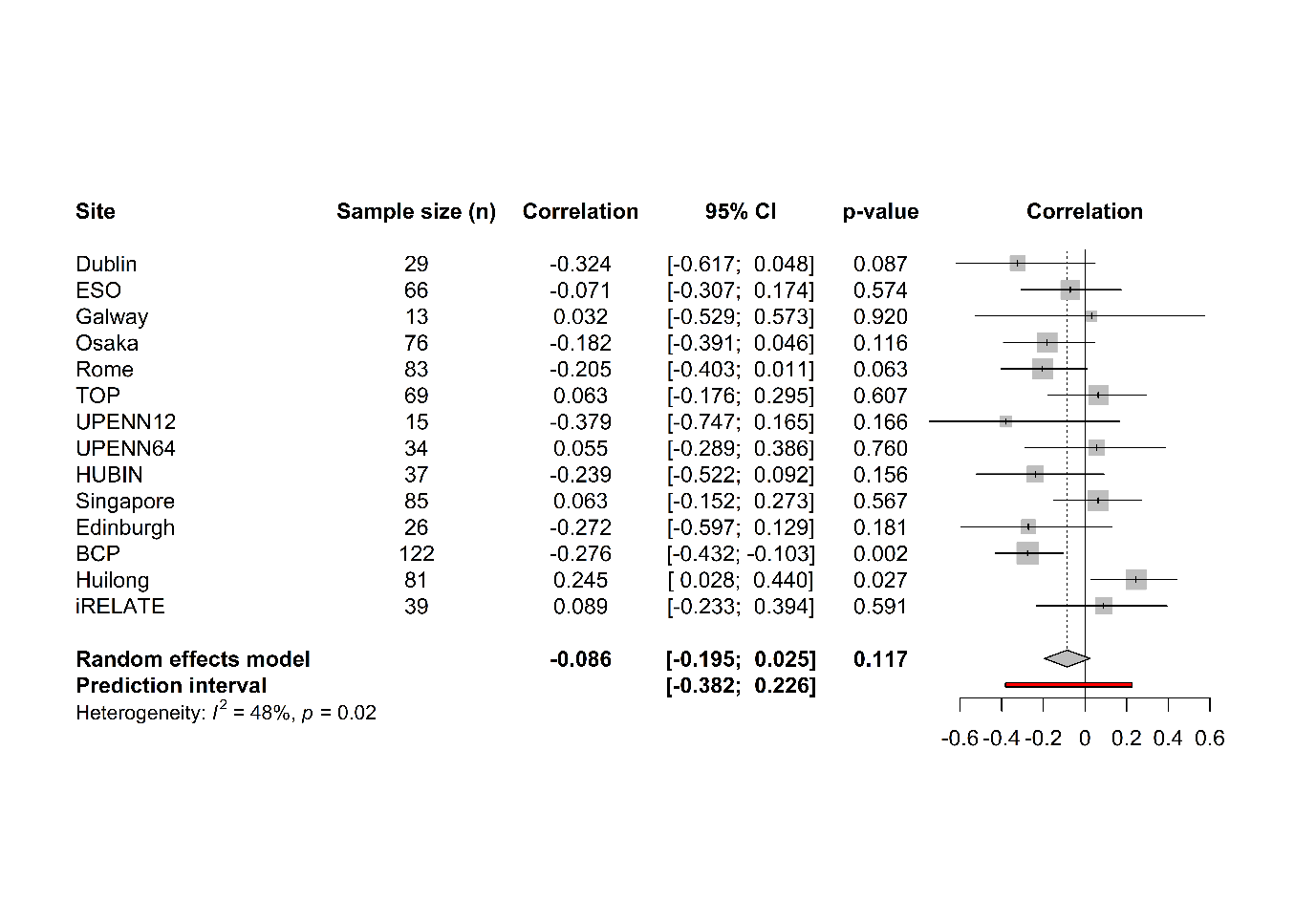


**Supplementary Table 9A**. Heterogeneity results for the mixed-effects model between negative symptoms and frontal-FA across sites with duration of illness as the moderator. Duration of illness accounted for the majority of residual heterogeneity in the model (I^2^ = 6.9%).

| **Mixed Effects Model** | |
| --- | --- |
| **Measure of variability** | **Value** |
| tau^2^ | 0.0015 (SE = 0.0088) |
| tau | 0.0384 |
| I^2^ | 6.90% |
| H^2^ | 1.07 |
| R^2^ | 91.84% |

**Supplementary Table 9B**. Moderator results for the mixed-effects model between negative symptoms and frontal-FA across sites with duration of illness as the moderator. Duration of illness was a significant moderator in the meta-regression model (*p* = 0.0065). *Note*. ** *p* < 0.01.

|  | **estimate** | **se** | **tval** | **df** | **pval** | **LLCI** | **ULCI** |
| --- | --- | --- | --- | --- | --- | --- | --- |
| **intercept** | 0.1149 | 0.0719 | 1.5971 | 12 | 0.1362 | -0.0418 | 0.2716 |
| **mod1 (duration)** | -0.018 | 0.0055 | -3.2829 | 12 | 0.0065** | -0.03 | -0.0061 |

**Supplementary Figure 12**. Baujat plot for the negative symptom and frontal-FA analysis showing the relative influence of each site on the pooled association (y-axis) and the overall heterogeneity (x-axis) across sites.


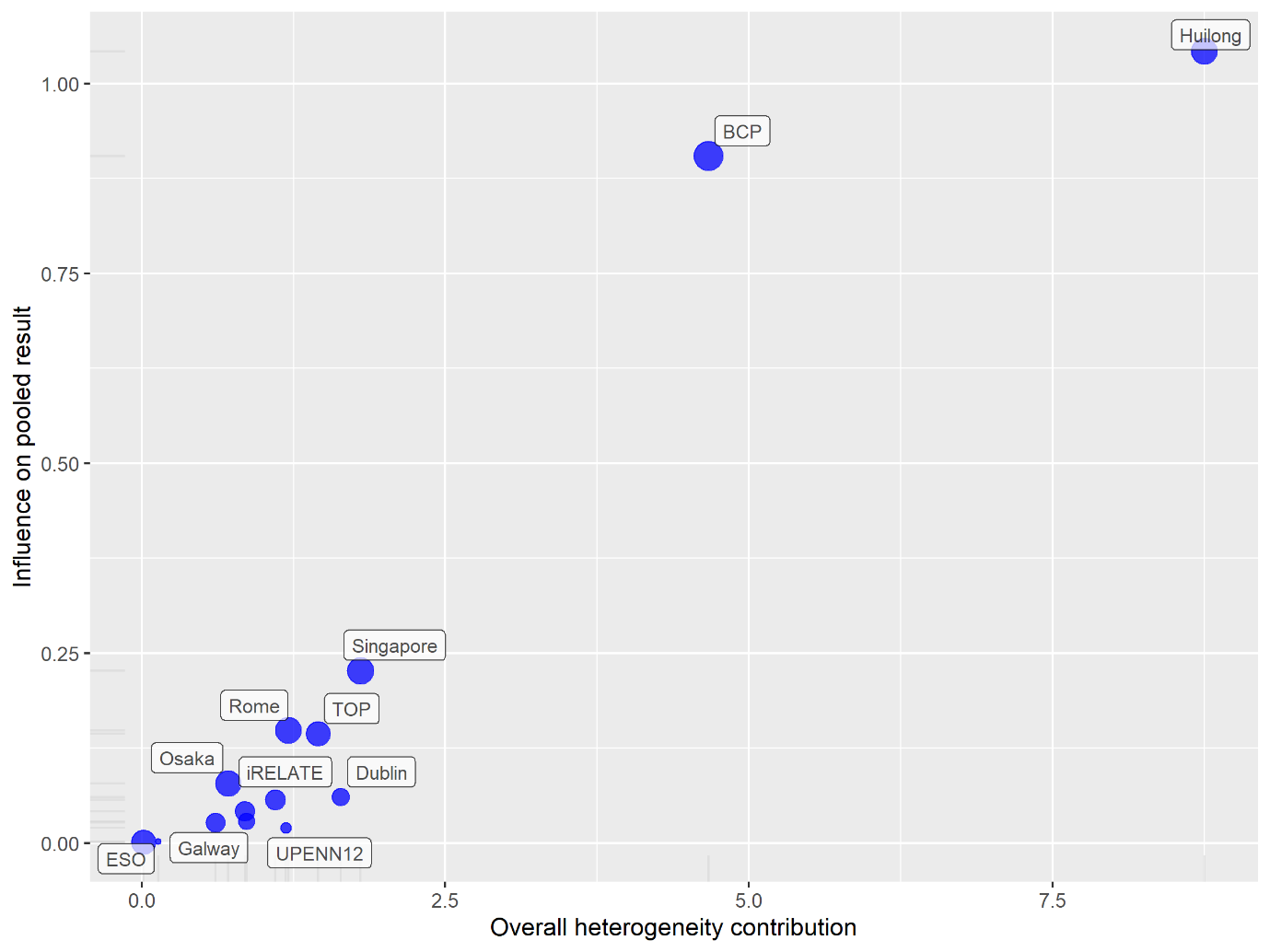


**Supplementary Figure 13.** Leave-One-Out analysis for the negative symptom and frontal-FA meta-analysis sorted by correlation. After omitting each of the 14 sites on a leave-one-out basis, the pooled correlation coefficient differs most when Huilong is omitted (estimated *r* = -0.12 [-0.22, -0.02], I^2^ = 21%).


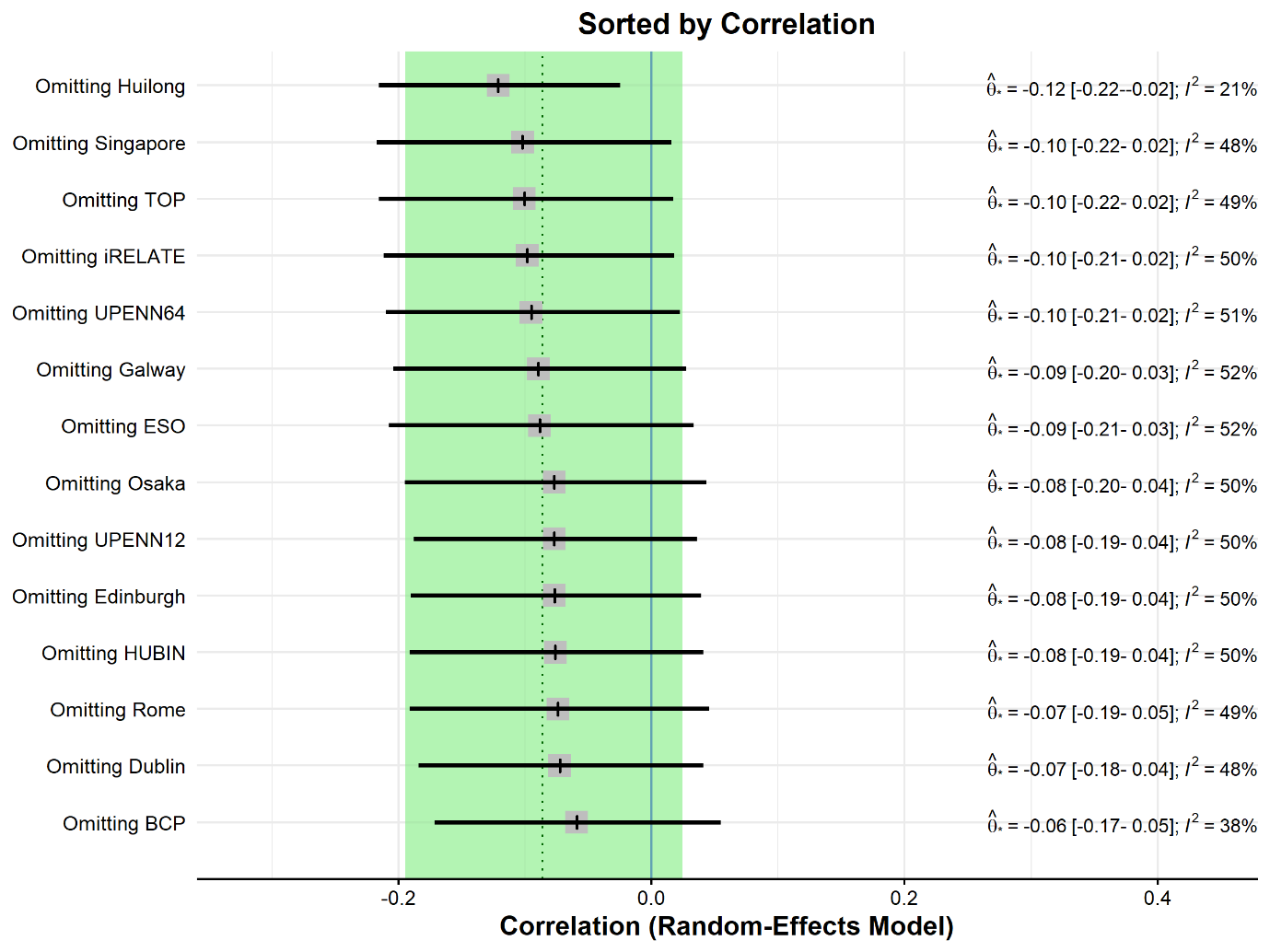


**Supplementary Figure 14.** Leave-One-Out analysis for the negative symptom and frontal-FA meta-analysis sorted by heterogeneity as measured by I^2^. After omitting each of the 14 sites on a leave-one-out basis, the heterogeneity between sites differs most when Huilong is omitted (estimated *r* = -0.12 [-0.22, -0.02], I^2^ = 21%).


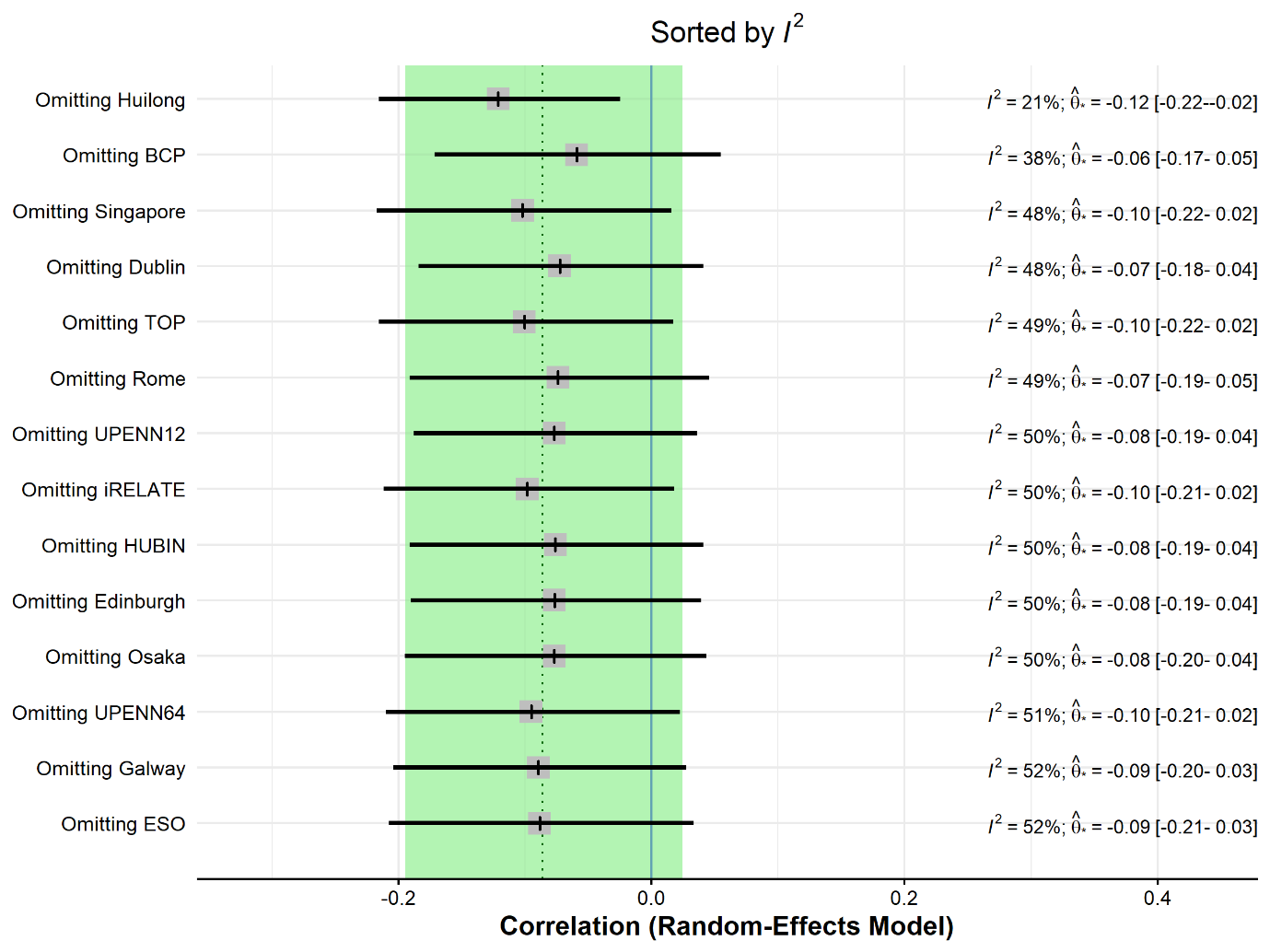


**Supplementary Figure 15.** Meta-analysis of negative symptoms and frontal-FA removing Huilong from the analysis. For the remaining sites (*n*=13), there was a significant negative pooled association between negative symptoms and frontal-FA (*r* = -0.121, [-0.215, -0.025], *p* = 0.018), with a non-significant degree of residual heterogeneity (I^2^ = 21%, *p* = 0.23).

**
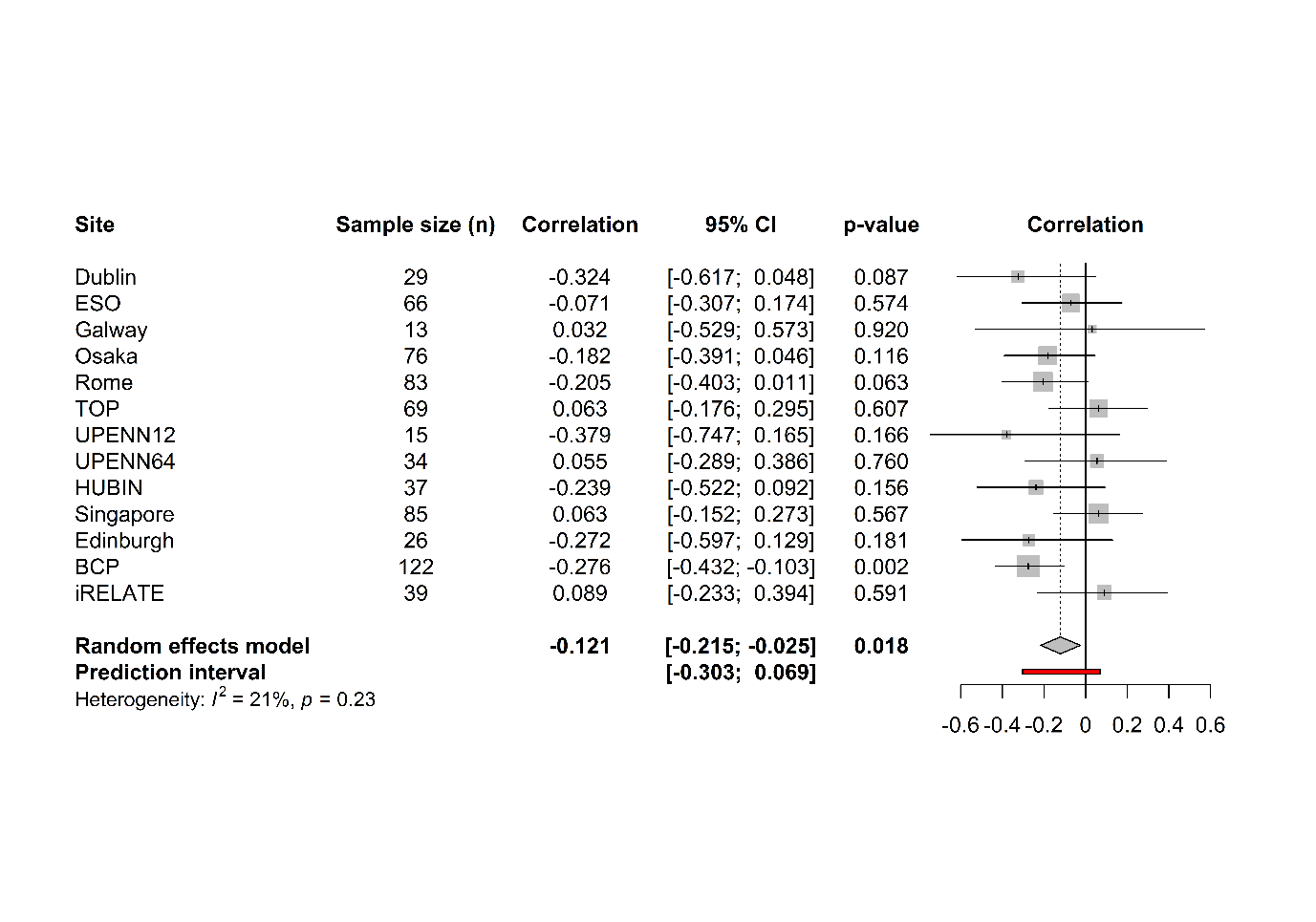
**

**Supplementary Table 10.** Summary of the meta-analysis results for negative symptoms and global-FA, showing a non-significant association between gFA and negative symptoms (*r* = -0.0084, [-0.1988, 0.0340], *p* = 0.148), and low-to-moderate heterogeneity between sites (I^2^ = 54%, *p* <0.01). *Note.* r=site correlation value, LLCI=lower-level confidence interval, ULCI=upper-level confidence interval, %W = percentage weight, CPZ = chlorpromazine equivalents (mg per day), mod1 = moderator 1, mod2 = moderator 2.

| **Duration of illness** | **CPZ** | **Site** | **n** | **r** | **LLCI** | **ULCI** | ***p*** | **%W** |
| --- | --- | --- | --- | --- | --- | --- | --- | --- |
| 19.56 | 340 | Dublin | 29 | -0.323 | -0.616 | 0.050 | 0.088 | 5.4 |
| 0.91 | 311 | ESO | 66 | -0.134 | -0.364 | 0.111 | 0.284 | 8.5 |
| 7.7 | 324 | Galway | 13 | 0.055 | -0.512 | 0.588 | 0.862 | 2.7 |
| 11.46 | 750 | Osaka | 76 | -0.138 | -0.352 | 0.090 | 0.236 | 9.1 |
| 14 | 417 | Rome | 83 | -0.279 | -0.467 | -0.068 | 0.010 | 9.4 |
| 6.85 | 340 | TOP | 69 | 0.045 | -0.194 | 0.278 | 0.716 | 8.7 |
| 15.21 | 380 | UPENN12 | 15 | -0.372 | -0.742 | 0.174 | 0.177 | 3.1 |
| 12.75 | 1019 | UPENN64 | 34 | 0.100 | -0.247 | 0.424 | 0.576 | 6 |
| 28.38 | 415 | HUBIN | 37 | -0.164 | -0.164 | 0.169 | 0.334 | 6.3 |
| 5.98 | 192 | Singapore | 85 | 0.063 | -0.152 | 0.273 | 0.567 | 9.4 |
| 13.58 | 428 | Edinburgh | 26 | -0.311 | -0.624 | 0.086 | 0.122 | 5 |
| 16.36 | 473 | BCP | 122 | -0.247 | -0.407 | -0.072 | 0.006 | 10.6 |
| 1.08 | 600 | Huilong | 81 | 0.295 | 0.082 | 0.482 | 0.007 | 9.3 |
| 17.86 | 980 | iRELATE | 39 | 0.078 | -0.244 | 0.384 | 0.641 | 6.5 |
| **mod1** | **mod2** | **Summary** | 775 | -0.084 | -0.199 | 0.034 | 0.148 | 100 |

**Supplementary Figure 16.** The meta-analysis results for negative symptoms and global-FA (*n*=775), showing a non-significant pooled association across 14 ENIGMA sites (*r* = -0.0084, [-0.1988, 0.0340], *p* = 0.148) with low to moderate heterogeneity between sites (I^2^ = 54%, *p* = <0.01).


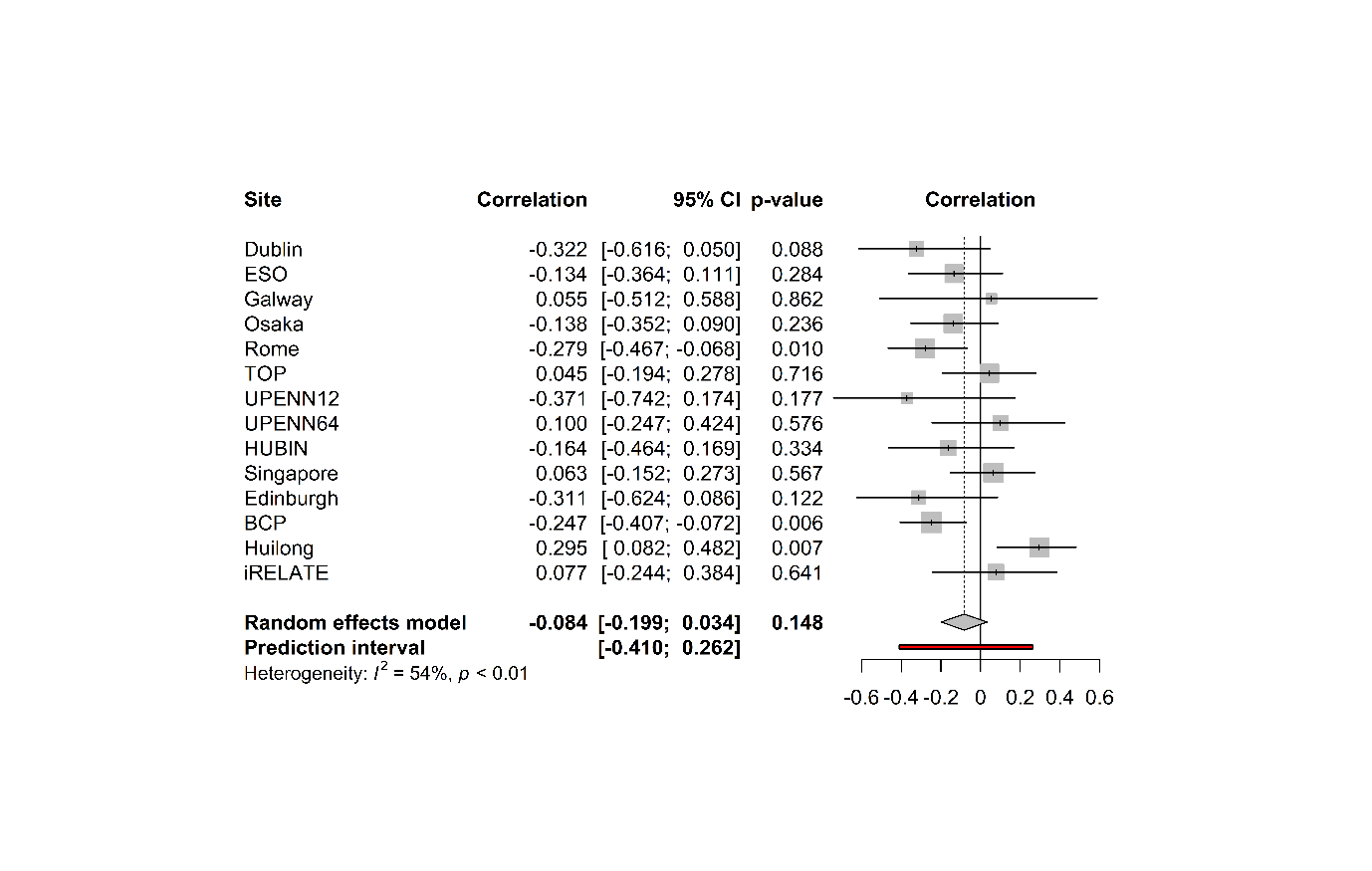


**Supplementary Table 11A.** Heterogeneity results for the mixed-effects model between negative symptoms and global-FA across sites with duration of illness as the moderator. Duration of illness accounted for a significant amount of residual heterogeneity in the model (I^2^ = 32.45%).

| **Mixed Effects Model** | |
| --- | --- |
| **Measure of variability** | **Value** |
| tau^2^ | 0.0096 (SE =0.0123) |
| tau | 0.0979 |
| I^2^ | 32.45% |
| H^2^ | 1.48 |
| R^2^ | 58.04% |

**Supplementary Table 11B.** Moderator results for the mixed-effects model between negative symptoms and global-FA across sites with duration of illness as the moderator. Duration of illness was a significant moderator in the meta-regression model (*p* = 0.0065). *Note*. **p* < 0.05.

|  | **estimate** | **se** | **tval** | **df** | **pval** | **LLCI** | **ULCI** |
| --- | --- | --- | --- | --- | --- | --- | --- |
| **intercept** | 0.0935 | 0.0868 | 1.0778 | 12 | 0.3023 | -0.0955 | 0.2826 |
| **mod1 (duration)** | -0.0155 | 0.0065 | -2.3859 | 12 | 0.0344* | -0.0296 | -0.0013 |

**Supplementary Figure 17.** Baujat plot for the negative symptom and global-FA analysis showing the influence of each site on the pooled association (y-axis) and the overall heterogeneity (x-axis) across sites.


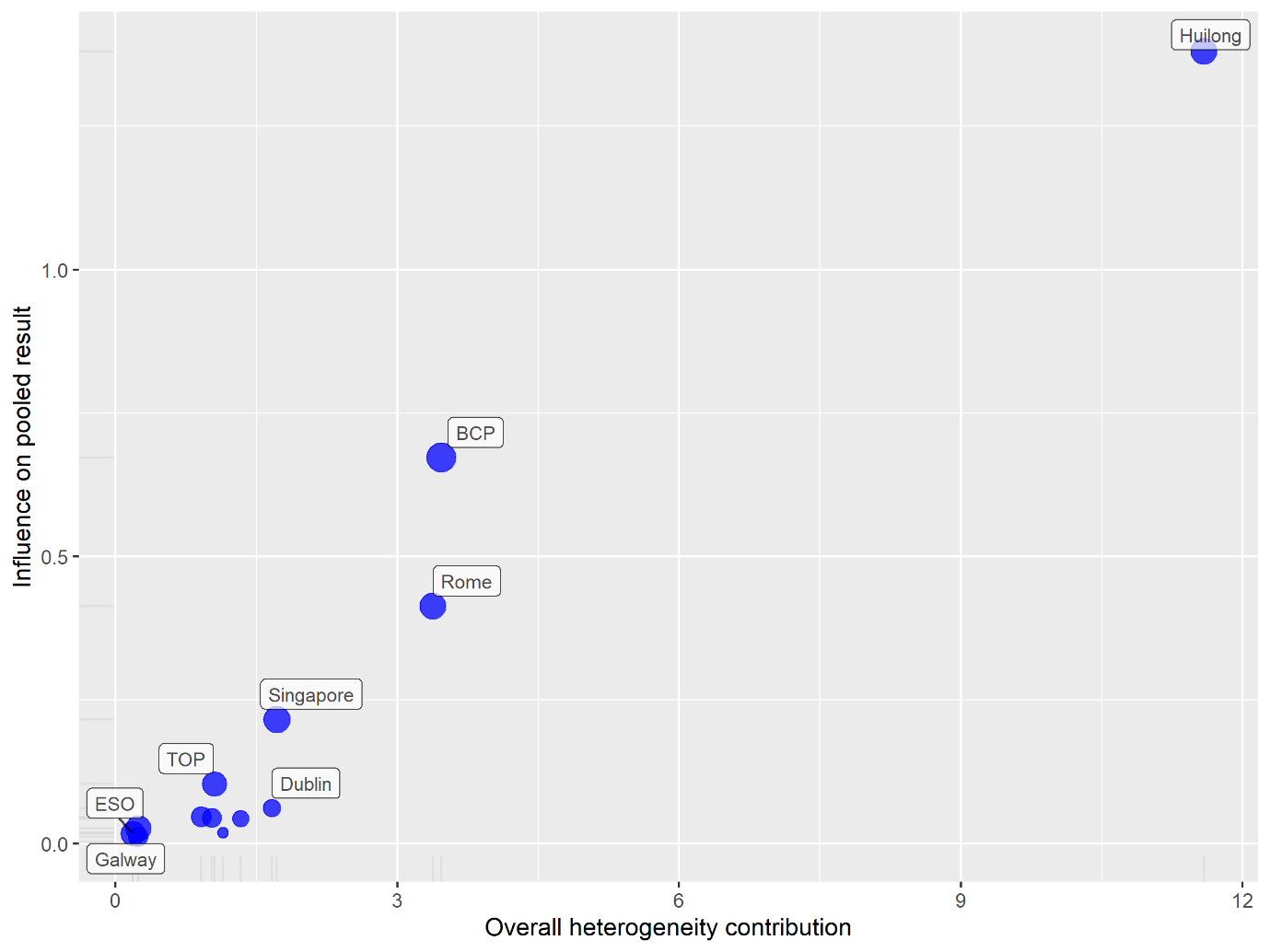


**Supplementary Figure 18.** Leave-One-Out analysis for the negative symptom and global-FA analysis sorted by correlation. After omitting each of the 14 sites on a leave-one-out basis, the pooled correlation coefficient differs most when Huilong is omitted (estimated *r* = -0.12 [-0.22, -0.03], I^2^ = 21%).


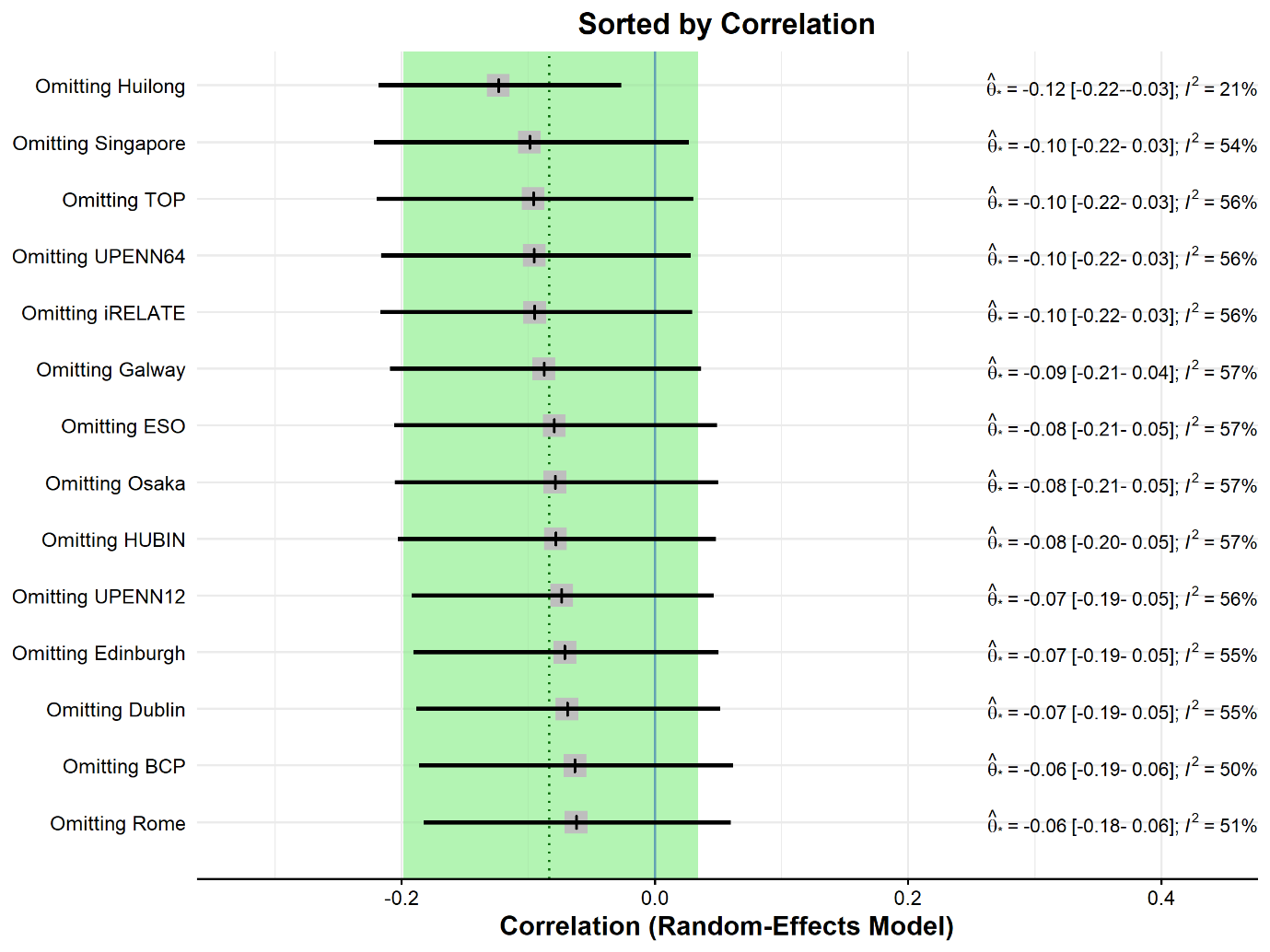


**Supplementary Figure 19.** Leave-One-Out analysis for the positive symptom and global-FA analysis sorted by heterogeneity as measured by I^2^. After omitting each of the 14 sites on a leave-one-out basis, the heterogeneity between sites differs most when Huilong is omitted (estimated *r* = -0.12 [-0.22, -0.03], I^2^ = 21%).


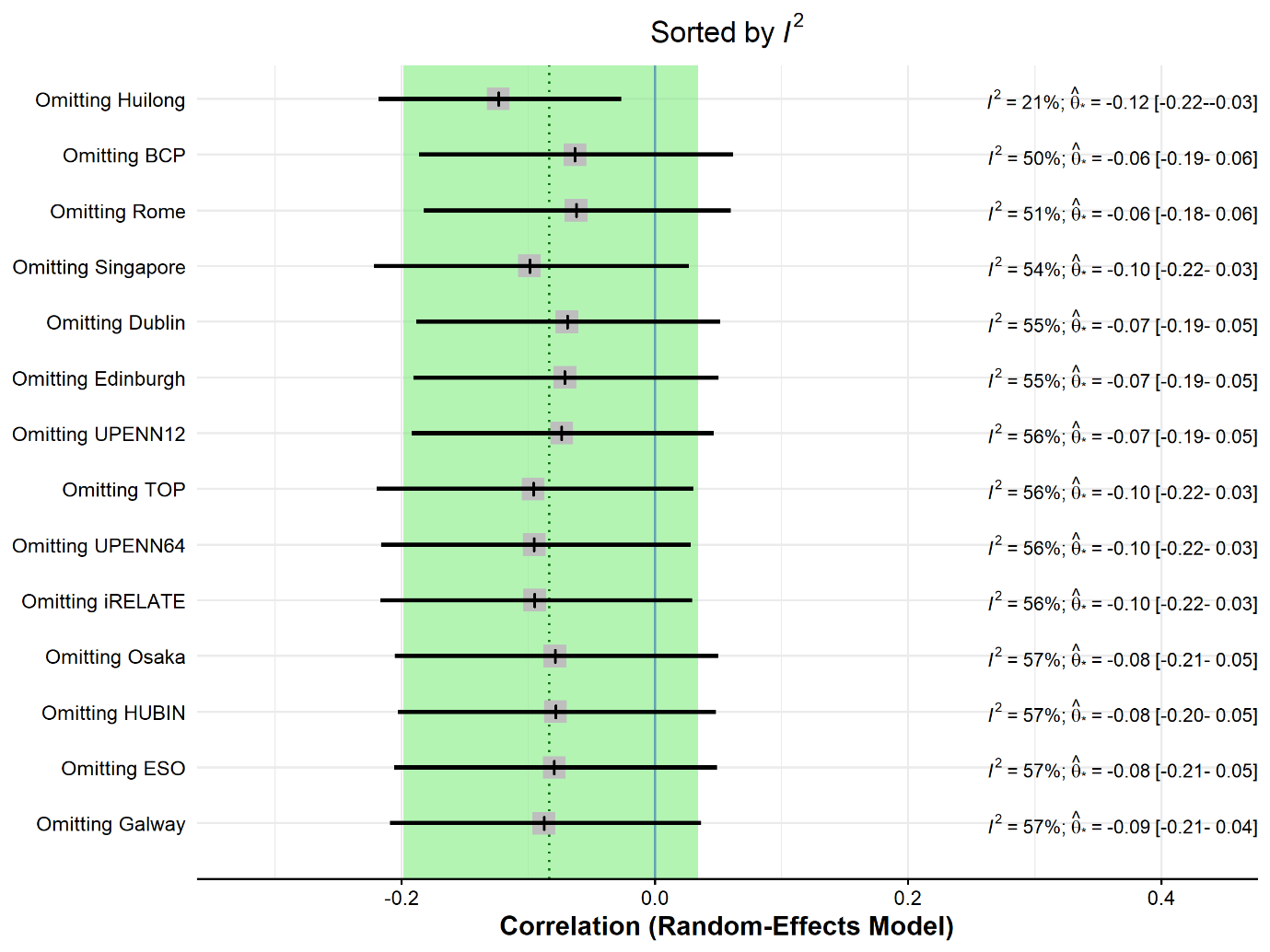


**Supplementary Figure 20.** Meta-analysis of negative symptoms and global-FA removing Huilong from the analysis. Across the remaining sites (*n*=13), there was a significant negative pooled association (*r* = -0.124, [-0.218, -0.027], *p* = 0.017), with a non-significant degree of residual heterogeneity (I^2^ = 21%, *p* = 0.23).


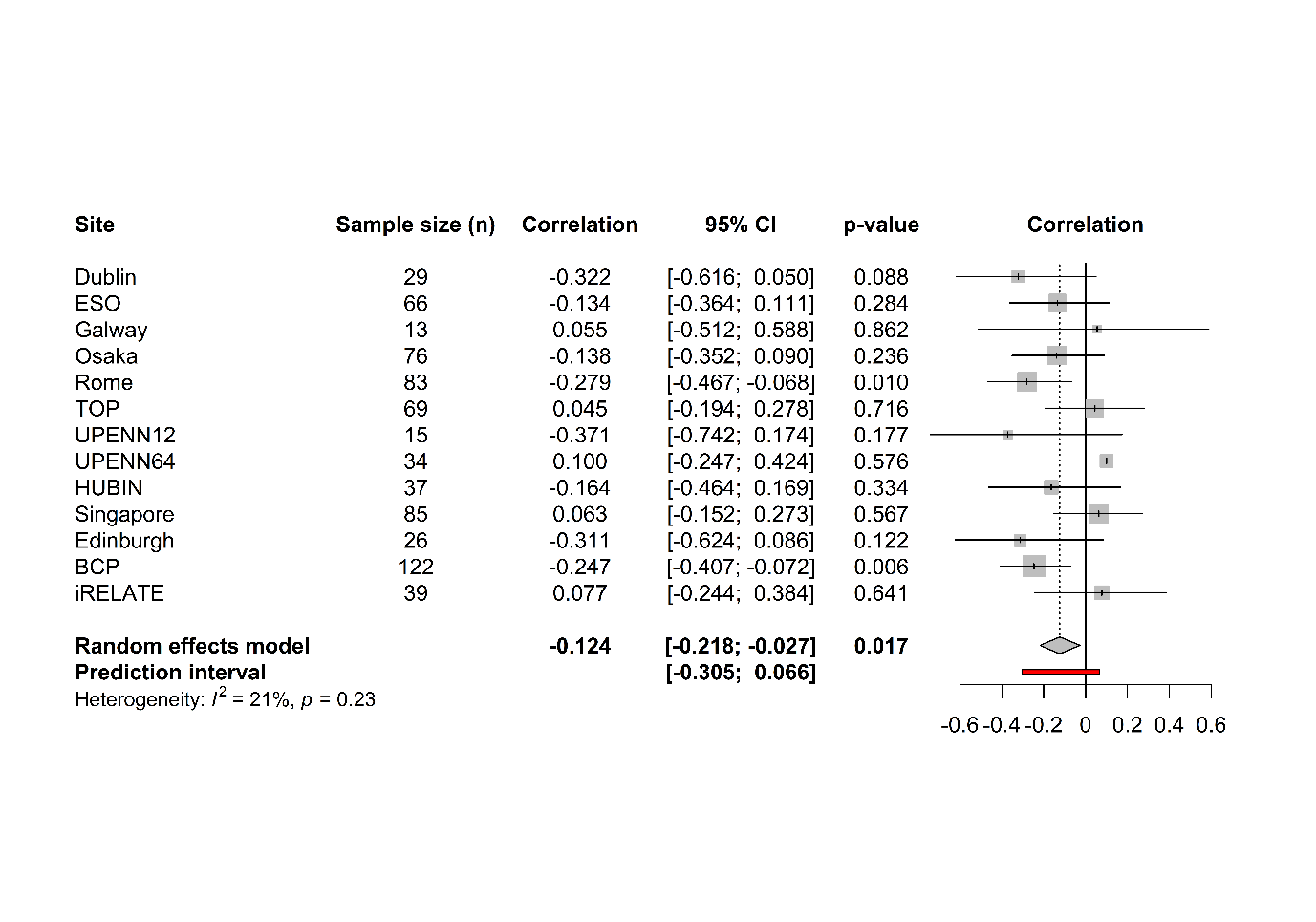


**Supplementary Table 12.** Summary of the meta-analysis results for negative symptoms and global-FA, excluding frontal brain regions, showing a non-significant association across sites (*r* = -0.0521, [-0.1594; 0.0565], *p* = 0.3192), with low to moderate heterogeneity between sites observed (I^2^ = 45%, *p* = 0.03). *Note.* r=site correlation value, LLCI=lower-level confidence interval, ULCI=upper-level confidence interval, %W = percentage weight, CPZ = chlorpromazine equivalents (mg per day), mod1 = moderator 1, mod2 = moderator 2

| **Duration of illness** | **CPZ** | **Site** | **n** | **r** | **LLCI** | **ULCI** | **p** | **%W** |
| --- | --- | --- | --- | --- | --- | --- | --- | --- |
| 19.56 | 340 | Dublin | 29 | -0.283 | -0.588 | 0.093 | 0.138 | 5.1 |
| 0.91 | 311 | ESO | 66 | -0.170 | -0.396 | 0.075 | 0.172 | 8.6 |
| 7.7 | 324 | Galway | 13 | 0.055 | -0.512 | 0.588 | 0.862 | 2.4 |
| 11.46 | 750 | Osaka | 76 | -0.055 | -0.277 | 0.173 | 0.639 | 9.3 |
| 14 | 417 | Rome | 83 | -0.205 | -0.403 | 0.011 | 0.063 | 9.7 |
| 6.85 | 340 | TOP | 69 | 0.002 | -0.235 | 0.239 | 0.987 | 8.8 |
| 15.21 | 380 | UPENN12 | 15 | -0.295 | -0.701 | 0.256 | 0.292 | 2.8 |
| 12.75 | 1019 | UPENN64 | 34 | 0.141 | -0.207 | 0.458 | 0.428 | 5.7 |
| 28.38 | 415 | HUBIN | 37 | -0.095 | -0.406 | 0.236 | 0.579 | 6.1 |
| 5.98 | 192 | Singapore | 85 | 0.078 | -0.138 | 0.286 | 0.482 | 9.8 |
| 13.58 | 428 | Edinburgh | 26 | -0.303 | -0.618 | 0.095 | 0.133 | 4.7 |
| 16.36 | 473 | BCP | 122 | -0.190 | -0.355 | -0.012 | 0.036 | 11.3 |
| 1.08 | 600 | Huilong | 81 | 0.318 | 0.107 | 0.501 | 0.004 | 9.5 |
| 17.86 | 980 | iRELATE | 39 | 0.071 | -0.250 | 0.378 | 0.671 | 6.3 |
| **mod1** | **mod2** | **Summary** | 775 | -0.052 | -0.159 | 0.057 | 0.319 | 100 |

**Supplementary Figure 21.** The meta-analysis results for negative symptoms and global-FA, excluding frontal regions (*n*=775), showing a non-significant pooled association across 14 ENIGMA sites (*r* = -0.0521, [-0.1594; 0.0565], *p* = 0.3192), with low-to-moderate heterogeneity between sites (I^2^ = 45%, *p* = 0.03).


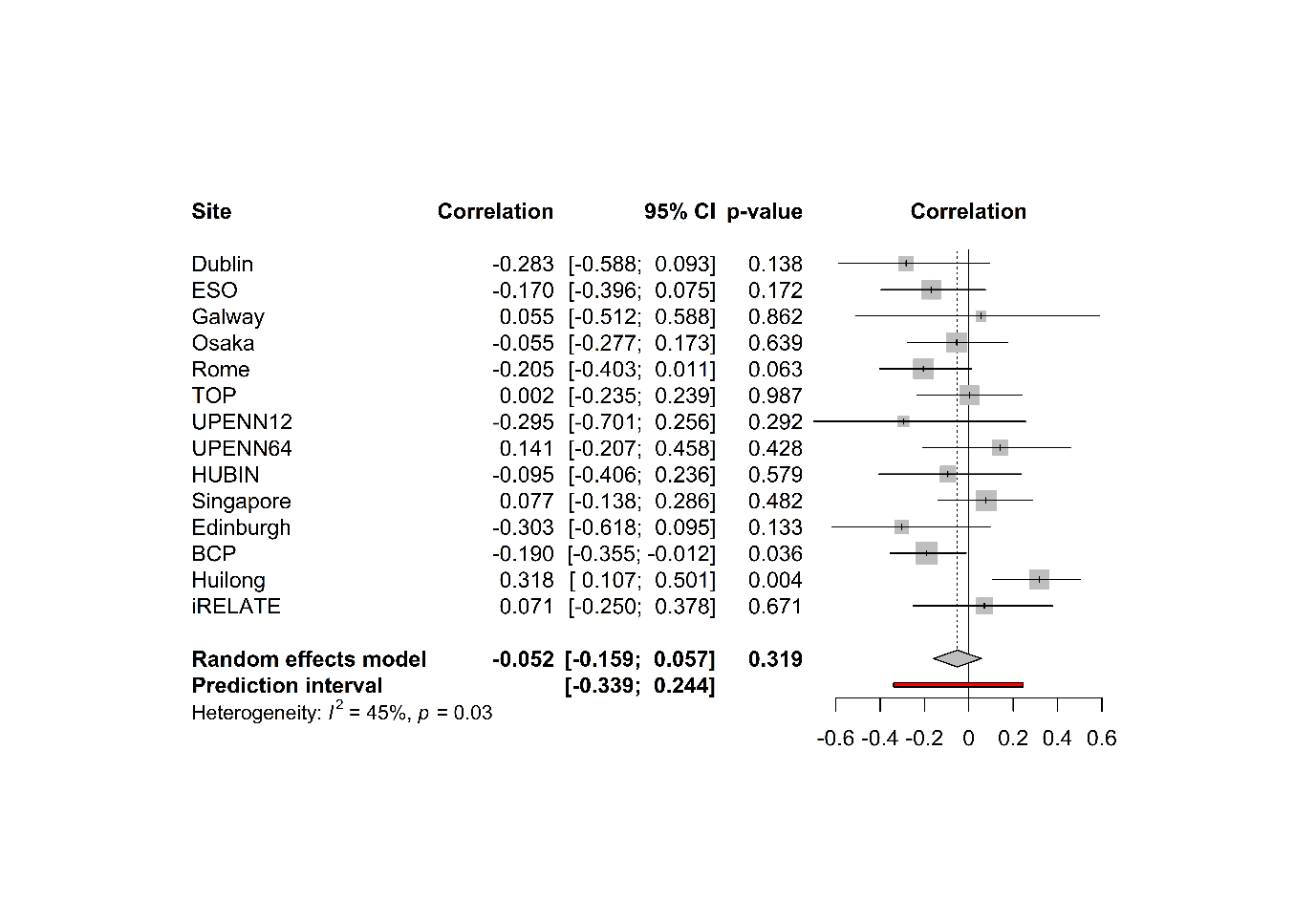


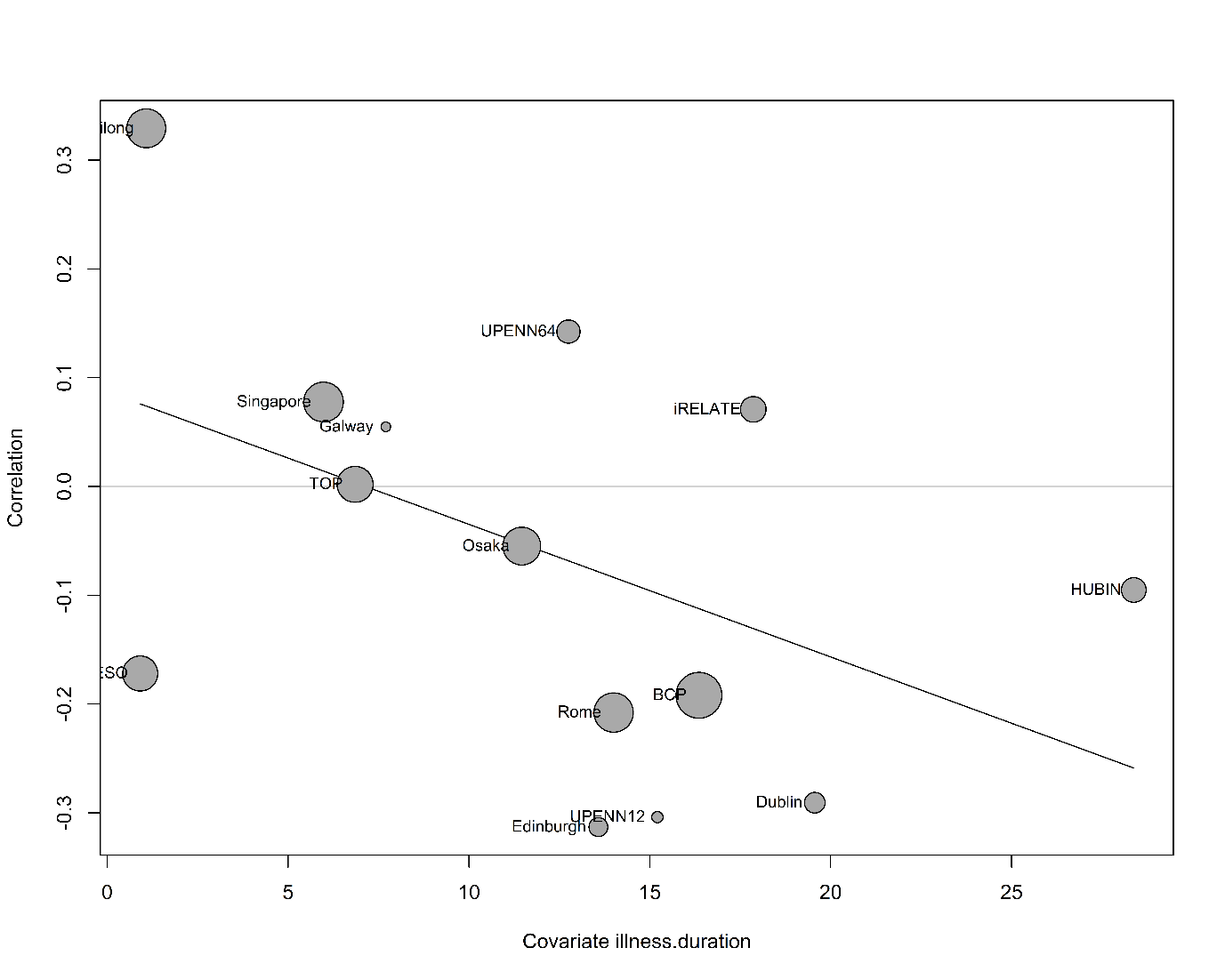
**Supplementary Figure 22.** The relationship between duration of illness and the size of the correlation coefficient between gFA (excluding frontal regions) and negative symptoms at each site (*n* = 14) included in the meta-regression model. Duration of illness shows a trend towards explaining a significant degree of residual heterogeneity (*p* = 0.0799, I^2^ = 30.6%).

**Supplementary Table 13A.** Heterogeneity results for the mixed-effects model between negative symptoms and global-FA, excluding frontal regions, across sites with duration of illness as the moderator. Duration of illness showed a trend towards explaining the residual heterogeneity across sites (I^2^ = 30.6%).

| **Mixed Effects Model** | |
| --- | --- |
| **Measure of variability** | **Value** |
| tau^2^ | 0.0088 (SE = 0.0119) |
| tau | 0.0937 |
| I^2^ | 30.56% |
| H^2^ | 1.44 |
| R^2^ | 46.09% |

**Supplementary Table 13B.** Moderator results for the mixed-effects model between negative symptoms and global-FA, excluding frontal brain regions, across sites with duration of illness as the moderator. Duration of illness showed a trend for explaining a significant degree of residual heterogeneity in the meta-regression model (*p* = 0.0799).

|  | **estimate** | **se** | **tval** | **df** | **pval** | **LLCI** | **ULCI** |
| --- | --- | --- | --- | --- | --- | --- | --- |
| **intercept** | 0.087 | 0.0851 | 1.0218 | 12 | 0.327 | -0.0985 | 0.2724 |
| **mod1 (duration)** | -0.0122 | 0.0064 | -1.9127 | 12 | 0.0799 | -0.0261 | 0.0017 |

**Supplementary Figure 23.** Baujat plot for the analysis between negative symptom and global-FA (excluding frontal brain regions) showing the influence of each site on the pooled association (y-axis) and the overall heterogeneity (x-axis) across sites.


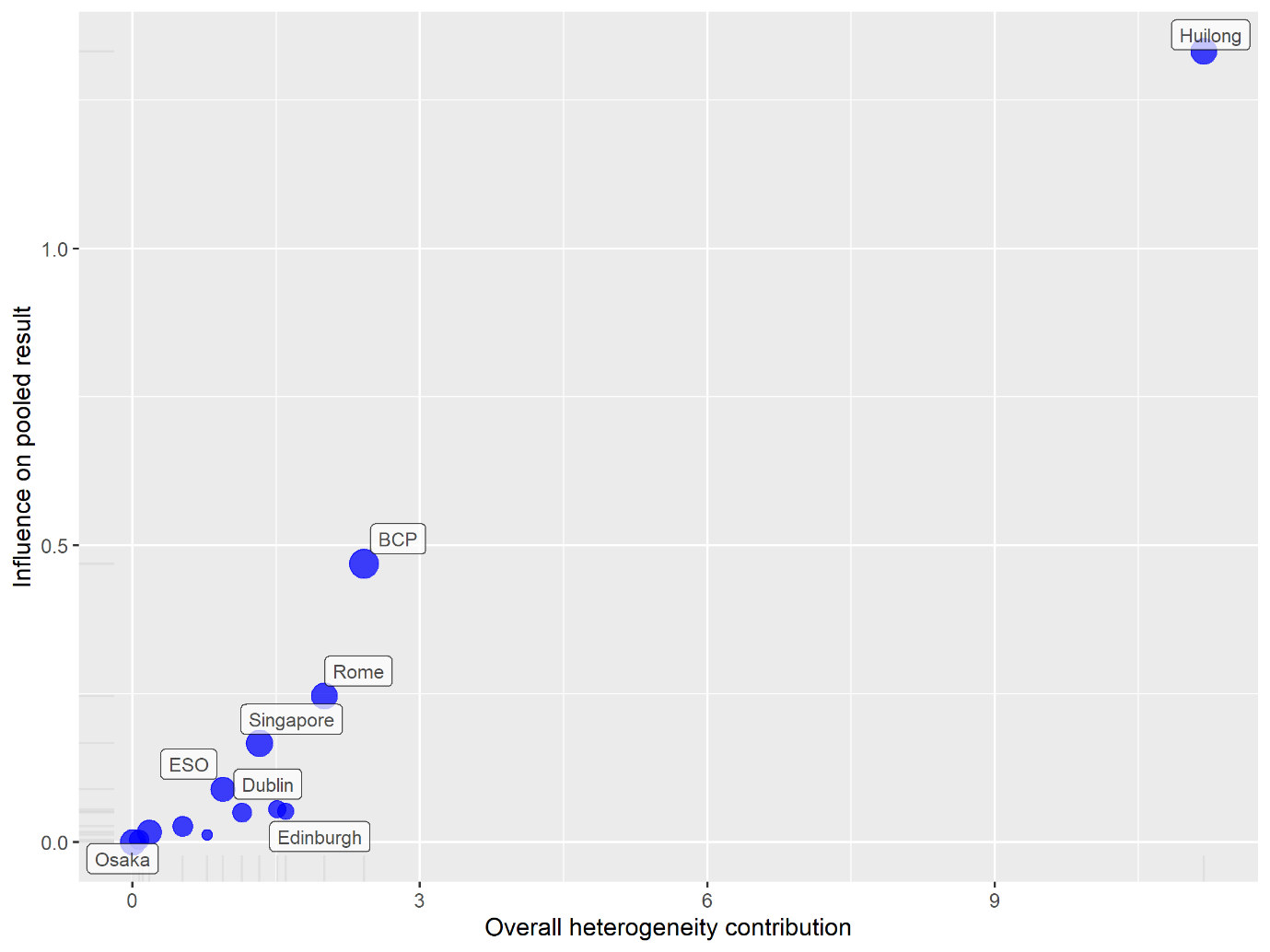


**Supplementary Figure 24.** Leave-One-Out analysis for the negative symptom and global-FA (excluding frontal regions) meta-analysis sorted by correlation. After omitting each of the 14 sites on a leave-one-out basis, the pooled correlation coefficient differs the most when Huilong is omitted (estimated r = -0.09 [-0.18, -0.01], I^2^ = 0%).


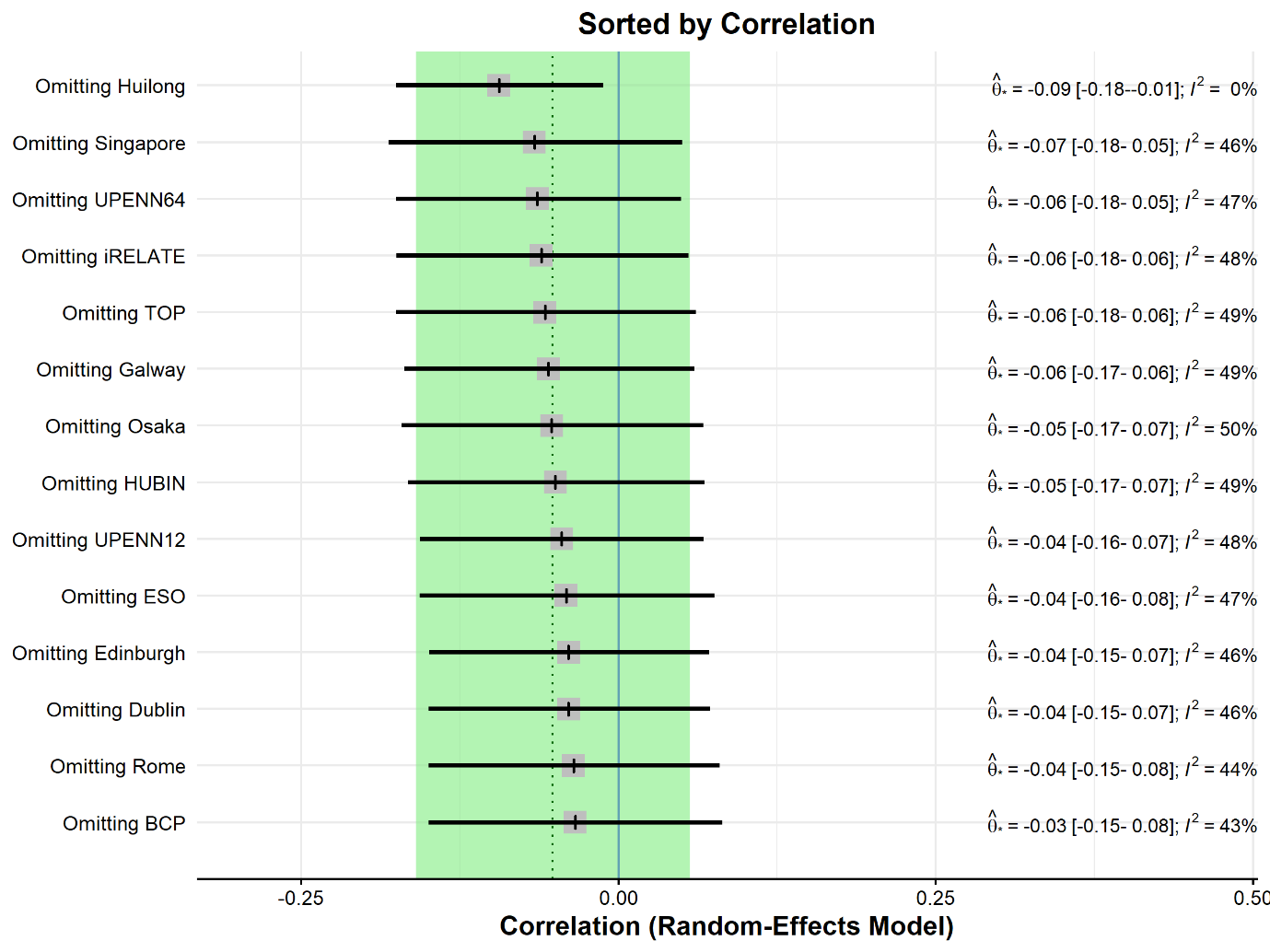


**Supplementary Figure 25.** Leave-One-Out analysis for the negative symptom and global-FA (excluding frontal regions) meta-analysis sorted by heterogeneity as measured by I^2^. After omitting each of the 14 sites on a leave-one-out basis, the heterogeneity between sites differs the most when Huilong is omitted (estimated r = -0.09 [-0.18, -0.01], I^2^ = 0%).


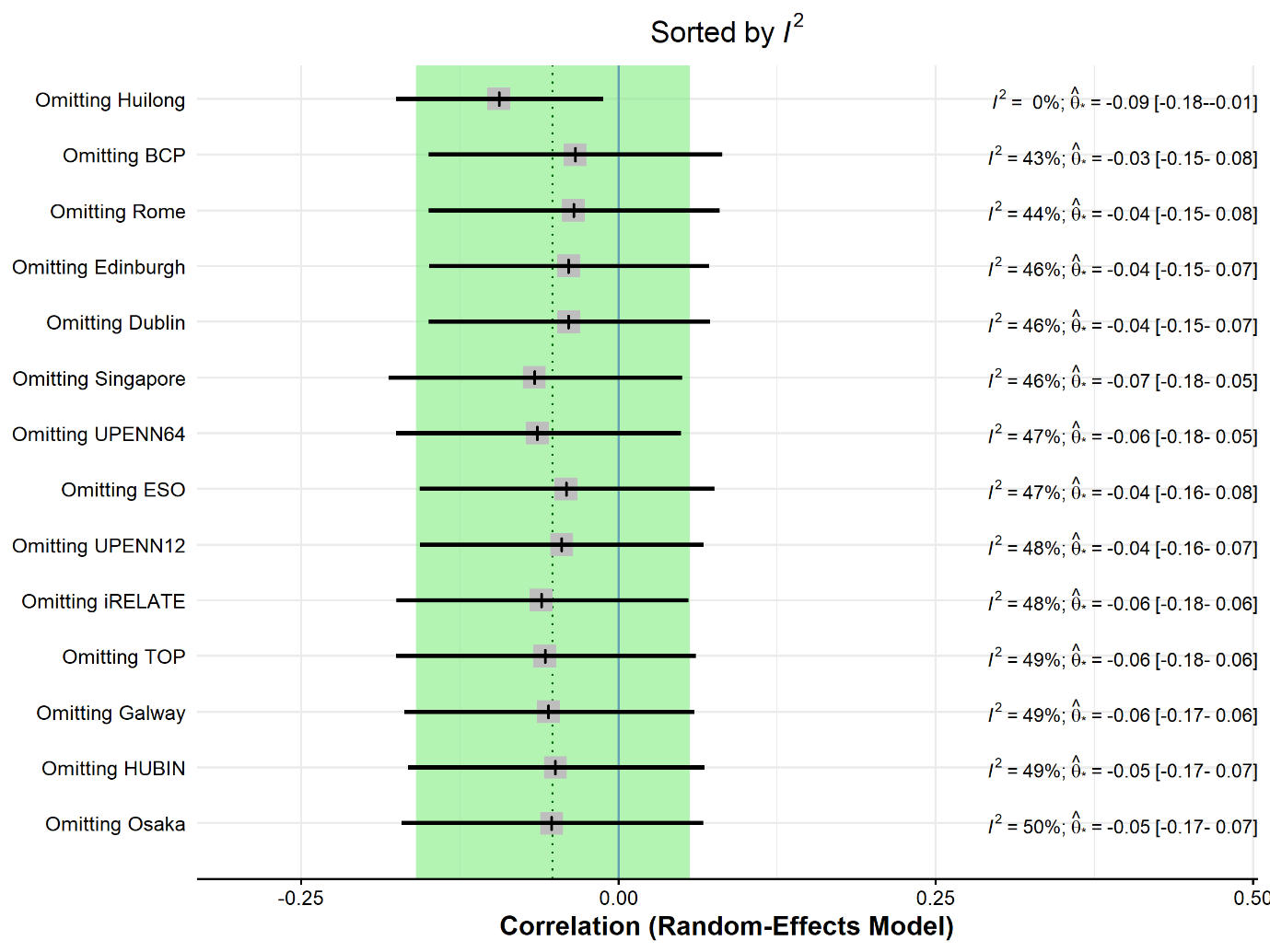


**Supplementary Figure 26.** Meta-analysis of negative symptoms and global-FA (excluding frontal regions) and removing Huilong from the analysis. The results show that there is a significant negative pooled association (r = -0.094, [-0.175, -0.012], *p* = 0.028) with a non-significant degree of residual heterogeneity (I^2^ = 0%, *p* = 0.51).


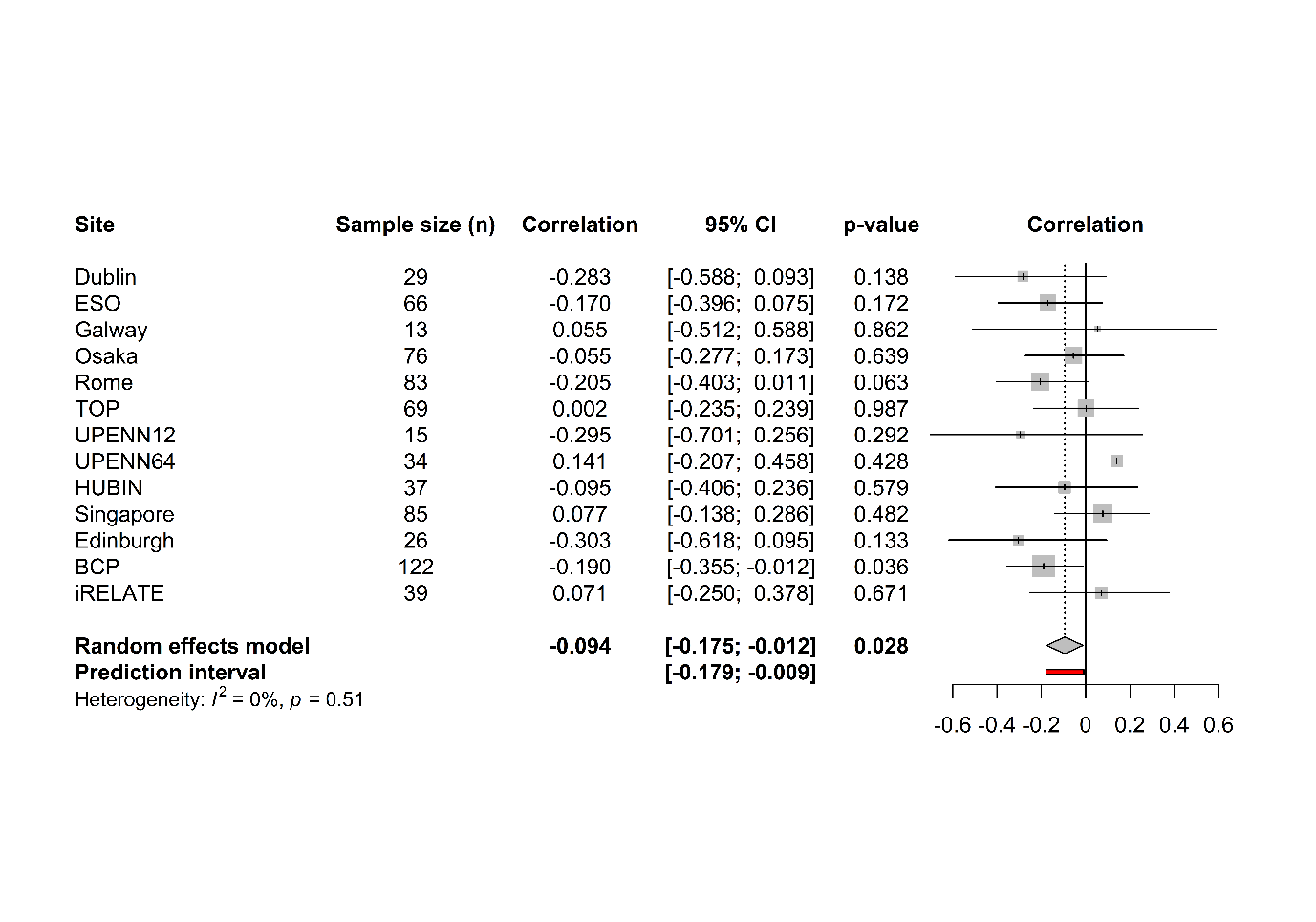


**Supplementary Analyses Exploring the Effects of Age and Sex on the FA-symptom associations**

**Supplementary Table 14A.** Heterogeneity results for the mixed-effects model between positive symptoms and temporal-FA across sites with age as the moderator.

| **Mixed Effects Model** | |
| --- | --- |
| **Measure of variability** | **Value** |
| tau^2^ | 0 (SE = 0.0070) |
| tau | 0 |
| I^2^ | 0.00% |
| H^2^ | 1 |
| R^2^ | 0.00% |

**Supplementary Table 14B.** Moderator results for the mixed-effects model between positive symptoms and temporal-FA across sites with age as the moderator.

|  | **estimate** | **se** | **tval** | **df** | **pval** | **LLCI** | **ULCI** |
| --- | --- | --- | --- | --- | --- | --- | --- |
| **intercept** | 0.1526 | 0.1526 | 0.9998 | 17 | 0.3314 | -0.1694 | 0.4745 |
| **mod1 (age)** | -0.0064 | 0.0041 | -1.5484 | 17 | 0.1399 | -0.0152 | 0.0023 |

**Supplementary Table 15A.** Heterogeneity results for the mixed-effects model between positive symptoms and temporal-FA across sites with sex (% males) as the moderator.

| **Mixed Effects Model** | |
| --- | --- |
| **Measure of variability** | **Value** |
| tau^2^ | 0 (0.0071) |
| tau | 0 |
| I^2^ | 0.00% |
| H^2^ | 1 |
| R^2^ | 0.00% |

**Supplementary Table 15B.** Moderator results for the mixed-effects model between positive symptoms and temporal-FA across sites with sex (% males) as the moderator.

|  | **estimate** | **se** | **tval** | **df** | **pval** | **LLCI** | **ULCI** |
| --- | --- | --- | --- | --- | --- | --- | --- |
| **intercept** | -0.0009 | 0.1633 | -0.0054 | 17 | 0.9957 | -0.3455 | 0.3437 |
| **mod1 (sex)** | -0.0013 | 0.0027 | -0.4935 | 17 | 0.628 | -0.0069 | 0.0043 |

**Supplementary Table 16A.** Heterogeneity results for the mixed-effects model between positive symptoms and global-FA across sites with age as the moderator.

| **Mixed Effects Model** | |
| --- | --- |
| **Measure of variability** | **Value** |
| tau^2^ | 0 (SE = 0.0070) |
| tau | 0 |
| I^2^ | 0.00% |
| H^2^ | 1 |
| R^2^ | 0.00% |

**Supplementary Table 16B.** Moderator results for the mixed-effects model between positive symptoms and global-FA across sites with age as the moderator.

|  | **estimate** | **se** | **tval** | **df** | **pval** | **LLCI** | **ULCI** |
| --- | --- | --- | --- | --- | --- | --- | --- |
| **intercept** | 0.1764 | 0.172 | 1.0258 | 17 | 9.3194 | -0.1865 | 0.5393 |
| **mod1 (age)** | -0.0068 | 0.0047 | -1.4455 | 17 | 0.1665 | -0.0166 | 0.0031 |

**Supplementary Table 17A.** Heterogeneity results for the mixed-effects model between positive symptoms and global-FA across sites with sex (% males) as the moderator.

| **Mixed Effects Model** | |
| --- | --- |
| **Measure of variability** | **Value** |
| tau^2^ | 0 (SE = 0.0071) |
| tau | 0 |
| I^2^ | 0.00% |
| H^2^ | 1 |
| R^2^ | 0.00% |

**Supplementary Table 17B.** Moderator results for the mixed-effects model between positive symptoms and global-FA across sites with sex (% males) as the moderator.

|  | **estimate** | **se** | **tval** | **df** | **pval** | **LLCI** | **ULCI** |
| --- | --- | --- | --- | --- | --- | --- | --- |
| **intercept** | 0.0531 | 0.1815 | 0.2923 | 17 | 0.7736 | -0.3299 | 0.436 |
| **mod1 (sex)** | -0.002 | 0.003 | -0.6802 | 17 | 0.5055 | -0.0083 | 0.0042 |

**Supplementary Figure 27.** *Age Stratification Analysis:* Meta-analysis of positive symptoms and temporal-FA for sites with a "young" or "middle" mean age (as calculated using a tertile split). The results show that there is a significant pooled association (r = -0.059, 95% CI [-0.116, -0.002], p = 0.0442), with a non-significant degree of residual heterogeneity (I^2^ = 0%, p = 0.9252).


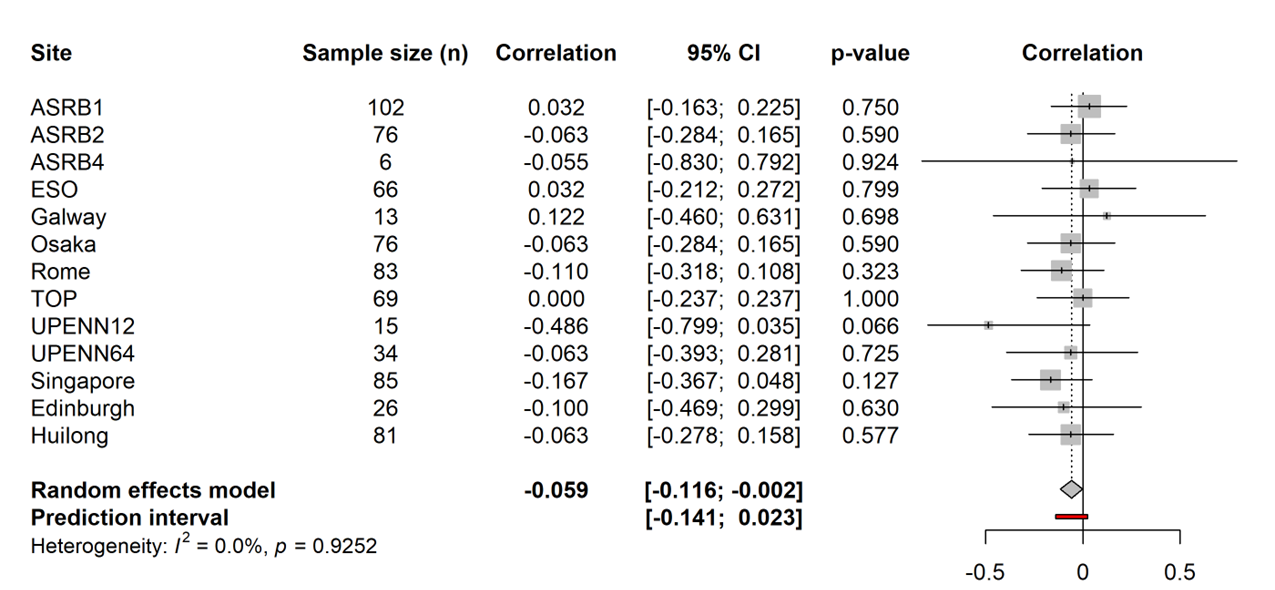


**Supplementary Figure 28.** *Age Stratification Analysis:* Meta-analysis of positive symptoms and temporal-FA for sites with a "middle" or "old" mean age (as calculated using a tertile split). The results show that there is a significant pooled association (r = -0.098, 95% CI [-0.184, -0.011], p = 0.0301), with a non-significant degree of residual heterogeneity (I^2^ = 0%, p = 0.5514).


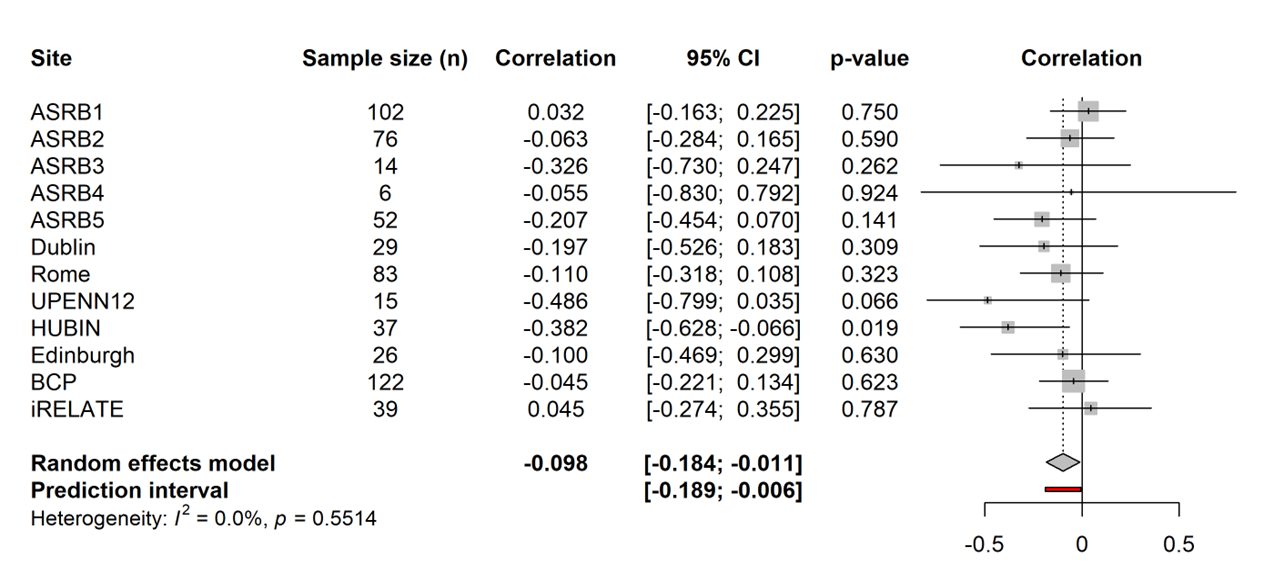


**Supplementary Figure 29.** *Age Stratification Analysis:* Meta-analysis of positive symptoms and global-FA for sites with a "young" or "middle" mean age (as calculated using a tertile split). The results show that there is a non-significant pooled association (r = -0.056, 95% CI [-0.118, 0.006], p = 0.0730) across sites, with a non-significant degree of residual heterogeneity (I^2^ = 0%, p = 0.8721).


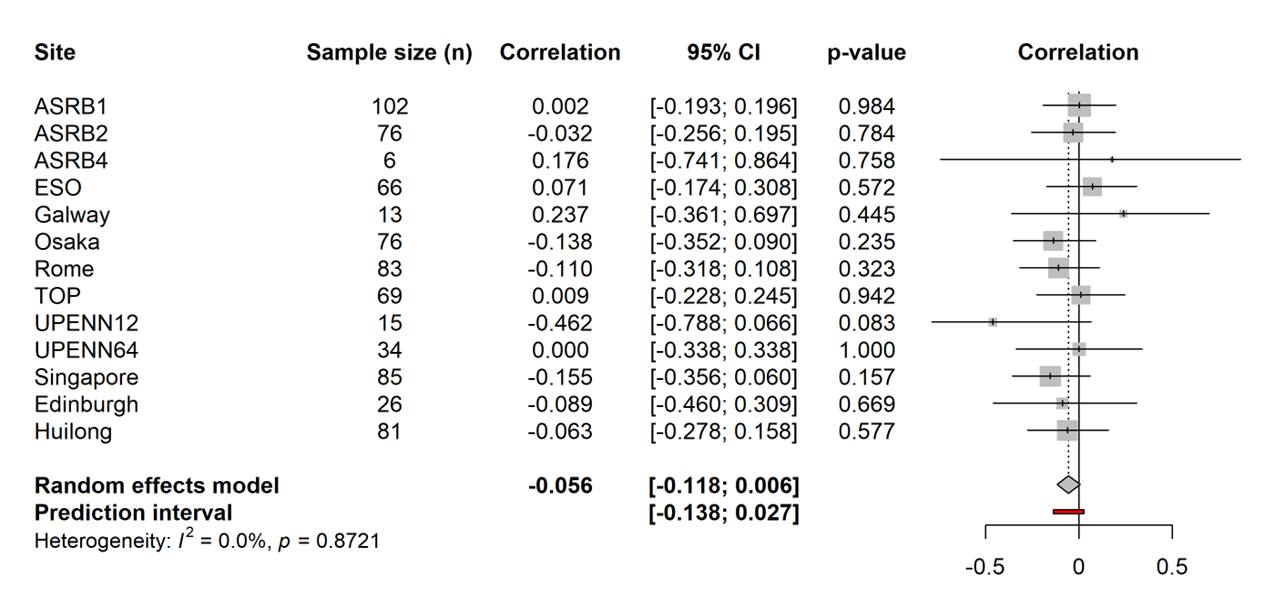


**Supplementary Figure 30.** *Age Stratification Analysis:* Meta-analysis of positive symptoms and global-FA for sites with a "middle" or "old" mean age (as calculated using a tertile split). The results show that there is a non-significant pooled association (r = -0.084, 95% CI [-0.179, 0.012], p = 0.0789) across sites, with a non-significant degree of residual heterogeneity (I^2^ = 4.6%, p = 0.3996).


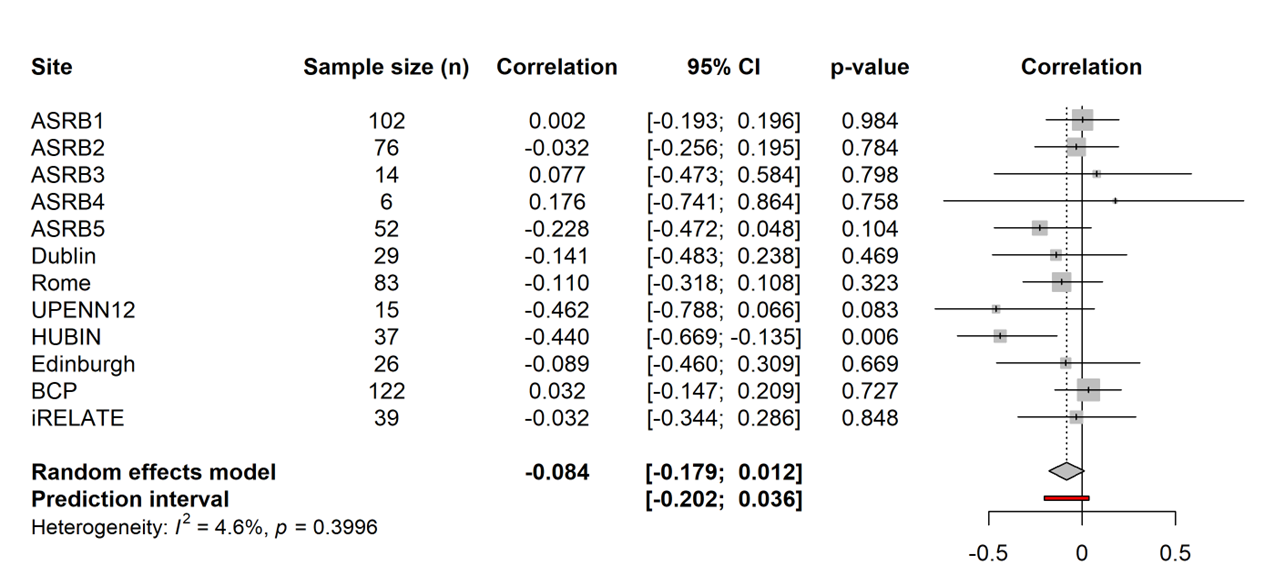


**Supplementary Table 18A.** Heterogeneity results for the mixed-effects model between negative symptoms and frontal-FA across sites with age as the moderator.

| **Mixed Effects Model** | |
| --- | --- |
| **Measure of variability** | **Value** |
| tau^2^ | 0 (SE = 0.0082) |
| tau | 0 |
| I^2^ | 0.00% |
| H^2^ | 1 |
| R^2^ | 100.00% |

**Supplementary Table 18B.** Moderator results for the mixed-effects model between negative symptoms and frontal-FA across sites with age as the moderator. *Note: ** p* < 0.01, **** p* < 0.001

|  | **estimate** | **se** | **tval** | **df** | **pval** | **LLCI** | **ULCI** |
| --- | --- | --- | --- | --- | --- | --- | --- |
| **intercept** | 0.6375 | 0.1833 | 3.4781 | 12 | 0.0046*** | 0.2381 | 1.0368 |
| **mod1 (age)** | -0.0204 | 0.0051 | -4.015 | 12 | 0.0017** | -0.0314 | -0.0093 |

**Supplementary Table 19A.** Heterogeneity results for the mixed-effects model between negative symptoms and frontal-FA across sites with sex (% males) as the moderator.

| **Mixed Effects Model** | |
| --- | --- |
| **Measure of variability** | **Value** |
| tau^2^ | 0.0214 (SE = 0.0175) |
| tau | 0.1464 |
| I^2^ | 51.98% |
| H^2^ | 2.08 |
| R^2^ | 0.00% |

**Supplementary Table 19B.** Moderator results for the mixed-effects model between negative symptoms and frontal-FA across sites with sex (% males) as the moderator.

|  | **estimate** | **se** | **tval** | **df** | **pval** | **LLCI** | **ULCI** |
| --- | --- | --- | --- | --- | --- | --- | --- |
| **intercept** | -0.0358 | 0.298 | -0.12 | 12 | 0.9065 | -0.6851 | 0.6136 |
| **mod1 (sex)** | -0.0009 | 0.0049 | -0.1748 | 12 | 0.8642 | -0.0116 | 0.0099 |

**Supplementary Table 20A.** Heterogeneity results for the mixed-effects model between negative symptoms and global-FA across sites with age as the moderator.

| **Mixed Effects Model** | |
| --- | --- |
| **Measure of variability** | **Value** |
| tau^2^ | 0.0037 (SE = 0.0097) |
| tau | 0.061 |
| I^2^ | 15.66% |
| H^2^ | 1.19 |
| R^2^ | 83.73% |

**Supplementary Table 20B.** Moderator results for the mixed-effects model between negative symptoms and global-FA across sites with age as the moderator. *Note: * p* < 0.05, **** p* < 0.01

|  | **estimate** | **se** | **tval** | **df** | **pval** | **LLCI** | **ULCI** |
| --- | --- | --- | --- | --- | --- | --- | --- |
| **intercept** | 0.0602 | 0.2161 | 2.7857 | 12 | 0.0165* | 0.1312 | 1.0728 |
| **mod1 (age)** | -0.0192 | 0.006 | -3.2181 | 12 | 0.0074** | -0.0322 | -0.0062 |

**Supplementary Table 21A.** Heterogeneity results for the mixed-effects model between negative symptoms and global-FA across sites with sex (% males) as the moderator.

| **Mixed Effects Model** | |
| --- | --- |
| **Measure of variability** | **Value** |
| tau^2^ | 0.0265 (SE = 0.0198) |
| tau | 0.1628 |
| I^2^ | 57.23% |
| H^2^ | 2.34 |
| R^2^ | 0.00% |

**Supplementary Table 21B.** Moderator results for the mixed-effects model between negative symptoms and global-FA across sites with sex (% males) as the moderator.

|  | **estimate** | **se** | **tval** | **df** | **pval** | **LLCI** | **ULCI** |
| --- | --- | --- | --- | --- | --- | --- | --- |
| **intercept** | -0.0174 | 0.3137 | -0.0556 | 12 | 0.9566 | -0.7009 | 0.6661 |
| **mod1 (age)** | -0.0011 | 0.0052 | -0.2171 | 12 | 0.8317 | -0.0124 | 0.0102 |

**Supplementary Figure 31.** *Age Stratification Analysis:* Meta-analysis of negative symptoms and frontal-FA for sites with a "young" or "middle" mean age (as calculated using a tertile split). The results show that there is a non-significant pooled association (r = -0.078, 95% CI [-0.186, 0.032], p = 0.1395), with a non-significant degree of residual heterogeneity (I^2^ = 0%, p = 0.4375).


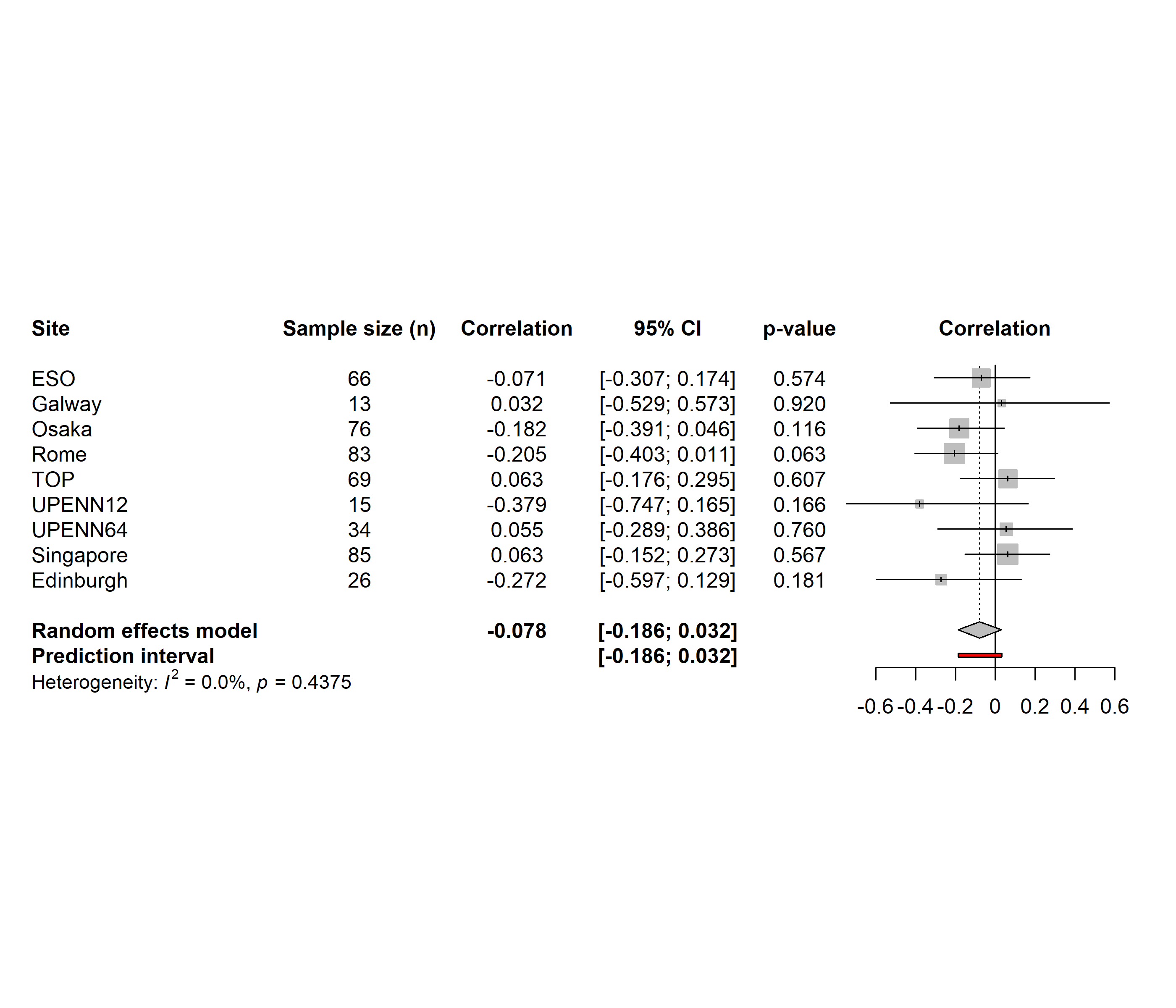


**Supplementary Figure 32.** *Age Stratification Analysis:* Meta-analysis of negative symptoms and frontal-FA for sites with a "middle" or “old” mean age (as calculated using a tertile split). The results show that there is a significant inverse pooled association (r = -0.217, 95% CI [-0.308, -0.121], *p* = 0.0011), with a non-significant degree of residual heterogeneity (I^2^ = 0%, *p* = 0.6761).


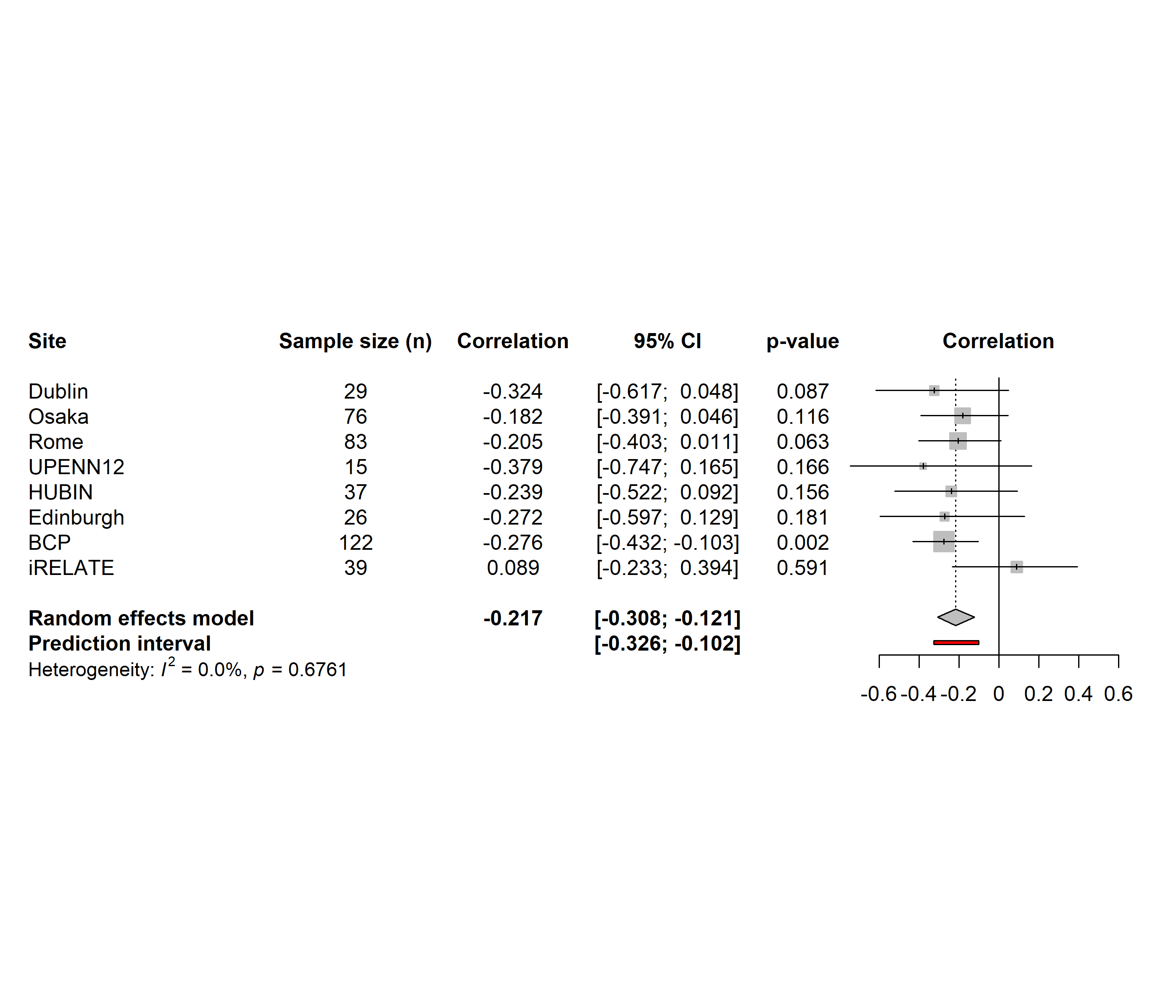


**Supplementary Figure 33.** *Age Stratification Analysis:* Meta-analysis of negative symptoms and global-FA for sites with a “young” or "middle" mean age (as calculated using a tertile split). The results show that there is a non-significant pooled association (r = -0.096, 95% CI [-0.219, 0.030], p = 0.1162), with a non-significant degree of residual heterogeneity (I^2^ = 0%, p = 0.2534).


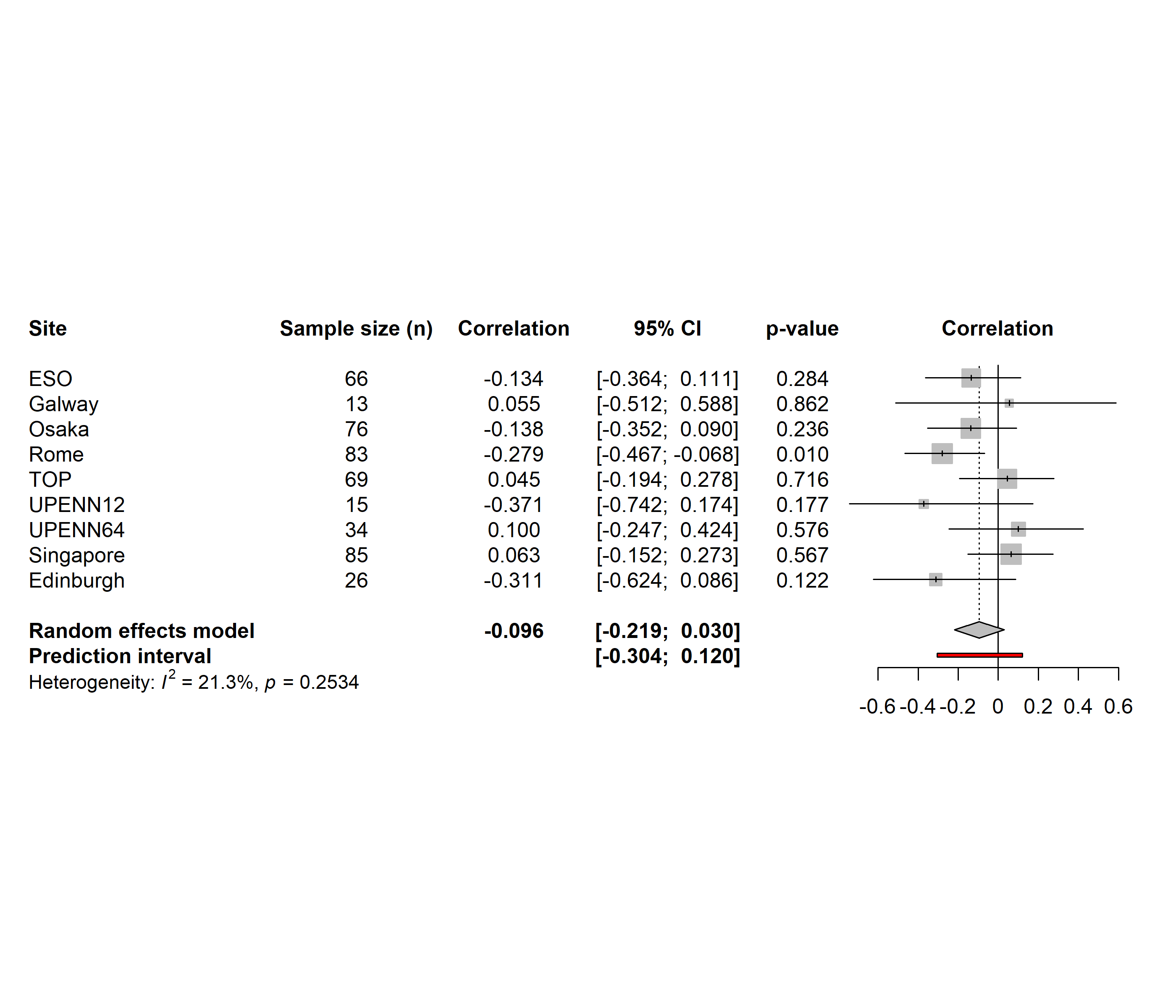


**Supplementary Figure 34.** *Age Stratification Analysis:* Meta-analysis of negative symptoms and global-FA for sites with a "middle" or “old” mean age (as calculated using a tertile split). The results show that there is a significant inverse pooled association (r = -0.212, 95% CI [-0.306, -0.113], p = 0.0015), with a non-significant degree of residual heterogeneity (I^2^ = 0%, p = 0.6389).


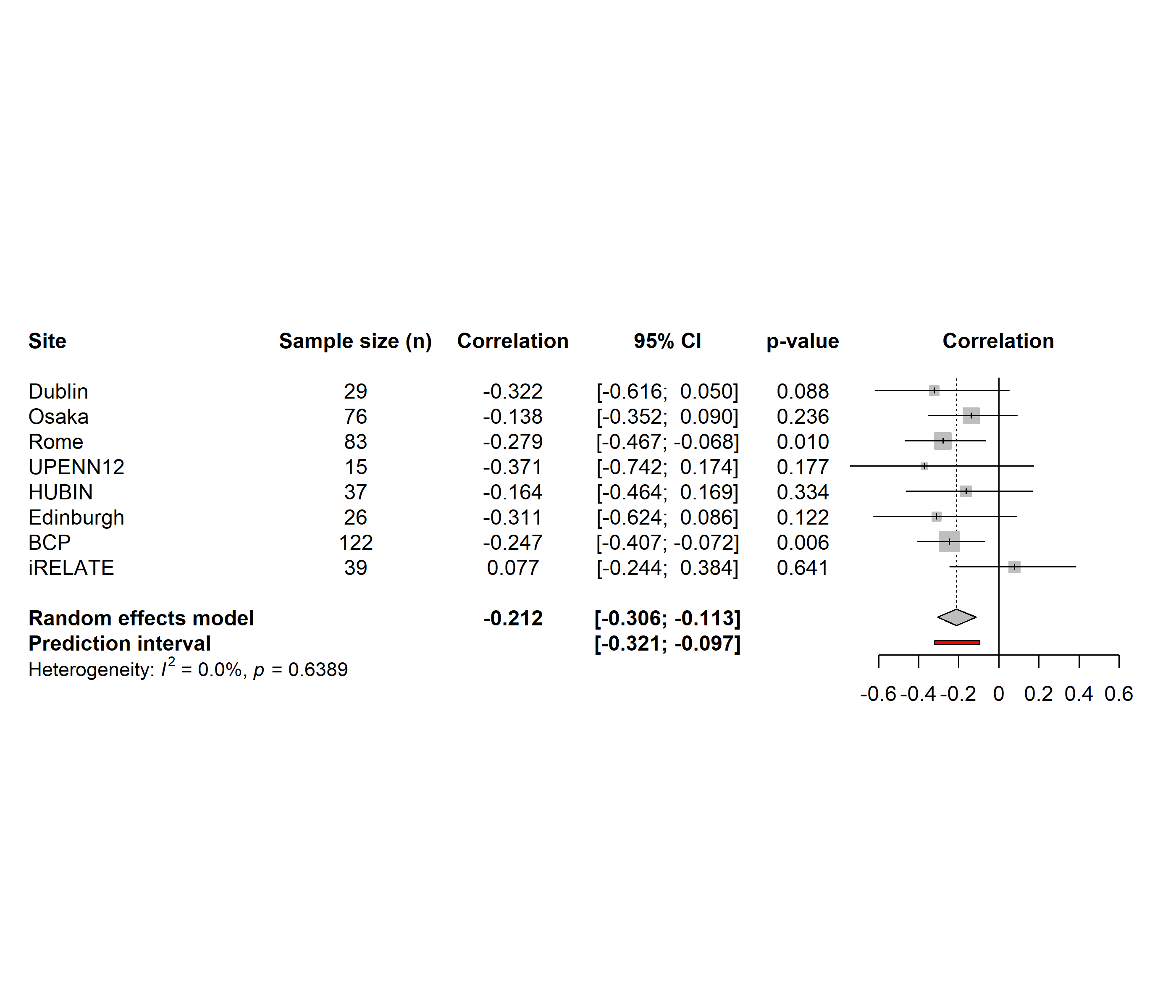


**Supplementary Analysis excluding ASRB1-5 in the positive symptom analyses**

**Supplementary Figure 35.** Meta-analysis of positive symptoms and temporal-FA with sites ASRB1-5 excluded. The results show that there is a significant pooled association (r = -0.085, 95% CI [-0.152, -0.017], p = 0.0183), with a non-significant degree of residual heterogeneity (I^2^ = 0%, p = 0.7391).


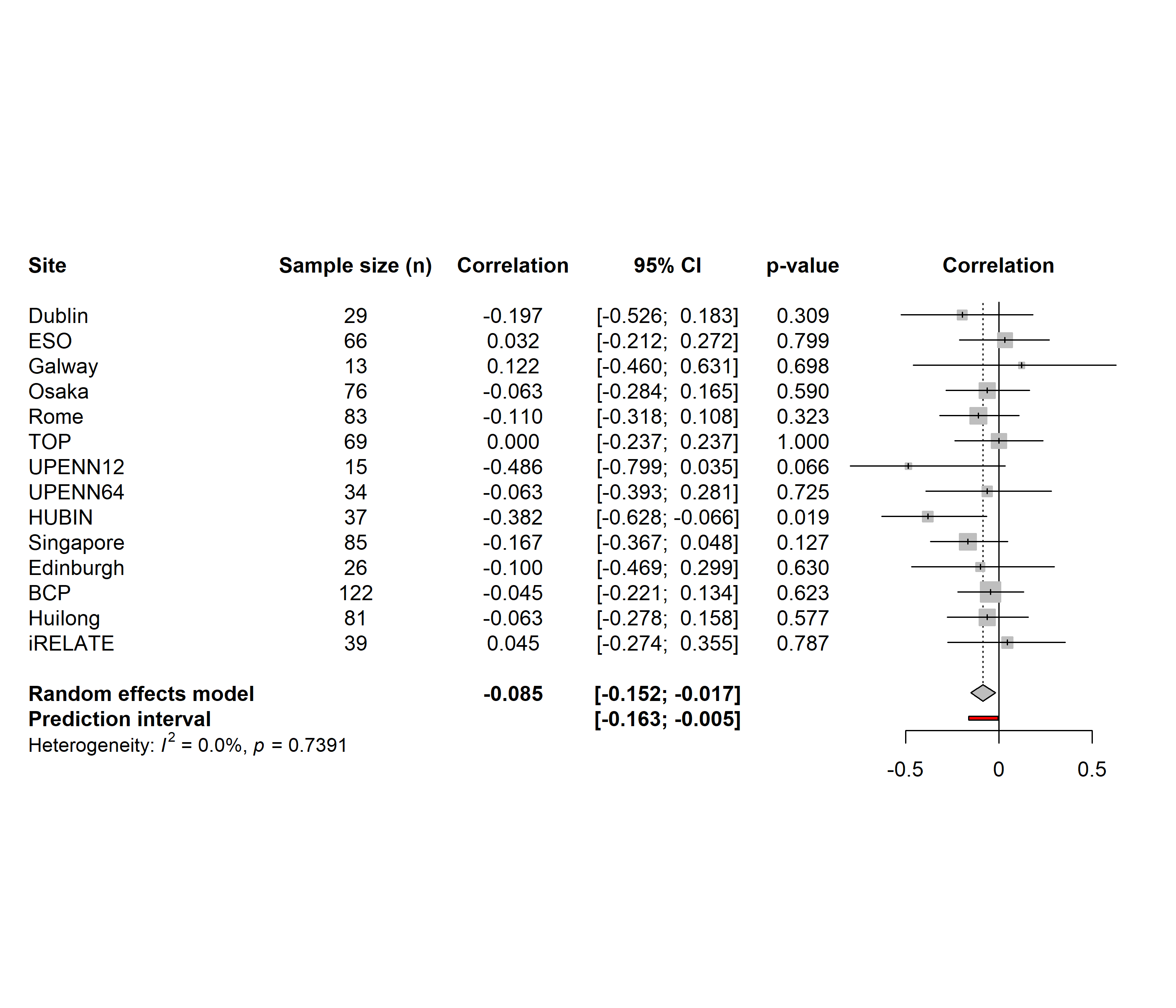


**Supplementary Figure 36.** Meta-analysis of positive symptoms and global-FA with sites ASRB1-5 excluded. The results show that there is a non-significant pooled association (r = -0.074, 95% CI [-0.153, 0.006], p = 0.0661) across sites, with a non-significant degree of residual heterogeneity (I^2^ = 0.1%, p = 0.4463).


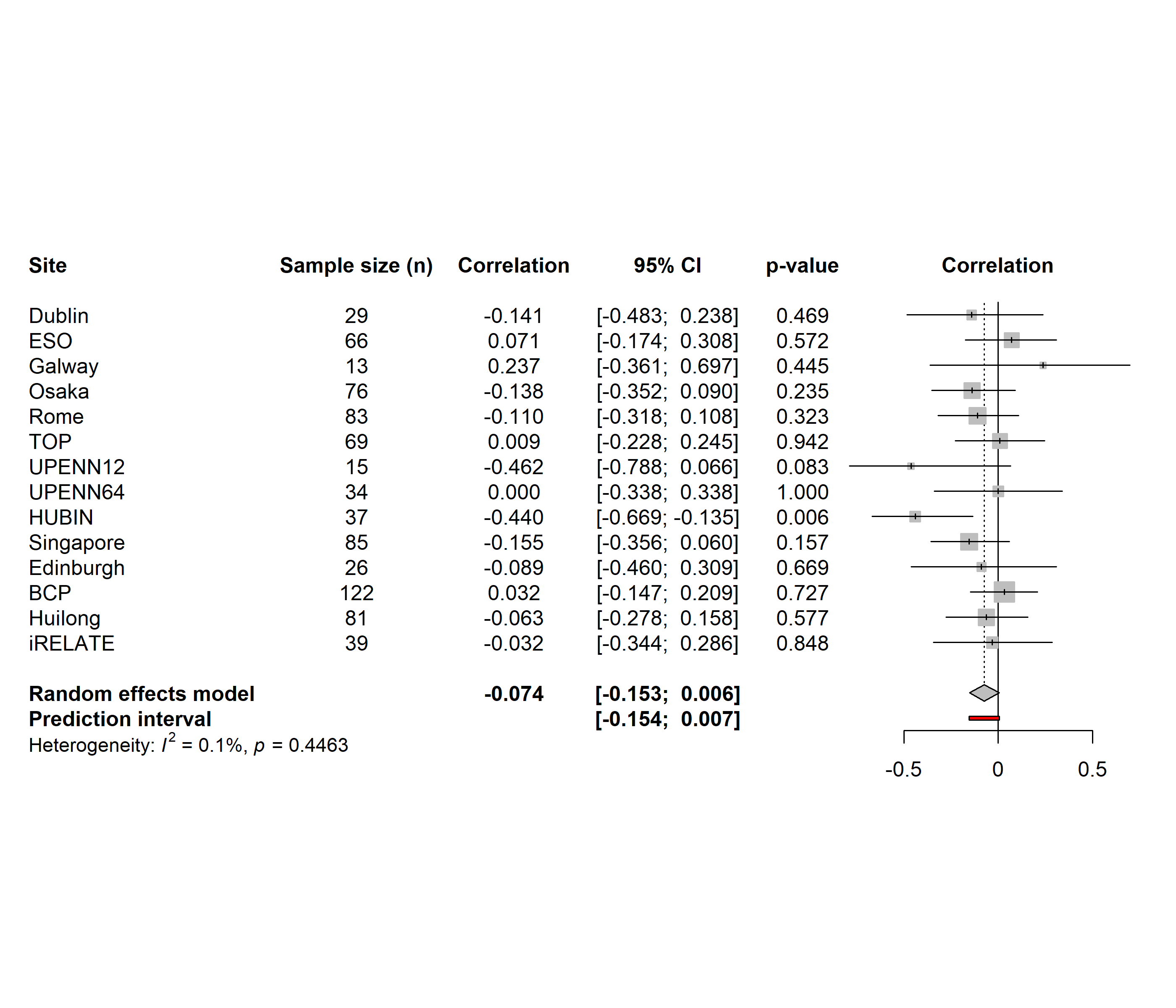

Supplement: Supplementary file 1 — Supplementary Materials for the Manuscript [file 41537_2026_728_MOESM1_ESM.docx]
